# High-throughput allele-specific expression across 250 environmental conditions

**DOI:** 10.1101/gr.209759.116

**Published:** 2016-12

**Authors:** Gregory A. Moyerbrailean, Allison L. Richards, Daniel Kurtz, Cynthia A. Kalita, Gordon O. Davis, Chris T. Harvey, Adnan Alazizi, Donovan Watza, Yoram Sorokin, Nancy Hauff, Xiang Zhou, Xiaoquan Wen, Roger Pique-Regi, Francesca Luca

**Affiliations:** 1 Center for Molecular Medicine and Genetics, Wayne State University, Detroit, Michigan 48201, USA;; 2 Department of Obstetrics and Gynecology, Wayne State University, Detroit, Michigan 48201, USA;; 3 Department of Biostatistics, University of Michigan, Ann Arbor, Michigan 48109, USA

## Abstract

Gene-by-environment (GxE) interactions determine common disease risk factors and biomedically relevant complex traits. However, quantifying how the environment modulates genetic effects on human quantitative phenotypes presents unique challenges. Environmental covariates are complex and difficult to measure and control at the organismal level, as found in GWAS and epidemiological studies. An alternative approach focuses on the cellular environment using in vitro treatments as a proxy for the organismal environment. These cellular environments simplify the organism-level environmental exposures to provide a tractable influence on subcellular phenotypes, such as gene expression. Expression quantitative trait loci (eQTL) mapping studies identified GxE interactions in response to drug treatment and pathogen exposure. However, eQTL mapping approaches are infeasible for large-scale analysis of multiple cellular environments. Recently, allele-specific expression (ASE) analysis emerged as a powerful tool to identify GxE interactions in gene expression patterns by exploiting naturally occurring environmental exposures. Here we characterized genetic effects on the transcriptional response to 50 treatments in five cell types. We discovered 1455 genes with ASE (FDR < 10%) and 215 genes with GxE interactions. We demonstrated a major role for GxE interactions in complex traits. Genes with a transcriptional response to environmental perturbations showed sevenfold higher odds of being found in GWAS. Additionally, 105 genes that indicated GxE interactions (49%) were identified by GWAS as associated with complex traits. Examples include *GIPR*–caffeine interaction and obesity and include *LAMP3*–selenium interaction and Parkinson disease. Our results demonstrate that comprehensive catalogs of GxE interactions are indispensable to thoroughly annotate genes and bridge epidemiological and genome-wide association studies.

For complex traits, a mismatch between genotype and environment can cause a higher disease risk. However, it is generally difficult to determine the relevant environmental factors to measure in order to study gene-by-environment (GxE) interactions. Consequently, some of the genetic effect sizes measured in GWAS may be underestimated when the relevant environmental factors are not controlled. Molecular phenotypes measured in tightly controlled cellular environments provide a more tractable setting in which we can study GxE interactions simplifying both complex phenotypes and environments (Fig. 1A). The cellular environment is determined by the complex of stimuli (e.g., hormonal and metabolic) to which a cell is exposed, can be defined as an agent that can potentially change the state of the cell, and is measurable at the molecular level. Examples include, agents secreted by nearby cells, hormones and metabolites secreted by other organs, pollutants, drugs, or micronutrients absorbed by the organisms. For example, physical or emotional environmental stressors alter blood glucocorticoid levels, which induce significant cellular changes in global gene expression patterns mediated through glucocorticoid receptor (GR) activation (Grundberg et al. 2011; Luca et al. 2013). Response expression quantitative trait loci (reQTL) mapping studies found that SNPs associated with specific immune traits are enriched for infection reQTL and for expression quantitative trait loci (eQTL) identified only in infected cells (Barreiro et al. 2012; Fairfax et al. 2014; Lee et al. 2014). However, eQTL mapping requires a large number of samples, thus limiting the number of cellular environments that can be analyzed in parallel. While association mapping compares genotypic effects across individuals that have different genetic backgrounds, allele-specific expression (ASE) approaches compare allelic effects within individuals, thereby controlling for genetic background and cellular environment. Currently, ASE approaches represent the most effective assay to quantify *cis*-regulatory variants within a defined cellular environment and to control for *trans*-acting modifiers of gene expression, such as genotype at other loci (Kasowski et al. 2010; McDaniell et al. 2010; Pastinen 2010; Skelly et al. 2011; Cowper-Sal·lari et al. 2012; Reddy et al. 2012; McVicker et al. 2013; Buil et al. 2014; Hasin-Brumshtein et al. 2014; Kukurba et al. 2014; Knowles et al. 2015; Kumasaka et al. 2016).

**Figure 1.**
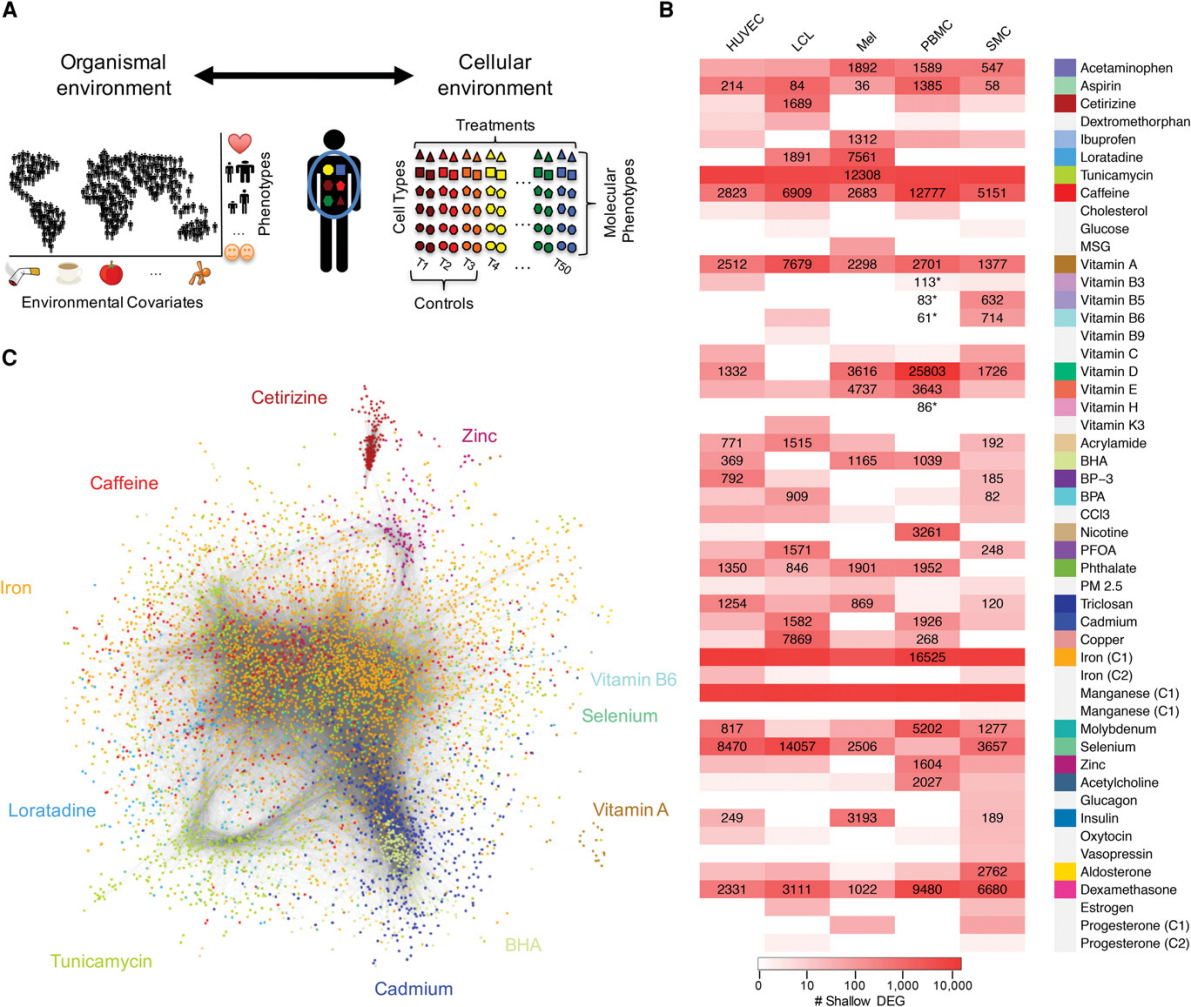
Overview of gene expression response. (*A*) Schematic of experimental design and rationale. Our approach uses specific treatment conditions as tightly controlled proxy for the organism environment and measures molecular phenotypes, such as gene expression, to infer genetic and molecular mechanisms for complex traits. (*B*) Heatmap of differential gene expression. Shown for each cell type (columns) and treatment (rows) combination are the number of differentially expressed genes (10% FDR and |log_2_FC| > 0.25). The shade of red indicates the number of differentially expressed genes from an initial screening step (see Supplemental Texts 5 and 8.1). Cellular environments with a strong response were further sequenced to a higher depth (>58 M reads, 113 M on average), and the number of differentially expressed genes is indicated by the text. Environments marked with an asterisk were chosen to confirm that treatments with a small response from the shallow sequencing data similarly have a small response when deep sequenced. Colors next to treatment names represent treatments chosen for deep sequencing. Gray indicates treatments that were not deep sequenced. (*C*) Global coexpression network inferred using weighted gene correlation network analysis (WGCNA) on 14,535 genes. Each dot represents a gene. Each module is assigned a color based on the treatment with the highest eigengene *t*-value. Note that colors representing treatments are consistently used across all the figures.

Here we developed a high-throughput in vitro system to characterize the response to tightly controlled environmental exposures. We then used ASE analysis to identify GxE interactions in individual samples at heterozygous sites. We focused on 50 treatments (Supplemental Table S1) in five cell types (250 conditions) across 15 individuals (three samples per cell type), including paired vehicle-controls. These treatments represent the cellular counterparts of a range of organismal exposures (Supplemental Table S1). We broadly grouped the treatments into six categories: steroid hormones, peptide hormones, metal ions, dietary components, common drugs, and environmental contaminants. For each treatment, we used the metabolically active form detected in the bloodstream at the highest physiological concentration reported by the Mayo Clinic (http://www.mayomedicallaboratories.com) or the CDC (http://www.cdc.gov/biomonitoring/), as available. Our goal is to identify GxE interactions across these conditions and characterize their roles in complex traits.

## Results

### High-throughput characterization of transcriptional responses

Literature reports on transcriptional responses for many treatments across cell types are often contradictory or nonexistent. To characterize transcriptional responses to 50 environmental perturbations (Supplemental Table S1) in five cell types (250 conditions), we utilized a high-throughput two-step RNA-seq approach (Supplemental Fig. S2; Moyerbrailean et al. 2015). In step one, we used shallow RNA-seq (8.2 million reads/sample on average) (Supplemental Table S2) and DESeq2 (Love et al. 2014) to coarsely characterize global changes in gene expression (Supplemental Fig. S3; Supplemental Table S3). We considered only treatment-by-cell-type combinations with more than 80 differentially expressed (DE) genes detected at 10% FDR and corresponding to |log_2_FC| > 0.25. We found eight treatments that induced gene expression changes across all cell types, such as dexamethasone and vitamin A, while other treatments had a cell-type–specific effect, such as vitamin B6 in peripheral blood mononuclear cells (PBMCs) (Fig. 1B). Of the 50 treatments, 16 did not induce significant changes in gene expression in any cell type. We excluded a few outlier response conditions (see Supplemental Methods 8.2). By using these criteria, we selected 89 conditions (35 treatments across five cell types and three individuals) corresponding to 297 RNA-seq libraries and resequenced them to 130 M reads per sample on average (Supplemental Table S4) in step two.

### Treatment-specific gene coexpression network

In step two, we used deep sequencing data and weighted gene correlation network analysis (WGCNA) (Langfelder and Horvath 2008) to infer the global gene coexpression network across all samples and environments (Fig. 1C). This network comprised 87 modules, which grouped genes with similar expression patterns that may be coregulated in a concerted way. The largest module contained 1456 genes, and the median module size was 42 genes. We assigned a representative treatment to each module based on the most significant treatment effect size on gene expression (Supplemental Fig. S11), and this allowed us to identify clusters of genes that represent treatment-specific responses. For example, two modules were each associated with treatment conditions in opposite directions: module 30 (vitamin D in HUVECs and PBMCs) and module 22 (caffeine and aspirin in smooth muscle cells [SMCs]) (Supplemental Figs. S12, S13). These results suggest that analysis of transcriptional responses to a large number of treatments in parallel can identify gene regulatory networks that mediate divergent effects that depend on specific cell type or treatment conditions.

### Analysis of ASE

**Figure 2.**
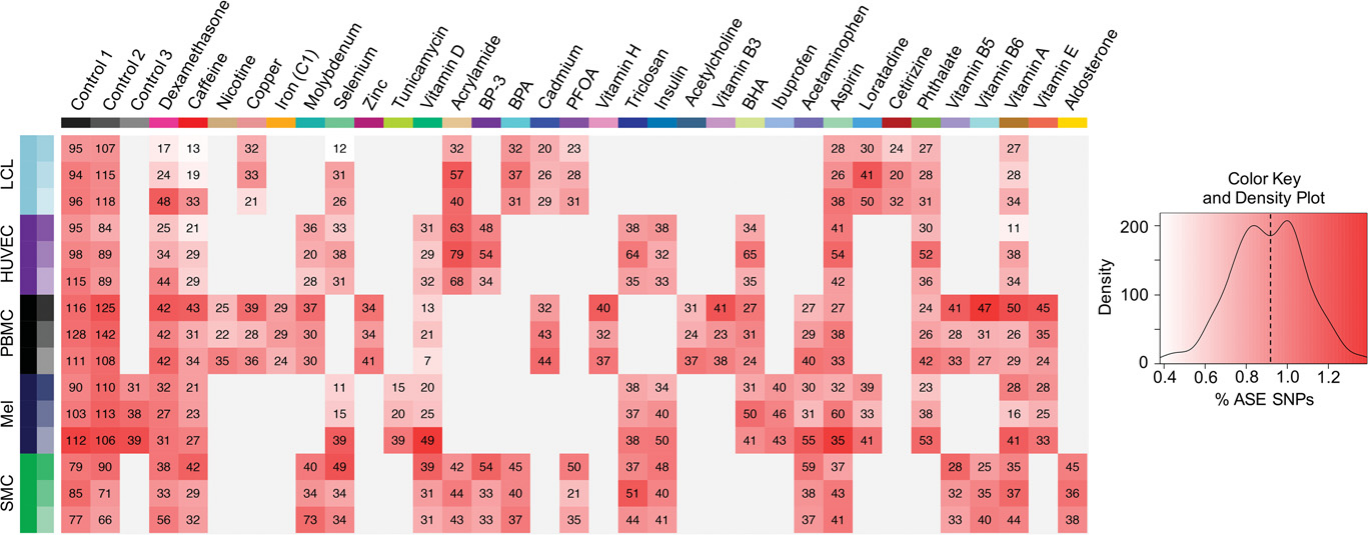
Heatmap of allele-specific expression (ASE). For each individual (row) and treatment (column) we list the number of SNPs displaying ASE (as determined in QuASAR [quantitative allele-specific analysis of reads] at 10% FDR). The shade of red represents the fraction of ASE SNPs to the number of heterozygous SNPs tested (% ASE SNPs) in a given sample and condition. The dotted line on the density plot indicates the average % ASE SNPs across all individual samples and conditions.

We used QuASAR (quantitative allele-specific analysis of reads) to identify genes with evidence of ASE. QuASAR (Harvey et al. 2015) identifies heterozygous genotypes and uses a beta binomial distribution to infer ASE in RNA-seq data. In the 89 treatment conditions, we identified 11,305 instances of ASE (10% FDR) (Fig. 2), corresponding to 1455 unique ASE genes. In an individual sample, 0.92% of expressed genes with heterozygous SNPs are ASE genes, on average.

The ASE analysis was performed on all expressed genes and was not limited to DE genes as some genes may not change total expression level. When we consider all ASE genes in our data set, 92% were also identified with eQTL in the Genotype-Tissue Expression (GTEx) Project. Thus, similar to other ASE studies (Buil et al. 2014), we were able to capture genes with regulatory variants that were previously identified at baseline. Additionally, many of the genes identified in GTEx with eQTL may have unknown latent environmental components modulating their expression.

### Analysis of GxE interactions

**Figure 3.**
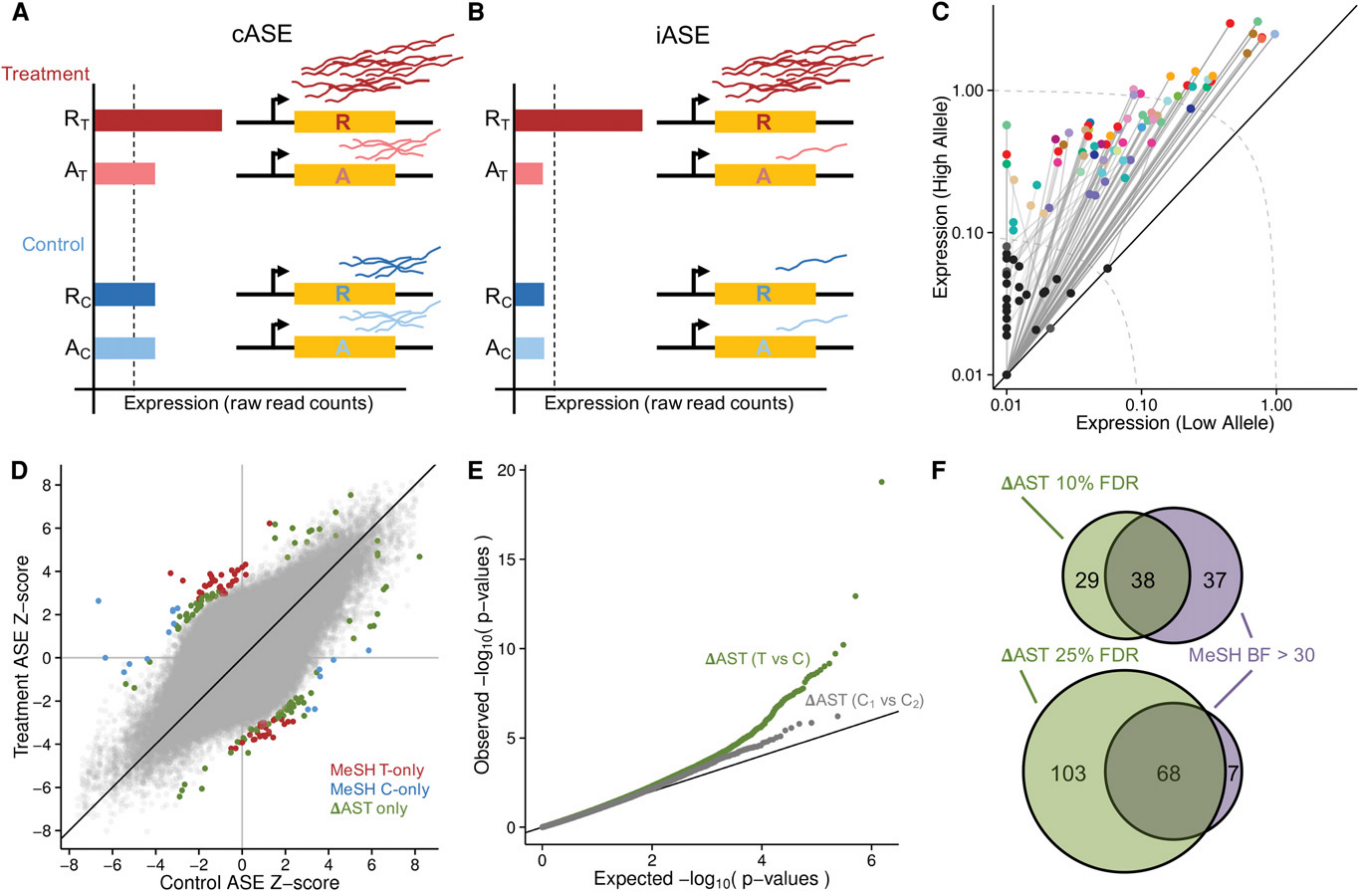
Gene–environment interactions. (*A*,*B*) Two types of gene–environment interactions: conditional ASE (*A*) and induced ASE (*B*). Treatment conditions are in red and control conditions in blue, with the shade (dark/light) representing the allele (reference/alternate). In this example of cASE, there is an imbalance of expression between the two alleles in the treatment condition, while the control shows balanced expression. iASE is defined by an imbalance of expression between the two alleles in the treatment condition and by expression below detectable levels (dotted line) in the control condition. (*C*) Plot of all iASE SNPs detected. Each iASE SNP is represented as two points (representing treatment and control expression) connected by a line (representing the fold-change between conditions). SNPs are plotted based on the expression (TPM [tags per million]) of each allele, with the higher expressed allele in the treatment on the *y*-axis and the lower allele on the *x*-axis. Points are colored by treatment (controls are black and gray), and the dotted lines represent constant expression levels 0.1, 1, and 10. For ease of display, expression of SNPs <0.01 have been set to 0.01. (*D*) Scatter plot of the *Z*-scores in the paired treatment and control samples for all SNPs tested for cASE. Colored points indicate those displaying cASE: Red is SNPs identified by meta-analysis of subgroup heterogeneity (MeSH) as having cASE in the treatment, blue is SNPs identified by MeSH as having cASE in the control, and green is SNPs identified by ΔAST (differential allele-specific test) that were not identified by MeSH. (*E*) QQ-plot of *P*-values for cASE identified with the ΔAST method for treatment versus control (green line) and Control 1 versus Control 2 (gray line). (*F*) Venn diagrams showing the number of cASE SNPs identified by two methods: MeSH and ΔAST at different empirically estimated FDR thresholds.

Next, we characterized GxE interactions on gene expression by analyzing ASE differences between treatment and control. Reliably estimating ASE effect sizes required a significant amount of reads for detecting condition-specific ASE (cASE) (see Fig. 3A; Supplemental Fig. S15). However, some genes had very low expression levels in the control condition and high expression with ASE following treatment, suggesting that expression of these genes would be induced by a specific treatment. For these cases, ASE in the control condition cannot be defined accurately. We denoted this phenomenon as induced ASE (iASE) (see Fig. 3B), which indicated cases when the ASE was only observed in genes induced by the treatment. Studies that only consider baseline eQTL or ASE may fail to characterize or may mischaracterize genes with iASE if the relevant environmental stimulus is present as latent exposure. We identified 75 iASE SNPs (10% FDR) corresponding to 60 unique genes (Fig. 3C). The genetic effect in these iASE SNPs is slightly stronger than that of baseline ASE (Supplemental Fig. S17).

When we can reliably measure ASE in both treatment and control conditions for the same SNP and individual, we can contrast the amount of ASE between the conditions to determine cASE. ASE across cell types was never contrasted because the samples correspond to different individuals. Here we used two approaches to identify cASE (see Fig. 3D): (1) a *qualitative* “on/off” approach using a meta-analysis framework, and (2) a new *quantitative* approach to detect ASE changes even when ASE is present in both conditions.

For the qualitative analysis of cASE, we used meta-analysis of subgroup heterogeneity (MeSH) (Wen and Stephens 2014), a Bayesian meta-analysis approach that has been previously used in eQTL studies to contrast effect sizes across conditions (Maranville et al. 2011) and tissues (Flutre et al. 2013). Here, for each SNP, individual, and treatment/control experiment pair, we assume four different mutually exclusive models: (1) no ASE in either condition, (2) ASE in both conditions, (3) ASE in treatment only, or (4) ASE in control only. Configurations 3 and 4 represent cASE, while configuration 2 represents shared ASE accommodating for random effects in the genetic effect size. This results in a stringent test for cASE.

**Figure 4.**
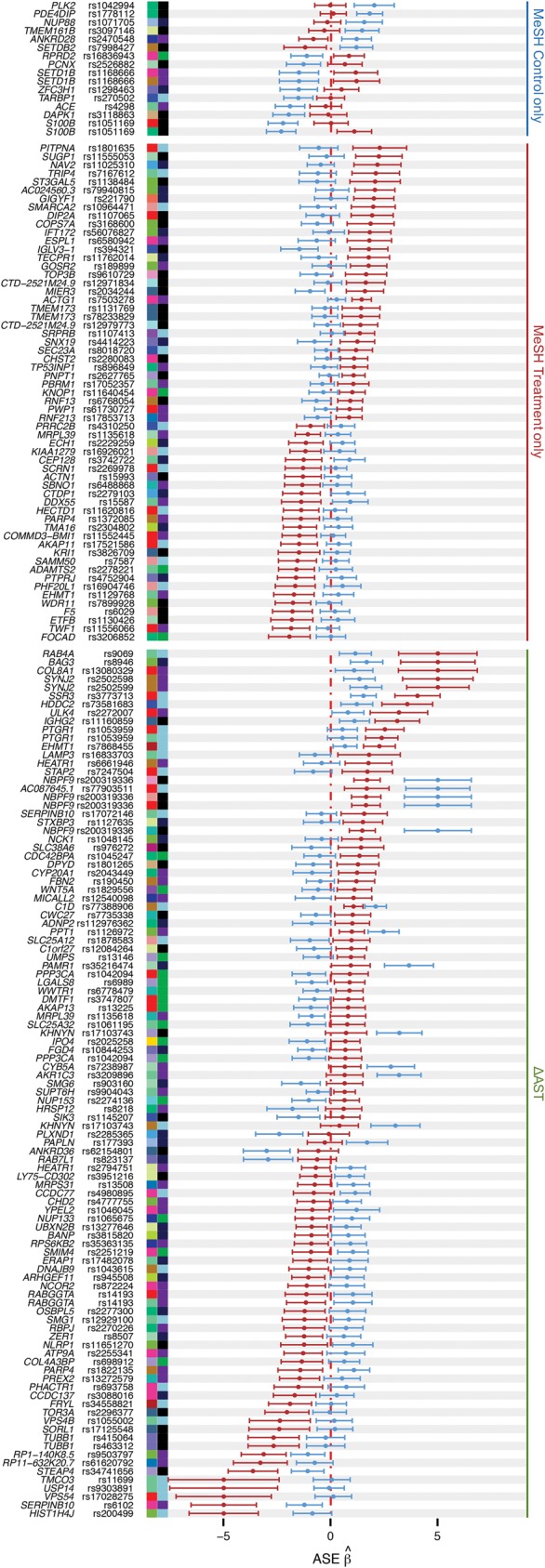
Forest plot of all cASE SNPs. Each row shows the ASE $\hat{\beta }$ for paired treatment (red) and control (blue) conditions. Defined as in Figure 2, colored squares indicate the treatment (*left*) and cell type (*right*) in which cASE was identified, along with the gene name and SNP rsID.

For each of the QuASAR treatment/control measurement pairs, MeSH calculated a Bayes factor (BF) for each configuration. We observed that the majority of genes had ASE shared between the treatment and control conditions (Fig. 3D). We identified 75 SNPs with cASE (difference in the BF_treatment_ or BF_control_ and the BF_shared_ > 30) corresponding to 71 unique genes. We observed a larger proportion of treatment-only cASE compared with control-only cASE (59 vs. 16) (Figs. 3D, 4). These proportions are consistent with observations from eQTL studies contrasting individual treatments and tissues (Maranville et al. 2011; Fairfax et al. 2012; Flutre et al. 2013; Mangravite et al. 2013).

MeSH detects qualitative interactions and strictly requires ASE only in one condition analyzed, either treatment or control, while showing no ASE in the alternate condition. However, these extreme on/off ASE cases are rare. Other cASE models may exist. For example, a prior report identified eQTL with genetic effects in opposite directions in the treatment and control conditions in stimulated monocytes (Fairfax et al. 2014). Additionally, the majority of GxE interactions can arise in cases where the genetic effects differ significantly between treatment and control conditions, but they are nonzero in both conditions.

To capture cASE genes that may have ASE in both conditions but of a significantly different magnitude, we developed an alternate approach named ΔAST (differential allele-specific test). The goal was to quantitatively detect GxE interactions. For each heterozygous site, we compared the QuASAR-derived ASE estimates following treatment to those observed in the matched control for each individual. We calculated a *P*-value for the difference in ASE between the two conditions (Fig. 3E).

A key component of our experimental approach is the inclusion of two sets of vehicle controls in each experimental batch, which empirically estimates the true underlying FDR for identifying cASE, equivalent to permutation-based approaches used in eQTL studies (see Supplemental Methods 10.3). By use of ΔAST, we identified 67 cASE SNPs corresponding to an FDR of 10%, 38 of these cASE SNPs were also identified by MeSH. When we relaxed the FDR threshold (25%), we found a total of 178 cASE SNPs corresponding to 160 genes. Of these genes, 65 were identified with both methods (Figs. 3D–F, 4).

### Features of GxE interactions

**Figure 5.**
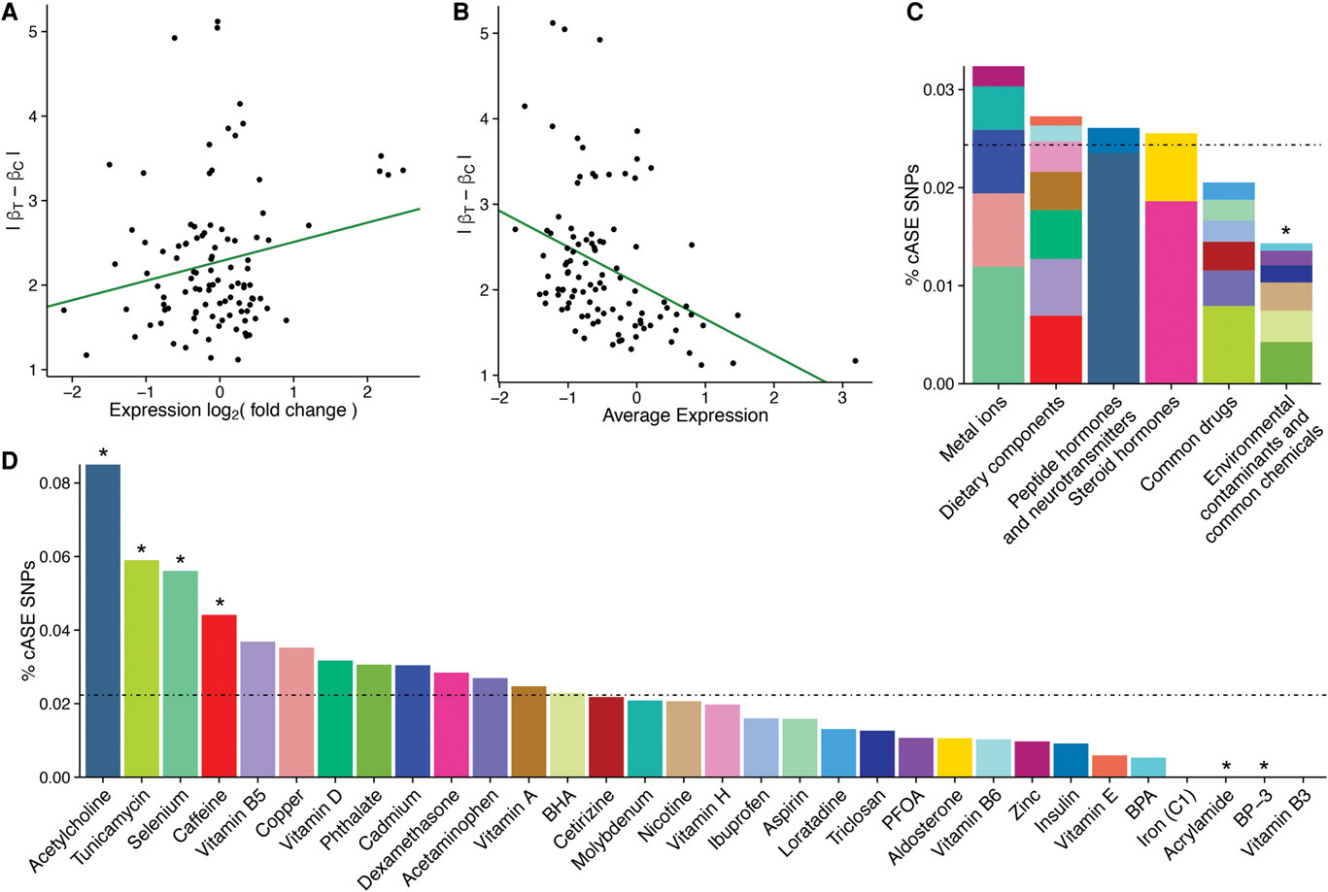
Features of cASE SNPs. (*A*,*B*) Scatter plot comparing the absolute difference in ASE $\hat{\beta }$ between treatment and control (*y*-axis) and the average log_2_ (expression; *A*) or log_2_ (fold change; *B*) between treatment and control samples for cASE SNPs. The green line indicates the trendline from a linear model fit on the points. (*C*,*D*) Percentage of cASE SNPs identified in each treatment category (*C*) or treatment (*D*). For each group, plotted is the percentage of cASE SNPs identified, relative to the number of SNPs tested for that group. The dotted black line represents the average percentage of cASE SNPs across all groups. Groups with an asterisk are significantly enriched or depleted (binomial *P*-value <0.05) relative to the average. The colors in *C* represent the relative proportion of cASE SNPs for each treatment in a treatment category.

When we considered all cASE SNPs, we observed a significant positive correlation between the gene expression log_2_ (fold change) after treatment and differences in the genetic effect in treatment and control samples (Fig. 5A). This result could be a consequence of increased power to detect ASE with more highly expressed genes. However, we observed a negative correlation between gene expression levels and the difference in the genetic effect in the treatment and the control samples (Fig. 5B). This finding suggests that if a gene has sufficiently high expression to test for ASE in both conditions, stronger cASE occurs at genes with stronger positive responses to treatment.

To determine if we can validate some of the few previously known reQTL with cASE, we compiled a list of 134 genes reported to have GxE effects from prior reQTL and ASE studies. Of these genes, 83 were heterozygous and could be tested for cASE in our data (Maranville et al. 2011, 2013; Idaghdour et al. 2012; Franco et al. 2013; Mangravite et al. 2013; Çalişkan et al. 2015; Knowles et al. 2015). Sixty-three out of 83 genes were identified as cASE genes in our analysis (*P* < 0.05) (Supplemental Table S13). For example, gene *IRF5* has a rhinovirus-reQTL (Çalişkan et al. 2015) and showed cASE in response to two treatments: phthalate (*P* = 0.04) and vitamin B5 (*P* = 0.04). Additionally, *IRF5* is also linked to autoimmune responsivity through its association to lupus (Sigurdsson et al. 2005; Graham et al. 2006, 2007). Interestingly, phthalates may play a role in lupus etiology since they induce anti-DNA antibodies (Lim and Ghosh 2005), while vitamin B5 deficiency is found in lupus patients (Leung 2004).

We next wanted to determine the difference in the number of cASE SNPs across cellular environments (Fig. 5C,D; Supplemental Fig. S18). We found that acetylcholine, selenium, and caffeine had significantly higher numbers of cASE SNPs compared with the mean number of cASE SNPs per treatment, while acrylamide and BP-3 had significantly fewer (binomial, *P* < 0.05). In addition, we found that environmental contaminants and common chemicals have a significantly lower proportion of cASE SNPs (*P* < 0.003).

To assess the extent of GxE interactions on gene regulation in different cellular environments, we developed an index that aggregates all cASE tests for each condition and cell type and determines how much the environment can globally perturb ASE. To achieve this, we can compare the correlation of the standardized effect size between treatment and control (as shown in Fig. 3D) across all SNPs tested for a given cell type and condition. We denote this genome-wide measurement as environmental displacement of genetic effect (EDGE) index (for more details, see Methods). Specifically, the EDGE index is the ratio between the pair-wise correlation observed between the two control sets and the correlation observed between the treatment and control conditions. The EDGE index is one for the control conditions (Supplemental Fig. S19A) and will have higher values for treatments that can affect ASE for a large number of genes (Supplemental Fig. S19B). We find significant differences in the EDGE index across many treatments (Supplemental Fig. S20). As expected, we found a high correlation between the proportion of cASE SNPs and the EDGE index (Spearman ρ = 0.717, *P* = 3.8 × 10^−6^) (Supplemental Fig. S19C).

### GxE interactions and complex traits

**Figure 6.**
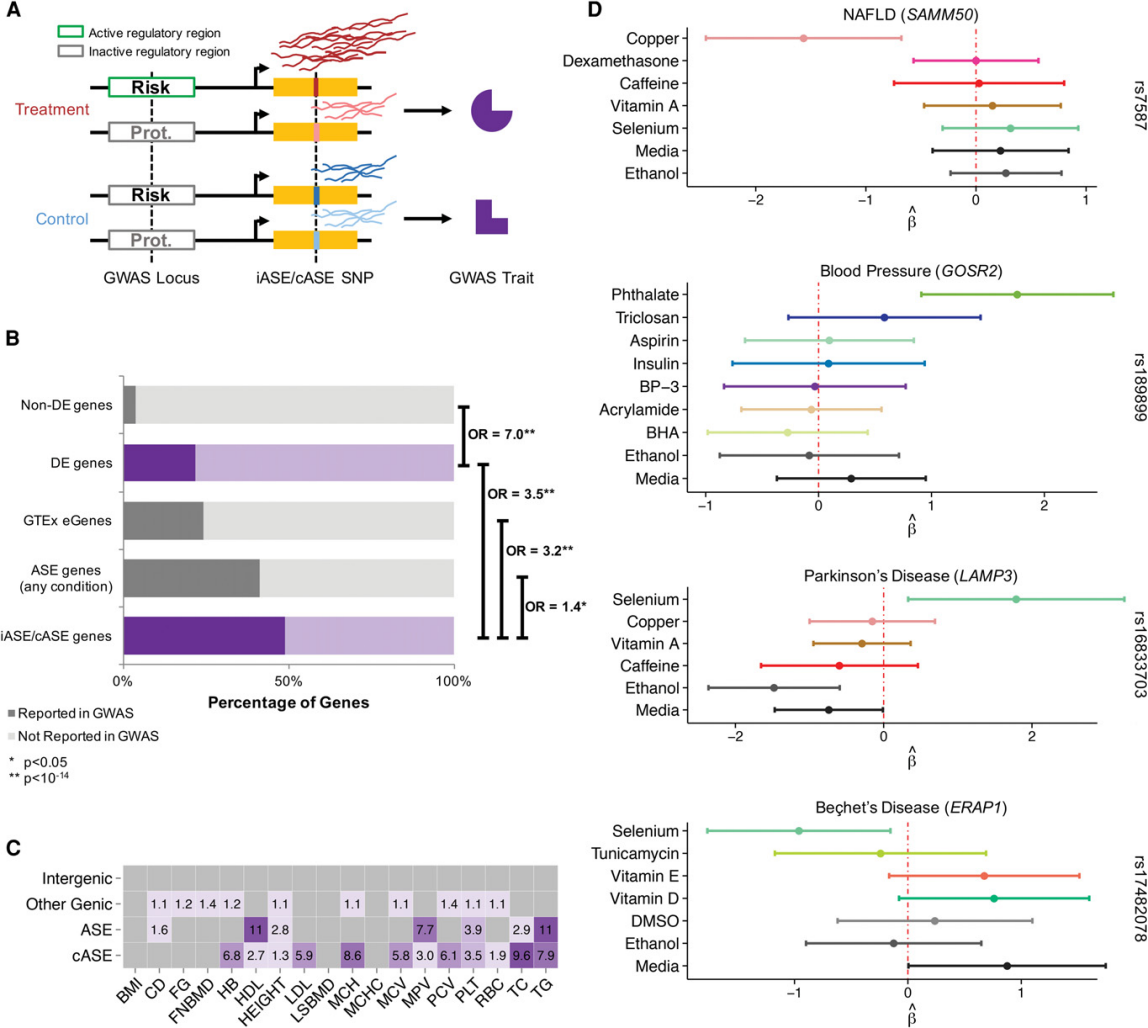
Integration with GWAS. (*A*) Hypothetical model detailing the use of GxE interactions to characterize putative molecular mechanisms for risk or protective environmental factors for complex traits. In the treatment environment, a regulatory region is either active or inactive depending on the haplotype, therefore resulting in different levels of gene expression. In the control environment, the regulatory region is inactive regardless of haplotype. Risk and protective haplotypes are identified in GWAS. (*B*) Enrichment analysis of GWAS genes. Reported genes from the GWAS catalog (version 1.0.1) were compared to different gene sets analyzed in this study: (1) genes that were not differentially expressed in any condition, (2) genes that were differentially expressed in any condition, (3) genes previously associated with an eQTL in GTEx (eGenes) (The GTEx Consortium 2015), (4) genes containing ASE in any condition, and (5) genes containing either iASE or cASE. The percentage of genes in these data sets that were found in the GWAS catalog is indicated by a darker shade. Genes that can be perturbed by our environments are highlighted in purple and indicate a GxE mechanism for the GWAS association. Odds ratios and enrichment *P*-values were calculated using a Fisher's exact test and are shown on the *right* for each pair of gene categories contrasted. (*C*) Genome-wide efficient mixed model association (GEMMA) per SNP heritability estimates relative to the genomic average for cASE (SNPs in genic regions with cASE or iASE), ASE (SNPs in genic regions with ASE), other genic (SNPs in genic regions), and intergenic (SNPs <100 kb from any gene). Only significant enrichment values are reported, with a darker tone of purple indicating a higher enrichment odds ratio relative to the genome average. (*D*) Forest plots of four cASE SNPs in genes associated with a GWAS trait. For each SNP, shown is the ASE $\hat{\beta }$ for each treatment in which the SNP was tested. The 95% CI bars are colored for each treatment as in Figure 2.

We then used the GxE interactions we identified in vitro to characterize putative molecular mechanisms for risk or protective environmental factors for complex traits (Fig. 6A). We found that 22% of DE genes overlap with those identified in GWAS analyses (Welter et al. 2014) compared with 4% of nondifferentially expressed genes expressed in our samples (Fig. 6B). This overlap corresponds to a sevenfold enrichment (*P* < 2.2 × 10^−16^). These results suggest that genes responsive to our treatments are more likely involved in organismal traits.

To investigate the role of GxE interactions in complex traits directly, we analyzed genes containing iASE or cASE. Forty-nine percent of genes (105 out of 215) that contain either iASE or cASE were identified by GWAS as associated with various complex traits; this corresponds to a 3.5-fold enrichment (*P* < 2.2 × 10^−16^) compared with DE genes without iASE or cASE. Importantly, genes with iASE or cASE also have a 1.4-fold increased relevance for complex traits (*P* = 0.025) compared with ASE genes and a 3.2-fold enrichment (*P* < 4.3 × 10^−15^) compared with genes with eQTL identified at baseline (eGenes from GTEx). Note that by design much of our detected ASE may have an environmental component, but we may lack the power to claim cASE/iASE. These results suggest that GxE interactions represent an important mechanism for inter-individual variation in complex traits.

We find similar results when we analyze per SNP heritability for 18 complex traits using genome-wide efficient mixed model association (GEMMA) (Zhou and Stephens 2012; Zhou 2016). Similar to the LD-score regression method that partitions heritability estimates across SNPs functional categories (Gusev et al. 2014), we contrasted SNPs in genes with cASE/iASE, genes with ASE, genes without ASE, and inter-genic regions. The per SNP heritability for each of these categories is then compared with the genome average. A higher value of per SNP heritability for one of these categories indicates a higher number of causal SNPs, higher effect sizes, or both in that category. We found that the per SNP heritability estimate for SNPs in genes with ASE is 11.1 times higher than the genome average for high-density lipoprotein (HDL). For 13 of the 18 traits analyzed, per SNP heritability estimates for SNPs in genes with cASE, iASE, or ASE were significantly higher than the genome average. For seven of these, the cASE and iASE category estimates were even higher than any other partition (Fig. 6C), thus indicating that GxE interaction for these traits are particularly relevant. The highest values for cASE and iASE were observed for blood total cholesterol level (TC; 9.7-fold), triglicerides (TG; 7.9-fold), and mean corpuscular hemoglobin levels (MCH; 8.6-fold). Overall, these results suggest an important role for GxE interaction in a large number of traits.

When we isolated genes with iASE, we found 28 genes associated with a phenotype in the GWAS catalog (Supplemental Table S18). Additional investigation into these genes may yield insights not only on the GxE role in specific traits but also on the underlying molecular mechanisms. For example, previous reports show that caffeine prevents and treats obesity presumably through mitotic clonal expansion effects (Li et al. 2015; Kim et al. 2016; Ohara et al. 2016). Our work suggests that caffeine activates the GIPR pathway, which regulates insulin production. *GIPR* is linked to obesity and several obesity-related traits, including body mass index and type 2 diabetes (Saxena et al. 2010; Speliotes et al. 2010; Fox et al. 2012; Okada et al. 2012; Wen et al. 2012, 2014; Berndt et al. 2013; Mahajan et al. 2014). We identified a SNP, rs5390, in *GIPR* that demonstrates iASE following caffeine treatment. Specifically, we found higher *GIPR* expression and ASE favoring the rs5390 reference allele following caffeine treatment and low expression in controls. The rs5390 reference allele is located on the same haplotype as the nonrisk allele for body mass index in the individual sample used here (Wen et al. 2012, 2014; Okada et al. 2012). These results suggest that caffeine may reduce obesity through its effect on gene expression and ASE in *GIPR*.

Among the genes with cASE, 79 are associated with complex traits in the GWAS catalog (Supplemental Table S18). Figure 6D shows four examples of cASE genes associated with complex traits. These include cASE in *SAMM50* in response to copper, in *ERAP1* in response to selenium treatment, in *GOSR2* following treatment with mono-n-butyl phthalate, and in *LAMP3* in response to selenium. This last example may explain the influence of selenium on Parkinson's disease (PD). Previous studies found reduced selenium levels in PD patients (Shahar et al. 2010). In addition, selenium reduces bradykinesia, a well-described symptom of PD, in rats (Ellwanger et al. 2015), suggesting that higher selenium levels would be beneficial for PD patients. A GWAS hit for PD (Do et al. 2011; International Parkinson Disease Genomics Consortium 2011) was an eQTL for *LAMP3*, where the reference/PD-risk allele led to increased expression of *LAMP3* (The GTEx Consortium 2015). In our data, cASE at rs16833703 in *LAMP3* preferentially expressed the alternative allele at this SNP, located on the same haplotype as the nonrisk allele at the GWAS SNP. These data suggest that selenium is beneficial for PD patients through its influence on allelic expression in *LAMP3*. Overall, these cASE examples illustrate genes associated with complex traits, with a plausible biological association with the treatment (for details on the other three genes, see the Supplemental Material).

## Discussion

We presented a scalable high-throughput approach to characterize the effect of environmental and genetic perturbation on gene expression levels. In this study, we tightly controlled environmental exposure using in vitro treatments in different cell types and analyzed the transcriptional response for hundreds of conditions that were previously uncharacterized. These results will be highly valuable to many researchers interested in changes to specific genes or pathways following various treatments and cell types. Among the DE genes, we found that 22% have been associated with complex traits in GWAS. These results strongly suggest a key environmental component for many complex traits and should assist the design of future studies. For example, this resource can help in selecting relevant environmental variables that should be considered in animal models for human complex traits, in patient studies, and in reanalyzing GWAS data when the relevant exposure variable was collected as part of the study.

One of the main advantages of our approach is that it can be used to detect GxE interaction in a single individual for many treatments and cell types using ASE analysis. Compared to model organisms and transgenic models, studying GxE interaction in humans poses significant challenges. In clinical trials, a limited number of exposures can be tested, while in large-scale epidemiological studies many exposures convolve together. In this study, we have analyzed three individuals per cell type in order to explore a large number of environmental conditions that would not be practical for a reQTL study design. While some GxE interactions may be detected in a conventional baseline eQTL study, this would only occur if all or a subset of the samples was exposed to a relevant environment. However, in eQTL studies, the specific exposure would likely remain uncharacterized as a latent variable that may be unknown or difficult to model. Though we do not require many individuals, our approach is limited by the requirement of having two heterozygous variants: at the causal regulatory variant and at the variant for which ASE is measured. A small fraction of the ASE we detected may actually correspond to low-frequency variants that are sampled in three individuals, but the majority will correspond to common variants. The requirement that at least one of the three individuals is a double heterozygote means that we are missing instances of GxE interaction, especially those at low allele frequencies. Nonetheless, the 215 instances of GxE described here represent a lower bound to the amount of GxE signal that can be identified by applying our approach to additional treatment panels, cell types, and/or larger sample sizes.

Our catalog of GxE interactions, and future ones expanding on the one generated here, will be a necessary resource to thoroughly annotate genes and create a bridge between epidemiological and genome-wide association studies. Here we showed that 49% of genes with GxE interactions are GWAS genes. Although limited by our false-negative rate, we compiled the most comprehensive list to date of GxE and relevant environmental conditions that can aide in the interpretation of specific GWAS findings. We provided some examples of candidate GxE mechanisms for complex traits and released our results as a browsable web-resource. Mining of our results by other researchers has the potential to inform new GWAS findings and identify latent variables in GWAS that are important risk/protective factors for human complex traits and diseases.

In future research, we anticipate that the approach we developed will potentially aid in precision medicine to tailor medication exposures using patient cells for improved patient outcomes. Indeed, when considering potential translation of these findings to clinical practice, in vitro measurements of ASE for a large panel of cell types, extracted and derived from single-patient stem cells, are a promising solution to studying rare disease variants and individualized outcomes of combinatorial interactions of common genetic variants.

## Methods

### Cell culture and treatments

Experiments were conducted using the following cell types: lymphoblastoid cell lines (LCLs), PBMCs, human umbilical vein endothelial cells (HUVECs), human SMCs, and melanocytes. LCLs (GM18507, GM18508, and GM19239) were purchased from Coriell Cell Repository, cultured, and treated as described previously (Moyerbrailean et al. 2015). PBMCs were derived from whole human blood purchased from Research Blood Components. Blood specimens were obtained from three individual donors. Primary HUVECs and SMCs were isolated from human umbilical cord tissue collected shortly following birth. Additionally, cryopreserved HUVECs (CC-2517-0000315288) and SMCs (CC-2579-7F3794) were purchased from Lonza. For additional details on HUVEC and SMC preparation, see Supplemental Methods 1. Primary melanocytes (NHEM) isolated from neonatal foreskin were purchased from Lonza (CC-2504 lot no. 252410 and 5F0885J) and from Promocell (C-12400 lot no. 3052103.1). Details on cell culturing are provided in the Supplemental Methods 2. Supplemental Table S1 shows the concentrations used for each treatment. For each treatment panel and cell type, cells derived from three individuals were treated at the same time on a 96-well plate. A schematic of the study design is provided in Supplemental Figure S1.

### RNA-seq library preparation and sequencing

We used a two-step approach to gene expression analysis that we recently developed (Moyerbrailean et al. 2015). A 96-library pooling and shallow sequencing strategy (<10 M reads per library) (Supplemental Table S2) were used to minimize the amount of resources used in the first step. For the second step, we repooled a selection of the initial libraries (Fig. 1B; Supplemental Methods 8.1) to achieve a more uniform allocation of sequencing reads across samples (130 M reads/sample on average) (Supplemental Table S4). Pools of 96 samples from step 1 were sequenced on two lanes of an Illumina HiSeq2500 in fast mode to obtain 50-bp paired-end reads at the University of Chicago and at the Michigan State University Genomics Cores or were sequenced on one lane of the Illumina NextSeq500 for 75 cycles of paired-end in HO mode in the Luca/Pique-Regi laboratory. Step 2 resequencing was performed on the NextSeq500 in the Luca/Pique-Regi laboratory. The number of reads collected for each sample in step 1 and step 2 is reported in Supplemental Tables S2 and S4, respectively.

### Sequence alignment and post-processing

RNA-seq data for step 1 was processed as described previously (Moyerbrailean 2015). For step 2, reads were aligned to the hg19 human reference genome using STAR (https://github.com/alexdobin/STAR/releases, version STAR_2.4.0h1) (Dobin et al. 2013) and the Ensembl reference transcriptome (version 75). Details are provided in Supplemental Methods 7.1. We did not realign the reads to GRCh38 because hg19 is the version of the reference human genome used in the release of the 1000 Genomes Project that we used for pileup. The 1000 Genomes Project data were not available in GRCh38 coordinates until October 26, 2016. Realigning the reads should not affect the conclusions as any problematic region of the genome is excluded from any analysis as detailed in the Supplemental Material. To correct for potential alignment biases, we used the WASP suite of tools for allele-specific read mapping (https://github.com/bmvdgeijn/WASP, downloaded 09/15/15) (Van de Geijn et al. 2015). Note that we do not use the WASP combined haplotype test (CHT) as we tested each SNP separately using QuASAR (Harvey et al. 2015). Retained read counts per sample after filtering can be found in Supplemental Table S4.

### Differential gene expression

To identify DE genes, we used the method implemented in the software DESeq2 (R version 3.2.1, DESeq2 version 1.8.1) (Love et al. 2014). DE genes were determined as genes with at least one transcript having a Benjamini-Hochberg controlled FDR (BH-FDR) (Benjamini and Hochberg 1995) of 10% and an absolute log_2_ (fold-change) >0.25. The same procedure was used for step 1 and step 2. A summary of differential expression for both steps can be found in Supplemental Tables S3 and S5, and a full set of differential expression results from step 2 can be found in Supplemental Table S6.

### Network analysis with WGCNA

For network analysis, we used gene expression data normalized as described in Supplemental Methods 8.4. We combined all the data across cell types, treatments, and individuals, resulting in a matrix with 14,527 rows (genes) and 297 columns (samples). We then used WGCNA (Langfelder and Horvath 2008), version 1.47, implemented in R to build an unsigned network. A soft thresholding power of six was chosen, and the network was built using the automated block-wise modules pipeline using Pearson correlations, a signed topological overlap matrix, and a minimum module size of 10. Modules were cut from the network dendrogram with the dynamic hybrid tree cut method, and the module eigengene was calculated as the first principal component of each module's expression matrix. A measure of module membership was calculated for each gene by correlating the gene's expression profile with its module's eigengene. More details on the network module analysis are in Supplemental Methods 8.6.

### Joint genotyping and ASE inference

To create a core set of SNPs for ASE analysis, we started with all the SNPs from the phase 3 release of the 1000 Genomes Project Consortium (www.1000Genomes.org, v5b.20130502, downloaded on 08/24/15) (The 1000 Genomes Project Consortium 2015) and first removed SNPs with low minor allele frequency (MAF <5%). We also removed SNPs within the regions of annotated copy number variation and ENCODE blacklisted regions (Degner et al. 2012), leaving a total of 7,340,521 SNPs in the core set. Counts of reads covering each allele at selected SNPs (Supplemental Methods 9.1) were obtained by “piling up” aligned reads for each sample over SNPs using samtools mpileup (Li et al. 2009) and the hg19 human reference genome. All sample pileups for a given individual across all treatment conditions and the two treatment plates were processed together (not merged) using the QuASAR package (Harvey et al. 2015) for joint genotyping. ASE inference was performed for each sample separately. Heterozygous SNPs with a read coverage greater than 40 were tested for ASE using QuASAR (Harvey et al. 2015). A summary of the amount of ASE detected in each sample is in Supplemental Figure S10 and Supplemental Table S9. A full list of SNPs tested can be found in Supplemental Table S10.

### Identification of induced ASE

To identify genes with iASE, we selected SNPs that were well covered in the treatment (i.e., more than 40 reads) and had ASE (10% FDR) but had little to no expression in the matched control. We used a coverage threshold in the control of 10 × (*D*_*C*_/*D*_*T*_), where *D*_*C*_ and *D*_*T*_ represent the sequencing depth of the control and treatment libraries, respectively, in TPM (see Supplemental Methods 9.3). This equates to a ratio of 40 reads to 10 (expression in the control is fourfold lower than the minimum required for a gene to be considered expressed in the ASE analysis) while accounting for sequencing depth differences. Finally, we required the SNP-based log_2_ (fold change) (Supplemental Methods 9.3) to be >log_2_(5).

### Meta-analysis of subgroup heterogeneity

We used MeSH to model potentially heterogeneous cASE effects across multiple subgroups contained within the data. The input to MeSH is a pair of ASE observations derived from QuASAR summarized by the parameter β measuring the allelic imbalance and a standard error of the parameter. To look specifically at conditional ASE, a BF for cASE is calculated as BF_treatment_ − BF_shared_ (treatment-only cASE) and BF_control_ − BF_shared_ (control-only cASE). All the cASE BFs are then used to rank and select the observations with strongest evidence for cASE.

### ΔAST: a novel method to measure cASE

Differential *Z*-scores (*Z*_Δ_) were calculated from QuASAR β parameters using the following formula. For each SNP,

(1) $$Z_{\Delta }=\frac{\beta_{T}-\beta_{C}}{\sqrt{se_{T}^{2}+se_{C}^{2}}},$$

where β_*T*_ and *se*_*T*_ represent the estimates for the ASE parameter and its standard error for the treatment condition, and β_*C*_ and *se*_*C*_ represent the corresponding estimates for the control condition. The *Z*_Δ_ scores were then normalized by the standard deviation across *Z*_Δ_ scores corresponding to control versus control (CO1 vs. CO2). Finally, *P*-values (*P*_Δ_) are calculated from the *Z*_Δ_ scores as *P*_Δ_ = 2 × pnorm(−|*z*|). Under the null, *Z*_Δ_ are asymptotically normally distributed, and Figure 3C shows that when contrasting the two sets of controls the *P*_Δ_-values are almost uniformly distributed as expected. To further correct for this small deviation, we used the control versus control *P*-values to empirically estimate the FDR (see Supplemental Methods 10.3). The list of significant cASE SNPs is in Supplemental Table S11.

### Analysis of EDGE

Within each treatment and cell line subgroup, we examined the Pearson's correlation of the treatment standardized effect size (ASE *Z*_*T*_ = β_T_/*se*_*T*_) to the matched control one (ASE *Z*_*C*_ = β_C_/*se*_*C*_) across all SNPs tested (see Supplemental Fig. S19). This correlation measures the consistency of the genetic effect between the treatment and control, and therefore, a lower correlation indicates a higher perturbation or displacement of the genetic effects. We define this as the EDGE index, which is formally defined as

(2) $${\mathrm{EDGE}}_{s,t}=\frac{\mathrm{Pearson}(Z_{s,CO1},Z_{s,CO2})}{\mathrm{Pearson}(Z_{s,t},Z_{s,c})},$$

where Pearson(*Z*_*s,t*_, *Z*_*s,c*_) is the sample Pearson correlation coefficient between treatment *t* and control *c* ASE *Z*-scores across all observed SNPs in cell type *s*. Equivalently, Pearson(*Z*_*s,CO1*_, *Z*_*s,CO2*_) is the Pearson correlation coefficient between the two control sets ASE *Z*-scores across all observed SNPs in cell type *s*. The EDGE index values for each cell type and condition can be found in Supplemental Figure S20 and Supplemental Table S14.

### Analysis of heritability enrichment using GEMMA

To run GEMMA (Zhou and Stephens 2012; Zhou 2016), we partitioned SNPs genome wide to create a category file. Each SNP was assigned to one of the following categories: cASE (genic regions with conditional ASE) or iASE (genic regions with induced ASE), ASE (genic regions with ASE), other genic (genic regions that do not fall into any of the categories above), and intergenic (>100 kb from any gene). We then used GEMMA to compute the SNP correlations among different categories from a reference panel (502 individuals of European ancestry from the 1000 Genomes Project). This was followed by summing the *Z*^2^ statistics from the GWAS meta-analysis within the categories. Finally, we computed the proportion of variance and the fold enrichment of heritability explained by each category. A table of the results can be found in Supplemental Table S17.

## Data access

The RNA sequencing data from this study have been submitted to the database of Genotypes and Phenotypes (dbGaP, https://www.ncbi.nlm.nih.gov/gap) under the accession number phs001176.v1.p1. Additional browsing tools for exploring the data in this project are available at http://genome.grid.wayne.edu/gxebrowser.

## Supplementary Material

Supplemental Material
