# Supplementary material for: High-throughput allele-specific expression across 250 environmental conditions
Source: Genome Res. 2016 Dec;26(12):1627–38. doi: 10.1101/gr.209759.116 (PMC5131815; doi:10.1101/gr.209759.116)
Supplement: Supplemental Material [file supp_26_12_1627__index.html]

High-throughput allele-specific expression across 250 environmental conditions — Supplemental Material 

# High-throughput allele-specific expression across 250 environmental conditions

## Supplemental Material

- Supplemental\_Table\_S2.xlsx
- Supplemental\_Table\_S3.xlsx
- Supplemental\_Table\_S4.xlsx
- Supplemental\_Table\_S5.xlsx
- Supplemental\_Table\_S7.xlsx
- Supplemental\_Table\_S8.xlsx
- Supplemental\_Table\_S10.txt.gz
- Supplemental\_Table\_S12.txt.gz
- Supplemental\_Table\_S18.xlsx
- Supplemental\_Materials.pdf
- Supplemental\_Table\_S6.tar.gz
