# Supplementary material for: High-throughput allele-specific expression across 250 environmental conditions
Source: Genome Res. 2016 Dec;26(12):1627–38. doi: 10.1101/gr.209759.116 (PMC5131815; doi:10.1101/gr.209759.116)
Supplement: Supplemental Material [file supp_gr.209759.116_Supplemental_Materials.pdf]

Supplemental text

High-throughput allele-specific expression across 250 environmental conditions

G. A. Moyerbrailean[1,†], A. L. Richards[1,†], D. Kurtz[1,†], C. A. Kalita[1,†], G. O. Davis[1], C. T. Harvey[1], A. Alazizi[1], D. Watza[1], Y. Sorokin[3], N. Hauff[3], X. Zhou[2], X. Wen[2], R. Pique-Regi[1,3,*] F. Luca[1,3,*],

[1]Center for Molecular Medicine and Genetics, Wayne State University

[2]Department of Biostatistics, University of Michigan

[3]Department of Obstetrics and Gynecology, Wayne State University

[†]These authors equally contributed to this work

[*]To whom correspondence should be addressed: fluca@wayne.edu, rpique@wayne.edu

1 Cells

Experiments were conducted using the following cell types: lymphoblastoid cell lines (LCLs), peripheral blood mononuclear cells (PBMCs), human umbilical vein endothelial cells (HUVECs), human smooth muscle cells (SMCs) and melanocytes. LCLs (GM18507, GM18508, and GM19239) were purchased from Coriell Cell Repository, cultured and treated according to (*Moyerbrailean et al., 2015*).

PBMCs were derived from whole human blood purchased from Research Blood Components. Blood specimens were obtained from 3 individual donors. PBMCs were isolated by density gradient centrifugation, using a Ficoll-Paque isolation protocol. For isolation, PBS-diluted blood was gently layered over room temperature Histopaque-1077 (Life Technologies), centrifuged at 400 xg and the mononuclear cell layer was collected using a transfer pipette. Following isolation, PBMCs were resuspended in RPMI 1640, supplemented with 10% charcoal stripped FBS (CS-FBS) and 0.1% Gentamycin, at a 1×10^6 cells/mL and stored overnight at 4°C, for use the following day. Immediately before treatment, PBMCs were activated with PHA (2.5μg/mL).

Primary HUVECs and SMC were isolated from human umbilical cord tissue collected shortly following birth. Umbilical cord tissue specimens were obtained from healthy full-term pregnant women, admitted to DMC Hutzel Women's Hospital (Detroit, Michigan). Two cord specimens, between 10 and 30 cm in length, were first rinsed with warm PBS and a blunt-ended needle was inserted into the umbilical vein at one end of the cord, and subsequently clamped in place. The cord was then purged to remove any excess blood from the vein. The other end of the cord was then sealed, in a manner identical to the first end, and pre-warmed 0.25% trypsin-EDTA (Gibco) was then injected into the vein. Following a 20 minutes incubation, at 37°C, detached HUVECs were rinsed from the vein, collected by centrifugation, counted, and seeded into an appropriate vessel at 10,000 cells/cm^2, in EGM-2 growth medium (Lonza). Expanded cultures were cryopreserved prior to be used in the experiments. SMCs were collected from two additional cords. Briefly, following HUVECs isolation, the vein was purged with PBS and pre-warmed Collagenase A/Dispase II solution was slowly injected in the vein until it becomes moderately distended. The filled cord was then incubated in pre-warmed PBS for 60-120 min at 37°C, in the water bath. Following incubation, SMCs were collected from the vein with warm PBS, and resuspended in EGM-2 growth medium. Cells were then collected by centrifugation, counted, and seeded into an appropriate vessel at 3500 cells/cm^2 in SmGM-2 medium (Lonza#: CC-4149) and cultured at 37°C and 5% CO_2 prior to cryopreservation. All specimens for this study were collected following guidelines approved by the institutional

review board (#013213MP4E) of Wayne State University. Additionally, cryopreserved HUVECs (CC-2517-0000315288) and SMCs (CC-2579-7F3794) were purchased from Lonza.

Primary melanocytes (NHEM) isolated from neonatal foreskin were purchased from Lonza (CC-2504 lot # 252410 and 5F0885J) and from Promocell (C-12400 lot # 3052103.1).

2 Cell culturing prior to the treatments

LCLs were cultured prior to treatment as described in (*Moyerbrailean et al., 2015*). PBMCs were seeded in phenol-red free RPMI 1640, supplemented with 10% CS-FBS and 0.1% Gentamycin at $1x10^6$ cells/mL on a 96-well plate on the day of the treatment. For all HUVEC lines, cells were seeded onto 96-well plates, at 5000 $cells/cm^2$, in EGM-2. Following a 24 hour recovery period, the medium was changed to a "starvation medium", composed of phenol-red free EGM-2, without Hydrocortisone and Vitamin C and supplemented with 2% CS-FBS. Cell starvation was continued for 48 hour prior to treatment. HUASMCs and HUVSMCs (both referred as SMCs) were seeded at10,000 $cells/cm^2$ in complete SmGM-2 medium on a 96-well plate. After 24hrs they were cultured in SmGM-2-starvation medium, containing CS-FBS and without insulin for 2 days. NHEMs were seeded at 10,000 $cells/cm^2$ in complete MGM-4 (Lonza#: CC-4435) medium on a 96-well plate. Following a recovery period of 24 hrs, the medium was changed to MGM-4 starvation medium (with CS-FBS, without insulin and hydrocortisone) for 2 days.

3 Treatments

Table S1 shows the concentrations used for the each treatment. These were derived from the Clinical Guidelines Mayo Clinic Reference Levels (`http://www.mayomedicallaboratories.com`) and the CDC National Biomonitoring Report Reference Levels (`http://www.cdc.gov/biomonitoring/`). Vehicle controls were included to represent the solvent used to prepare the different treatments. Treatments were subdivided into two panels, each containing the appropriate controls. For each treatment panel and cell type, cells derived from three individuals were treated at the same time on a 96-well plate. A schematic of the study design is provided in Figure S1. On each plate, the control treatments were performed in triplicate. For all cell types except PBMCs (cells grown in triplicates), cells were treated in duplicate (two plates per treatment panel) and the two duplicates for each treatment sample were pooled prior to RNA isolation, to ensure that enough RNA could

be obtained. Because we were interested in early changes in the transcriptome, cells were treated for six hours (no cell doublings).

4 RNA-seq library preparation

Treated cells were collected by centrifugation at 2000 rpm and washed 2x using ice cold PBS. Collected pellets were lysed on the plate, using Lysis/Binding Buffer (Ambion), and frozen at -80°. Poly-adenylated mRNAs were subsequently isolated from thawed lysates using the Dynabeads mRNA Direct Kit (Ambion) and following the manufacturer instructions. RNA-seq libraries were prepared using a protocol modified from the NEBNext Ultradirectional (NEB) library preparation protocol to use 96 Barcodes from BIOOScientific added by ligation, as described in (*Moyerbrailean et al., 2015*). The individual libraries were quantified using the KAPA real-time PCR system, following the manufacturer instructions and using a custom-made series of standards obtained from serial dilutions of the phi-X DNA (Illumina).

5 Two step high-throughput screening approach

We used a two step approach to gene expression analysis that we recently developed (*Moyerbrailean et al., 2015*). Briefly, in the first step all samples were experimentally processed in parallel, from tissue culture and treatments to library preparation, thus minimizing experimental variation from testing dozens of conditions at the same time (Figure S1). Additionally, high multiplexing allows for the reduction of the number of controls that would need to be repeated across different treatment batches in a less multiplexed experimental setup (here 32 treatments plus 3 controls for each of three individuals represented on a plate, see Figure S1). A 96-library pooling and shallow sequencing strategy ($<$10M reads per library, Table S2) was then used to minimize the amount of resources used in the screening step. This allowed for a rapid screen of a large number of treatment conditions, while sequencing resources could be strategically allocated to the in depth analysis of relevant cases.

For the second step, we repooled a selection of the initial libraries (Section 8.1, Figure 1B), without additional experimental costs. Furthermore, using a two-step approach allowed us to repool the samples to achieve a more uniform allocation of sequencing reads across samples (130M reads/sample on average, Table S4).

6 Sequencing

A flowchart of the 2-step sequencing procedure can be found in Figure S2. Pools of 96 samples from Step 1 were sequenced on two lanes of an Illumina HiSeq2500 in fast mode to obtain 50bp PE reads, at the University of Chicago and at the Michigan State University Genomics Cores; or on one lane of the Illumina NextSeq500 for 75 cycles PE in HO mode in the Luca/Pique-Regi laboratory. To prepare subpools for deep sequencing (Step 2), we used the re-pooling approach described in (*Moyerbrailean et al., 2015*) which allows for iterative adjustments of pooling proportions in order to reach the desired total number of reads through multiple re-pooling and sequencing runs. Step 2 resequencing was performed on the NextSeq500 in the Luca/Pique-Regi laboratory. The number of reads collected for each sample in step 1 and step 2 is reported in Table S2 and Table S4, respectively. We collected 6.6 billion reads in step 1 (averaging 8.2 million reads per sample) and 33.5 billion reads of deep sequencing data in step 2 (averaging 113 million reads per sample). Note that reads from shallow sequencing (obtained during the first step) were not combined to the ones from deep sequencing (second step) because of differences in read length.

7 Pre-processing of RNA-seq data

7.1 Sequence alignment

RNA-seq data for step 1 was processed as described in (*Moyerbrailean et al., 2015*). Briefly, raw sequencing reads were aligned to the hg19 human reference genome using `bwa mem` (*Li and Durbin, 2009*)(`http://bio-bwa.sourceforge.net`). Reads with quality <10 and duplicate reads were removed using `samtools rmdup` (`http://github.com/samtools/`). Read counts per sample after filtering steps can be found in Table S2.

For step 2, reads were aligned to the hg19 human reference genome using STAR (*Dobin et al., 2013*)(`https://github.com/alexdobin/STAR/releases`, version STAR_2.4.0h1), which is a highly efficient aligner that can handle split reads, and the Ensembl reference transcriptome (version 75). To index the genome using the reference transcriptome, we used the following command:

```
STAR --runThreadN 12 --runMode genomeGenerate --genomeDir ./ \
          --genomeFastaFiles hg19.fa --sjdbGTFfile ensembl75.gtf \
          --sjdbOverhang 150
```

Alignment of the fastq files was then carried out using the following options:

```
STAR --runThreadN 12 --genomeDir <genome> \
          --readFilesIn <fastqs.gz> --readFilesCommand zcat \
          --outFileNamePrefix <stem> --outSAMtype BAM Unsorted \
          --genomeLoad LoadAndKeep
```

where *<genome>* represents the location of the genome and index files, *<fastqs.gz>* represents that sample's fastq files, and *<stem>* represents the filename stem of that sample. For each sample (individual cell line and treatment), merging of sequencing replicates (lanes and runs on the same sequencer) and quality filtering was performed using `samtools` (version 2.25.0), using a quality score cutoff of "10", which corresponds to uniquely mapping reads. Note that different individuals are never merged, and that independent library preparations (i.e., controls on separate plates) are never merged.

7.2 Sequence filtering

To correct for potential alignment biases, we used the WASP suite of tools for allele-specific read mapping (*Van de Geijn et al., 2015*)(`https://github.com/bmvdgeijn/WASP`, downloaded 09/15/15). Note that we do not use the WASP combined haplotype test (CHT) as we tested each SNP separately using QuASAR (*Harvey et al., 2015*). The objective of the WASP pre-processing is to keep reads that are not ambiguously mapping to multiple places while considering all the known allelic variants contained in the corresponding genomic location. Briefly, after a first step of alignment and quality filter, we used `find_intersecting_snps.py` to identify reads overlapping SNPs of interest (see Section 9.1 for details on which SNPs are used). To do this, we used the paired-end flag and the default search window of 100,000 base pairs. Output fastqs generated by WASP, which were modified to include reads matching reference and alternate bases when overlapping SNPs, were then re-aligned using STAR as detailed before (see Section 7.1). To recover reads that mapped correctly (e.g., reads with reference and alternate allele mapping to same position), we used `filter_remapped_reads.py` with the paired-end flag. Finally, reads that did not overlap SNPs were merged with the retained reads after filtering, and duplicates were removed using WASP's `rmdup.py`. Importantly, when we identified duplicate paired reads with the reference and alternate allele, we randomly retained only one copy. Retained read counts per sample after filtering can be found in Table S4.

8 Gene expression analysis

8.1 Differential gene expression

To identify differentially expressed (DE) genes, we used the method implemented in the software DESeq2 (*Love et al., 2014*) (R version 3.2.1, DESeq2 version 1.8.1). DESeq2 estimates variance-mean dependence in the read counts for each gene and tests for differential expression based on a model using the negative binomial distribution. Transcripts with fewer than 20 reads total on a given plate were discarded. To better estimate the dispersion parameters, the DESeq2 model was fit on all sequencing data from a single plate simultaneously (i.e., all treatment samples, and without merging the technical replicates of the control samples, see Figure S1):

$$K_{ij} \sim \mathrm{NB}(\mu_{ij}, \alpha_{ij}) \tag{1}$$

$$\mu_{ij} = s_j q_{ij} \tag{2}$$

$$\log_2(q_{ij}) = \beta_{i,0} + \beta_{i,\mathrm{Cl}(j)} + \beta_{i,\mathrm{Tr}(j)} \tag{3}$$

where, for each transcript i and sample j, the read counts K_{ij} are modeled using a negative binomial distribution with fitted mean μ_{ij} and a gene-specific dispersion parameter α_i. The fitted mean is composed of a sample-specific size factor s_j and a parameter q_{ij} proportional to the expected true concentration of fragments for sample j. The coefficient β_0 represents the mean effect intercept, $\beta_{\mathrm{Cl}(j)}$ represents the cell line effect (in our case one parameter for each of the 3 cell lines in each plate), and $\beta_{\mathrm{Tr}(j)}$ represents the specific treatment/control effect (one parameter for each treatment and vehicle control used in that plate).

We then contrasted the treatment effect parameter $\beta_{\mathrm{Tr}(j)}$ to the appropriate matched control (β_{CO1}, β_{CO2} or β_{CO3}) using the default DEseq Wald test for each transcript, and a Benjamini-Hochberg (BH) adjusted p-value was calculated with automatic independent filtering (DEseq2 default setting). DE genes were determined as genes with at least one transcript having a Benjamini-Hochberg controlled FDR (*Benjamini and Hochberg, 1995*) (BH-FDR) of 10% and an absolute $\log_2$ fold-change value > 0.25. The same procedure was used for step 1 and step 2.

A summary of differential expression for both steps can be found in Tables S3, and S5, and a full set of differential expression results from step 2 can be found in Table S6.

8.2 Summary of 1st step DE analysis.

In step one, we used shallow RNA-seq (8.2 million reads/sample on average, Table S2) to coarsely characterize global changes in gene expression. To identify differentially expressed (DE) genes we used the method implemented in the software DEseq2 (*Love et al., 2014*) using gene annotations from Ensembl version 69, described in Section 8.1 (Figure S3, Table S3). This global characterization of transcriptional responses showed that certain treatments induce gene expression changes across all cell types (e.g. dexamethasone, retinoic acid), while others have a cell type specific effect (e.g. vitamin B6 in PBMCs) (Figure 1B). Of the 50 treatments considered, 16 do not induce strong changes in gene expression (defined here as $>$80 DE genes, 10% FDR, $|$logFC$|$ $>$0.25) in any cell type, while 8 result in strong gene expression changes across all cell types (dexamethasone, caffeine, tunicamycin, iron and manganese, vitamin D, aspirin, retinoic acid). Some of the more extreme responses, such as those corresponding to manganese, were determined to be a toxic response and were removed from any subsequent analysis.

8.3 Summary of 2nd step DE analysis.

32 treatment conditions with at least 80 DE genes from step one (see Section 8.2) were selected for deep sequencing. In addition, 12 treatment conditions with fewer than 80 DE genes were chosen to confirm that treatments with a small response from the shallow sequencing data similarly have a small response when deep sequenced. Overall, 297 samples (32 treatments and 3 controls across up to 5 cell types) were selected for repooling (Figure 1B, Table S4). Gene annotations from Ensembl version 75 were used, consisting of 204,940 transcripts corresponding to 60,234 unique ENSG gene identifiers. Sequencing reads covering transcripts were counted using `bedtools coverage`, using the `-s` and `-split` options to account for strandedness and for BED12 input, respectively.

8.4 Transcript and gene level FPKM summarization for each individual sample.

To perform Principal Component Analysis and network analysis, we calculated FPKMs for each sample (defined as the combination of a single individual and a single treatment, e.g., dexamethasone in GM19239) from the number of reads covering each transcript. To control for potential confounders, we fit the following linear model:

$$log10(\text{FPKM}_i + 1 \times 10^{-6}) \sim S_i + L_i + S_i * L_i \quad (4)$$

where for each transcript i, S_i is the transcript GC content proportion, L_i is the transcript length, and $S_i * L_i$ is an interaction term between GC content and transcript length. The residual of the linear model, the GC-corrected $\log_{10}$ FPKMs are then quantile normalized within each individual.

For analyses at the gene level, expression of a single transcript was chosen to represent each gene for each different cell type. The most highly expressed transcript (based on average expression across the cell type before quantile normalization) was selected from each gene to represent the overall expression of that gene.

After quantile normalization $\log_{10}$ FPKMs were further corrected within each individual by subtracting that individual's average value per transcript across all treatments. This was calculated after removing the top and bottom 10%-iles of data, usually referred to as 10% trimmed mean or Tukey's mean. The procedure is implemented in R "mean" function using the "trim=0.1" option.

8.5 Hierarchical clustering and PCA

Principal component analysis and hierarchical clustering was performed on a Pearson correlation matrix using transcript FPKM values for all samples on a plate (i.e., three individuals of a cell type treated with a single treatment panel, see Figure S1). Note that the input data is quantile normalized which makes Pearson correlation more similar to rank correlation in terms of robustness to outliers. Heatmaps were produced on the same correlation matrices, with dendrograms of Euclidian distance calculated using the "hclust" function with linkage "method=complete".

Samples corresponding to the same treatment tend to cluster together, and all controls (CO1, CO2, CO3) also cluster together (Figures S4 - S5). We see similar results when performing hierarchical clustering (Figures S4 - S5). Specifically, treatments that elicit strong responses cluster distinctly from control samples, and are often separated from other samples along the first or second PC (for example, selenium in HUVECs, dexamethasone and aldosterone in SMCs, tunicamycin in melanocytes – see Figures S6 and S4). In contrast, treatments that don't have a strong response cluster close with the controls (e.g., B vitamins in PBMCs, Figures S4 and S6). We also see clustering of treatments in biologically relevant ways. For example, in LCLs (Figure S4), PC1 separates the controls from the metal ions (selenium and copper), while PC2 separates the controls from nuclear receptor ligands (dexamethasone and vitamin A) and from caffeine. Finally, we also observe good concordance with the differential gene expression results. For example, vitamin D elicits a strong response in PBMCs, but less so in HUVECs (Figure 1B, also in FigureS12B). This is reflected in the fact that in PBMCs, vitamin D clusters apart

from other treatments (and furthest from the controls), while in HUVECs, vitamin D clusters with the controls (Figures S4, S6).

8.6 Network analysis with WGCNA

For network analysis we normalized the data as in the PCA procedure. We combined all the data across cell types, treatments and individuals resulting in a matrix with 14,527 rows (genes) and 297 columns (samples). We then used WGCNA (*Langfelder and Horvath, 2008*) version 1.47 implemented in R to build an unsigned network. A soft thresholding power of 6 was chosen, and the network was built using the automated blockwise modules pipeline using Pearson correlations, a signed topological overlap matrix, and a minimum module size of 10. Modules were cut from the network dendrogram with the Dynamic Hybrid Tree cut method, and the module eigengene was calculated as the first principal component of each module's expression matrix. A measure of module membership was calculated for each gene by correlating the gene's expression profile with its module's eigengene. Modules that did not have at least 3 genes with connection to the eigengene (> 0.5) were disbanded. Also, all genes with low connectivity to the module's eigengene (< 0.3) were removed from their modules. Modules with very highly correlated eigengenes were merged using WGCNA's default iterative clustering method. The minimum module size was set to 10, while the largest module contained 1,456 genes (median module size was 42 genes). A total of 7,936 genes, corresponding to 54.6% of the genes considered was assigned to a module (Table S7). The resulting coexpression network is made up of 87 modules and was found to approximate a scale free topology $R^2 = 0.8557$, indicating that the degree distribution (or measure of network connectivity) followed an expected power law. Each module eigengene was then evaluated for significant differences in expression in each cell type between treatment versus control using Student's t-test (Table S8). We used this t statistic to plot a heatmap of association of each eigengene to each cell-type/treatment combination (Figure S11). We then used a cytoscape version 3.2.1 to plot the global network (Figure 1C), and individual module networks that show different properties across treatments or cell types (Figure S12 and Figure S13).

We assigned a treatment to each module based on the most significant effect size of treatment on the module eigen-gene expression. Known target genes for specific treatments were categorized as hub genes in treatment-specific modules (Fig S12, Fig S13). For example, the known glucocorticoid targets, *TSC22D3* and *FKBP5*, are hub genes in Dexamethasone M66 and showed a similar transcriptional response in all analyzed cell types (Fig S13A). To identify the modules that capture cell type specific gene expression response to a treatment, we

considered seven treatments that were assayed in at least four cell types (dexamethasone, caffeine, selenium, vitamin A, aspirin, phthalate, and vitamin D) and are associated with 81 out of 87 modules (nominal $p < 0.01$). First, we examined specificity of each module across treatments and cell types. 33 of the 81 modules had only one cell type with a significant association in a given treatment while 42 of the 81 modules had significant association with one treatment in a cell type. By taking the overlap, we identified ten modules that are only associated with one environment, demonstrating that these ten modules contain genes that respond specifically to treatment in a particular cell type. Then, we analyzed modules significantly associated with a treatment in at least two cell types (59% of the modules) to investigate the extent each module represents similar patterns of gene expression responses across cell types. Overall, 88% of the modules (42 of 48 modules) exhibited similar gene expression response patterns across cell types with sharp differences being treatment-dependent. Of the five cell types, SMCs expressed the most treatment-specific responses with 3/5 modules showing opposite gene expression patterns across treatments.

9 Allele specific expression analysis

9.1 Core set of SNPs for analysis

To create a core set of SNPs for ASE analysis, we started with the autosomal 1KG SNPs from the phase 3 release (v5b.20130502, downloaded on 08/24/15), and first removed SNPs with low minor allele frequency (MAF < 5%). We also removed SNPs within regions of annotated copy number variation and ENCODE blacklisted regions (*Degner et al., 2012*), leaving a total of 7,085,180 SNPs in the core set.

9.2 Joint genotyping

Counts of reads covering each allele at selected SNPs (Section 9.1) were obtained by "piling up" aligned reads for each sample over SNPs using `samtools mpileup` and the hg19 human reference genome. Reads with a SNP at the beginning or at the end of the read were also removed to avoid any potential bias, as well as those within a reference skip (i.e., within a splice junction, meaning the read does not actually cover the SNP). All sample pileups for a given individual across all treatment conditions and the two treatment plates were processed together (not merged) using the QuASAR package (*Harvey et al., 2015*) for joint genotyping. For each individual, SNPs with less than 15 reads across all conditions were removed as the genotype would not be reliable. Compared to the DEseq2 analysis, technical replicate libraries for the same vehicle controls and plate are merged together.

To verify that none of the samples had been contaminated with reads from another individual during library preparation, we compared the allele ratio, $\hat{\rho}$, across samples processed at the same time (based on our study design these are samples from the same cell type, see Figure S1). The allele ratio is indicative of the genotype, as it follows a trimodal distribution with peaks for homozygous reference and alternate, and heterozygous (*Harvey et al., 2015*). For each cell type, we examined the $\hat{\rho}$ for SNPs covered in each of the three individuals across all samples. For each cell type,the $\hat{\rho}$ values were correlated across the three individuals, and the resulting correlation matrix used to perform PCA (Figure S8). We expect the three individuals to group into three distinct clusters on the PCA plot, in the absence of sample contamination or mix-up. This clustering is what we observe for all samples, confirming that there is no cross-individual contamination.

9.3 ASE Inference

ASE inference was performed for each sample separately. Using read count and genotyping data from Section 9.2, heterozygous SNPs were tested for ASE using QuASAR. Briefly, all heterozygous SNPs with a posterior probability of being heterozygous higher than 0.99 and a read coverage $>$40 reads were selected. To account for overdispersion, we calculated the M_s hyper parameter of the Beta-binomial model separately within bins of read coverage for each sample. SNPs were separated into the following eight bins: [40, 50), [50, 60), [60, 80), [80, 100), [100, 250), [250, 500), [500, 1000), [1000, 100000). We noted that the overdispersion decreases (i.e. M increases) corresponding to bins with more depth of coverage (Figure S9). The inference step takes into account the appropriate dispersion estimate for a given SNP's coverage, technical noise of that sample, and the genotyping uncertainty estimate (calculated in 9.2), and tests for the possibility that the allele ratio (ρ, or the proportion of reference reads to total reads) is different than 0.5. At the same time it also gives an estimate of ρ in log-odds form, $\hat{\beta} = \log(\rho/(1-\rho))$ that can be interpreted as log(reference reads / alternate reads) while taking into account overdispersion and genotyping error. We also obtain an estimate of the standard error for $\hat{\beta}$, that we can use for downstream analyses of cASE. A summary of the amount of ASE detected in each sample is in Figure S10 and Table S9. A full list of SNPs tested can be found in Table S10. In the 89 treatment conditions, we identified 11,305 instances of ASE (10% FDR, Fig 2), corresponding to 1,455 unique ASE genes out of 11,990 genes with heterozygous sites we interrogated. In an individual sample, 0.92% of expressed genes with heterozygous SNPs are ASE genes, on average.

To calculate a SNP-based expression and fold change (rather than gene-based, as in the FPKM (Section 8.4)

and DESeq2 (Section 8.1) methods), we first calculated the read coverage at each SNP, adjusted by the sequence depth of the sample:

$$\text{TPM}_{ij} = R_{ij} * 10^6 / D_j \tag{5}$$

where the transcripts per million (TPM) for SNP i in sample j is calculated as the read coverage R_{ij} times 10^6, divided by the sequence depth of the sample, D_j. The average expression level at a SNP is then calculated as the average between treatment T and control C samples:

$$\text{Avg. TPM} = \frac{log_2(\text{TPM}_T) + log_2(\text{TPM}_C)}{2} \tag{6}$$

Similarly, the SNP-based fold change was calculated as:

$$log_2(\text{FC}) = log_2(\text{TPM}_T / \text{TPM}_C) \tag{7}$$

10 Conditional ASE analysis

Most of the ASE signal is shared between treatment and control for a given SNP (dots along the $y = x$ line in Figure 3D). However, there is evidence for SNPs showing ASE only in the control (along the x-axis) or ASE only in the treatment condition (along the y-axis). These SNPs represent candidates for conditional ASE (cASE): SNPs that display ASE only in certain environmental conditions. Additionally, there is a large number of SNPs falling in the space comprised between the $y = x$ line and the axes. These SNPs may represent cases of shared-ASE with large overdispersion or cases of cASE with quantitative differences in the genetic effect across conditions. In general testing for differences in genetic effects across two conditions is particularly challenging as it implies comparing two noisy measurements to determine whether they are different while taking into account heterogeneity of the underlying true genetic effects.

This problem has been previously faced in the context of condition-specific eQTL mapping (e.g. reQTL mapping and tissue-specific eQTL mapping) (*Mangravite et al., 2013, Maranville et al., 2013, Qiu et al., 2014, Maranville et al., 2012, Maranville et al., 2011, Çalışkan et al., 2015, Barreiro et al., 2012, Lee et al., 2014, Siddle et al., 2014, Franco et al., 2013, Idaghdour et al., 2012, Fairfax et al., 2014, Flutre et al., 2013*) and some of the approaches developed can be broadly translated to applications for cASE analysis. With regard to

condition-specific eQTL mapping, three major approaches have been used so far: i) independent eQTL calling and comparing p-values, ii) comparing summary statistics using a meta-analysis approach that takes into account heterogeneity of the sub-groups, and iii) directly modeling the interaction term using ANOVA or QTL mapping of the fold-change.

In the first approach, independent eQTL are determined in the two conditions analyzed, and the p-values for each SNP compared across conditions. The SNP is defined then as an eQTL only in the condition with the lowest p-value (*Fairfax et al., 2014*). A major limitation of this approach is that higher p-values may result from incomplete power in any of the conditions analysed. Another modification of this approach is based on setting different FDR thresholds across conditions so that a SNP is assigned to condition 1 if it has a very low FDR in that condition (e.g.$<1\%$) and a very high FDR in condition 2 (FDR$>90\%$) (*Barreiro et al., 2012*).

The second class of approaches directly compares different eQTL configurations in a Bayesian framework (*Maranville et al., 2011, Wen and Stephens, 2014*) and incorporates in the model heterogeneity in the effect sizes between the groups contrasted. This class of models also defines the genetic effect of the response eQTL in a strict on/off mode between the groups compared.

Finally the third class of approaches uses a linear model with an interaction term to directly test for gene-by-environment interactions at a given gene and SNP (*Çalışkan et al., 2015*), and has been also recently applied to GxE analysis with ASE data (*Knowles et al., 2015*). This class of approaches includes eQTL mapping of gene expression log-fold change calculated across the two conditions tested (*Smirnov et al., 2009, Barreiro et al., 2012, Maranville et al., 2011*). As opposed to the Bayesian approaches they do not provide information on the specific eQTL configuration, they theoretically allow capturing any type of eQTL configuration, although they have the greatest power for eQTLs with opposite genetic effects in the two conditions tested and may be confounded with increased group heterogeneity.

To test for cASE, here we used two methods that belong to the classes of approaches described in 2 and 3 above. The first one is MeSH (Meta-analysis of Subgroup Heterogeneity), which identifies qualitative GxE interactions. We also developed a test for cASE that compares the evidence for ASE in the treatment to ASE in the control using the $\hat{\beta}$ estimates from QuASAR directly, thus identifying quantitative GxE interactions. We refer to this second method as ΔAST, Differential Allele-Specific Test.

10.1 Meta-analysis of subgroup heterogeneity (MeSH)

When testing for genetic associations such as cASE, it is important to consider subgroup heterogeneity. Specifically, a joint analysis that allows for heterogeneity can yield stronger signals than analyzing each subgroup separately. Here, we used MeSH to model potentially heterogeneous cASE effects across multiple subgroups contained within the data. MeSH uses a Bayesian approach to contrast 4 possible configurations: no ASE in either the treatment or the control, ASE in both the treatment and the control (”shared ASE”), ASE in the treatment only, and ASE in the control only, the latter two categories being cASE. To detect qualitative differences in the ASE signal between treatment and control samples, we used a modified version of MESH that quantifies the amount of heterogeneity in the genetic effect across pairs of conditions. The input to MeSH is a pair of ASE observations derived from QuASAR summarized by the the parameter β measuring the allelic imbalance and a standard error of the parameter. In our case, for each heterozygous SNP and individual, we pair the ASE observed in a treatment with the corresponding vehicle control on the same individual and plate. This results in 763,762 QuASAR ASE treatment/control measurement pairs (31,214 unique heterozygous SNPs). MeSH then uses a hierarchical model to characterize the ASE effects β and the heterogeneity across all observations. Then, a Bayes factor is derived contrasting each of the configurations that assume ASE in at least one of the conditions to the configuration with no ASE in either condition as baseline. Specifically, $BF_{treatment}$ contrasts the evidence for $\beta_t \neq 0, \beta_c = 0$ to $\beta_t = 0, \beta_c = 0$; $BF_{control}$ contrasts the evidence for $\beta_t = 0, \beta_c \neq 0$ to $\beta_t = 0, \beta_c = 0$, and BF_{shared} contrasts the evidence for $\beta_t \neq 0, \beta_c \neq 0$ to $\beta_t = 0, \beta_c = 0$. To look specifically at conditional ASE, a Bayes factor for cASE is calculated as $BF_{treatment} - BF_{shared}$ (treatment-only cASE) and $BF_{control} - BF_{shared}$ (control-only cASE). SNPs with cASE are then identified as SNPs with $BF_{cASE} > 30$.

10.2 ΔAST: A novel method to measure cASE

The cASE models tested in MESH assume extreme ASE differences between treatment and control. However, extreme on/off cases of ASE may not be the only relevant ones as cASE may also exist where the genetic effect (β) differs to a significant degree between treatment and control ($\beta_T - \beta_C \neq 0$) but is non-zero in both conditions. To capture cASE genes that may not fit perfectly to the models tested by MESH, we developed an alternative approach named ΔAST to identify quantitative differences in the ASE ratios between treatment and control.

Differential Z-scores (Z_Δ) were calculated from QuASAR betas using the following formula. For each SNP,

$$Z_\Delta = \frac{\beta_T - \beta_C}{\sqrt{se_T^2 + se_C^2}} \quad (8)$$

where β_T and se_T represent the beta and standard error estimates for the treatment condition, and β_C and se_C represent the beta and standard error estimates for the control condition. The Z_Δ scores were then normalized by the standard deviation across Z_Δ scores corresponding to control vs control (CO1 vs. CO2). Finally p-values (p_Δ) are calculated from the Z_Δ scores as $p_\Delta = 2 \times \text{pnorm}(-|z|)$. Under the null, Z_Δ are asymptotically normally distributed and Figure 3E shows that when contrasting the two sets of controls the p_Δ-values are almost uniformly distributed as expected. To further correct for this small deviation we use the control vs. control p-values to empirically estimate the FDR as detailed in the following section.

10.3 Empirical estimation of the cASE false discovery rate

For ΔAST analysis of cASE, we were also able to take advantage of our study design that includes at least two pairs of vehicle controls per individual (CO1 and CO2) in each treatment plate. This allowed us to apply our statistical methods to a subset of control data, contrasting within each individual and plate the two controls (CO2 to CO1), and to empirically determine the false discovery rate (FDR) or to recalibrate the p-values. This is very similar to a permutation procedure to estimate the p-value in eQTL studies. Permuting read assignments in ASE studies may not recapitulate the overdispersed nature of the allelic imbalances and there is a small number of permutations possible across individuals. Nevertheless, in our experimental design, contrasting the two control samples could be used to empirically reveal the underlying null distribution similar to the permutation based approaches that are possible in QTL studies with big sample sizes.

To this end, we ran ΔAST analysis on 120,273 SNPs, contrasting the two controls (CO2 to CO1) as we did for any of the treatments to its matched control (Tx to CO1 or Tx to CO2). To focus on actual false positives, we removed 8,040 control SNPs with a SNP-based log_2(fold change) > 1 (see Section 9.3) in the CO2 versus CO1) comparison (see Table S12 for results of the CO2 vs CO1 analysis). For our test statistics for cASE (i.e., Z_Δ), we can observe the empirical distribution obtained when contrasting the two sets of controls. This empirical distribution represents a sample from the null distribution. Using this empirical distribution we can then derive a corrected p-value based on the ranks observed in the control vs. control (this is exactly the same as in a permutation based approach). After calculating the corrected p-values derived from Z_Δ, we applied multiple test

correction using the q-value method (*Storey, 2003*).

We identified 67 cASE SNPs using the ΔAST method, corresponding to an FDR of 10%. When we relax the FDR threshold (25%), we find a total of 178 cASE SNPs (corresponding to 160 unique genes), of which 68 SNPs are identified also with MeSH. The list of significant cASE SNPs is in Table S11 (also see Figure 4). Of the 31 genes with dexamethasone reQTLs previously identified in 116 LCL and 88 PBMC samples (*Maranville et al., 2011, Maranville et al., 2013*) and tested in our dataset, here we validated 21 genes with cASE (p <0.05), 6 of which in response to dexamethasone, including the population-specific reQTL gene *NQO1* (p = 0.03).

When we considered all cASE SNPs, we observed a significant positive correlation between the gene expression log_2(fold change) after treatment and differences in the genetic effect in treatment and control samples (Pearson's r = 0.204, p-value = 0.038, Figure 5A) . However, we observed a negative correlation between gene expression levels and the difference in the genetic effect in the treatment and the control samples (Pearson's r = -0.381, p-value = 6.7×10^{-5}, Figure 5B).

10.4 Analysis of environmental displacement of genetic effects (EDGE)

In addition to characterizing significant differences in the allelic ratio parameters (β) between treatment and control to determine cASE, we also characterized how correlated they are across all heterozygous sites. This correlation measures the consistency of the genetic effect between the treatment and control, and therefore a lower correlation indicates a higher perturbation or displacement of the genetic effects, in our case the ASE β parameters estimated by QuASAR. Within each treatment and cell line subgroup, we examined the Pearson's correlation of the treatment standardized effect size (ASE Z_T=β_T/se_T) to the matched control one (ASE Z_C=β_C/se_C). To make the measurement more comparable across environments, we normalized the treatment-control correlation to the correlation observed between the two controls (CO1 and CO2) as measured within the same cell type (see Figure S19A-B). Formally, we define this as the environmental displacement of genetic effect (EDGE) index:

$$\mathrm{EDGE}_{s,t} = \frac{\mathrm{Pearson}(Z_{s,\mathrm{CO1}}, Z_{s,\mathrm{CO2}})}{\mathrm{Pearson}(Z_{s,t}, Z_{s,c})} \tag{9}$$

where Pearson($Z_{s,t}$, $Z_{s,c}$) is the sample Pearson correlation coefficient between treatment t and control c ASE Z-scores across all observed SNPs in cell type s. Equivalently, Pearson($Z_{s,\mathrm{CO1}}$, $Z_{s,\mathrm{CO2}}$) is the Pearson correlation coefficient between the two controls sets ASE Z-scores across all observed SNPs in cell type s. These values can be found in Figure S19C, Figure S20, and Table S14.

We also asked whether the EDGE index was fully explained by the SNPs with significant cASE. While the EDGE index and the proportion of tests with cASE are very well correlated (see Figure S19C), removing significant SNPs did not substantially alter the EDGE index (Figure S21) indicating that there may remain a similar fraction of cASE SNPs with a smaller effect size that we are not able to detect and a higher coverage may be necessary.

10.5 Identification of induced ASE

Conditional ASE analyses require that for both treatment and control conditions the SNP has a sufficiently high coverage (Section 9.3), resulting in the somewhat implicit requirement that the gene containing that SNP is expressed both in treatment and control conditions. However, many genes may have very low expression levels for the control condition and very high expression with ASE in the treatment. In other words, the expression of these genes would be induced by a specific treatment. For these cases, ASE in the control condition is not well defined, as it is exemplified in the extreme case where 0:0 reads are counted for both alleles. We denote this type of phenomenon as induced ASE (iASE), which are cases when the ASE can only be observed in genes that are induced by the treatment. Genes with iASE may be as interesting as genes with cASE and would probably be missed by studies that only consider baseline eQTLs or ASE. Physiologically, the iASE allele with high expression may exceed the threshold for a downstream effect on an observable cellular or organismal phenotype that may only manifest in a particular environmental condition.

To identify genes with iASE, we selected SNPs that were well covered in the treatment (i.e., $>$40 reads) and had ASE (10% FDR) but had little to no expression in the matched control. We used a coverage threshold in the control of $10 \times (D_C/D_T)$, where D_C and D_T represent the sequencing depth of the control and treatment libraries, respectively in TPM (see Section 9.3). This equates to a ratio of 40 reads to 10 (expression in the control is 4-fold lower than the minimum required for a gene to be considered expressed in the ASE analysis), while accounting for sequencing depth differences. Finally, we required the SNP-based $\log_2$(fold change) (Section 9.3) to be greater than $\log_2$(5). This threshold represents a strong difference between treatment and control expression, corresponding to the 0.13 percentile of fold change values for all SNPs tested for cASE. Using this criteria, of 6,817 SNPs with ASE in 2,868 genes not expressed at baseline, we identified 75 iASE SNPs (10% FDR) corresponding to 60 unique genes (Table S15).

10.6 Analysis of heritability enrichment using GEMMA

GEMMA (Genome-wide Efficient Mixed Model Association) (*Zhou and Stephens, 2012,Zhou, 2016*) tests for the proportion of variance in phenotypes explained (PVE) by typed genotypes, for example, ”chip heritability” (*Yang et al., 2010*). This program implements a univariate linear mixed model (LMM) for marker association tests with a phenotype of interest (i.e., GWAS traits) that takes into account population stratification and sample structure. Similar to other methods that partition heritability estimates across SNP functional categories (*Gusev et al., 2014*), GEMMA estimates fold change, to contrast heritability per SNP in one category with heritability per SNP across the genome. In particular, the enrichment in ith category = (heritability per SNP in category i)/(heritability per SNP in all categories). We used GEMMA to jointly analyze summary statistics from 18 GWAS meta-analysis studies with annotations of regulatory variation.

To run GEMMA we first annotated SNPs to the closest gene, using bedtools closest. We then partitioned SNPs genome wide to create a category file. Each SNP was assigned to one of the following categories: cASE (genic regions with conditional allele specific expression) or iASE (genic regions with induced allele specific expression), ASE (genic regions with allele specific expression), Other Genic (genic regions that do not fall into any of the categories above) and Intergenic (greater than 100kb from any gene). Here genic regions are defined as the gene body $+100$ kb on both sides of the gene. This genomic definition was chosen to be inclusive of the minimum regulatory region usually tested in eQTL studies. We then used GEMMA to compute the SNP correlations among different categories from a reference panel (502 individuals of European ancestry from the 1000 genome project). This was followed by summing the z^2 statistics from the GWAS meta-analysis with the categories. Finally, we computed the proportion of variance, and fold enrichment of heritability explained by each category. A table of the results can be found in Table S17.

10.7 Analysis of SNPs in the GWAS catalog

We downloaded the GWAS catalog (*Welter et al., 2014*) (version 1.0.1) on January 5th, 2016. To identify the overlap between our annotations and those associated with a GWAS trait, we intersected the unique genes of interest from our data with the reported genes from the GWAS catalog. Of the genes expressed in our dataset, 32,451 genes are differentially expressed in at least one treatment condition while 7,734 genes are not. 22% of DE genes (7,010 genes) are identified in the GWAS catalog while only 4% of non-differentially expressed genes

(292 genes) are previously reported in the GWAS catalog. Using a Fisher's exact test on a 2x2 contingency table, we found an enrichment of DE genes among the genes reported to be associated with a trait in the GWAS catalog ($p < 2.2 \times 10^{-16}$, OR = 7.0).

We further studied whether we find an enrichment of genes containing cASE or iASE among genes associated with organismal traits. Of the 215 genes that contain iASE or cASE, 105 genes (49%) were found in the GWAS catalog. When compared to DE genes (after removing genes that contain either iASE or cASE, 6,906 were found in GWAS while 25,331 were not) using a Fisher's exact test, we found a significant enrichment of iASE/cASE genes among GWAS genes ($p < 2.2 \times 10^-16$, OR = 3.5). We also found that when compared to genes with ASE (not iASE or cASE), genes containing iASE or cASE have 1.4 higher odds to be relevant for complex traits (p = 0.025).

To assess our results relevant to other studies, we compared our iASE and cASE genes to eGenes, genes associated with an eQTL, from the GTEx study (*The GTEx Consortium, 2015*). 194 iASE or cASE genes were also found to be eGenes leaving 26,899 eGenes without evidence of GxE in our study. We found, again, that iASE and cASE genes were enriched among genes associated with organismal traits with 50% in the GWAS catalog (97 genes) as compared to 24% eGenes (6,419 genes) (Fisher's exact test, $p = 4.3 \times 10^-15$, OR = 3.2). We also repeated these enrichment analyses excluding the iASE cases, but we essentially obtain the same odd ratios and with a significant enrichment for cASE genes.

10.8 Examples of cASE/iASE in GWAS genes with a biological connection

One example of iASE that occurs following treatment with caffeine is in the gene *GIPR* associated with obesity-related traits (*Wen et al., 2014, Mahajan et al., 2014, Berndt et al., 2013, Fox et al., 2012, Okada et al., 2012, Wen et al., 2012, Speliotes et al., 2010, Saxena et al., 2010*). Previous studies have shown that caffeine can help prevent or treat obesity (*Ohara et al., 2016, Kim et al., 2016, Li et al., 2015*). This iASE links the effect of caffeine to obesity through *GIPR*. The direction of the effect, with the low-BMI allele being in phase with the high expression allele, is in agreement with a study showing that *GIPR* expression is lower in obese patients (*Ceperuelo-Mallafré et al., 2014*). Another example of iASE occurs at rs4619 in *IGFBP1* following dexamethasone treatment. *IGFBP1* is a gene that interacts with insulin-like growth factors and promotes cell migration. *IGFBP1* has also been associated with rheumatoid arthritis and major depressive disorder in genome wide association studies (*GENDEPInvestigators et al., 2013, Padyukov et al., 2011*). These two disorders are seemingly unrelated until

we consider that dexamethasone is a drug often used to alleviate rheumatoid arthritis (*BUNIM et al., 1958, Ferreira et al., 2016*) while glucocorticoids are often misregulated in depression (*Zunszain et al., 2011, Mokhtari et al., 2013, Carvalho and Pariante, 2008*). Here, our data suggests that *IGFBP1* may be associated with these very different phenotypes because it participates in one of the glucocorticoid response pathways.

We identified cASE following copper treatment at rs7587 in *SAMM50*, a gene associated with liver enzyme levels and non-alcoholic fatty liver disease (*Kitamoto et al., 2013, Kawaguchi et al., 2012, Chambers et al., 2011, Kamatani et al., 2010, Yuan et al., 2008*). Reduced copper levels is associated with non-alcoholic fatty liver disease and severe hepatic steatosis (*Feldman et al., 2015, Aigner et al., 2010*). Our data suggest that copper facilitates these liver responses through modulation of allele-specific expression in *SAMM50*. A missense SNP (rs17482078) in *ERAP1* showed cASE in response to selenium treatment (*ERAP1* is shown in a selenium response module in Fig S14). This SNP is associated with Behçet's disease (*Kirino et al., 2013*), an autoimmune disorder. Individuals with Behçet's disease have lower selenium levels (*Delilbaşi et al., 1991*). Another example of cASE was identified at rs189899 in the gene, *GOSR2*, following treatment with mono-n-butyl phthalate. *GOSR2* is associated with blood pressure regulation (*Ehret et al., 2011, Wain et al., 2011*). As phthalate exposure is also associated with increased blood pressure (*Trasande et al., 2013*), phthalate may influence blood pressure through ASE modulation in *GOSR2*.

References

Aigner et al., 2010. Aigner, E., Strasser, M., Haufe, H., Sonnweber, T., Hohla, F., Stadlmayr, A., Solioz, M., Tilg, H., Patsch, W., Weiss, G., *et al.*, 2010. A role for low hepatic copper concentrations in nonalcoholic Fatty liver disease. *The American Journal of Gastroenterology*, **105**(9):1978–85.

Barreiro et al., 2012. Barreiro, L. B., Tailleux, L., Pai, A. A., Gicquel, B., Marioni, J. C., and Gilad, Y., 2012. Deciphering the genetic architecture of variation in the immune response to Mycobacterium tuberculosis infection. *Proceedings of the National Academy of Sciences*, **109**(4):1204–9.

Benjamini and Hochberg, 1995. Benjamini, Y. and Hochberg, Y., 1995. Controlling the False Discovery Rate: A Practical and Powerful Approach to Multiple Testing. *Journal of the Royal Statistical Society*, **57**(1):289 – 300.

Berndt et al., 2013. Berndt, S. I., Gustafsson, S., Mägi, R., Ganna, A., Wheeler, E., Feitosa, M. F., Justice, A. E., Monda, K. L., Croteau-Chonka, D. C., Day, F. R., *et al.*, 2013. Genome-wide meta-analysis identifies 11 new loci for anthropometric traits and provides insights into genetic architecture. *Nature Genetics*, **45**(5):501–12.

BUNIM et al., 1958. BUNIM, J. J., BLACK, R. L., LUTWAK, L., PETERSON, R. E., and WHEDON, G. D., 1958. Studies on dexamethasone, a new synthetic steroid, in rheurheumatoid arthritis: a preliminary report; adrenal cortical, metabolic and early clinical effects. *Arthritis and Rheumatology*, **1**(4):313–331.

Çalışkan et al., 2015. Çalışkan, M., Baker, S. W., Gilad, Y., and Ober, C., 2015. Host genetic variation influences gene expression response to rhinovirus infection. *PLoS Genetics*, **11**(4):e1005111.

Carvalho and Pariante, 2008. Carvalho, L. A. and Pariante, C. M., 2008. In vitro modulation of the glucocorticoid receptor by antidepressants. *Stress*, **11**(6):411–424.

Ceperuelo-Mallafré et al., 2014. Ceperuelo-Mallafré, V., Duran, X., Pachón, G., Roche, K., Garrido-Sánchez, L., Vilarrasa, N., Tinahones, F. J., Vicente, V., Pujol, J., Vendrell, J., *et al.*, 2014. Disruption of gip/gipr axis in human adipose tissue is linked to obesity and insulin resistance. *The Journal of Clinical Endocrinology and Metabolism*, **99**(5):E908–E919. PMID: 24512489.

Chambers et al., 2011. Chambers, J. C., Zhang, W., Sehmi, J., Li, X., Wass, M. N., Van der Harst, P., Holm, H., Sanna, S., Kavousi, M., Baumeister, S. E., *et al.*, 2011. Genome-wide association study identifies loci influencing concentrations of liver enzymes in plasma. *Nature Genetics*, **43**(11):1131–1138.

Degner et al., 2012. Degner, J. F., Pai, A. A., Pique-Regi, R., Veyrieras, J.-B., Gaffney, D. J., Pickrell, J. K., De Leon, S., Michelini, K., Lewellen, N., Crawford, G. E., *et al.*, 2012. DNase I sensitivity QTLs are a major determinant of human expression variation. *Nature*, **482**(7385):390–4.

Delilbaşi et al., 1991. Delilbaşi, E., Turan, B., Yücel, E., Saşmaz, R., Işimer, A., and Sayal, A., 1991. Selenium and Behçet's disease. *Biological Trace Element Research*, **28**(1):21–5.

Dobin et al., 2013. Dobin, A., Davis, C. a., Schlesinger, F., Drenkow, J., Zaleski, C., Jha, S., Batut, P., Chaisson, M., and Gingeras, T. R., 2013. STAR: ultrafast universal RNA-seq aligner. *Bioinformatics*, **29**(1):15–21.

Ehret et al., 2011. Ehret, G. B., Munroe, P. B., Rice, K. M., Bochud, M., Johnson, A. D., Chasman, D. I., Smith, A. V., Tobin, M. D., Verwoert, G. C., Hwang, S.-J., *et al.*, 2011. Genetic variants in novel pathways influence blood pressure and cardiovascular disease risk. *Nature*, **478**(7367):103–9.

Fairfax et al., 2014. Fairfax, B. P., Humburg, P., Makino, S., Naranbhai, V., Wong, D., Lau, E., Jostins, L., Plant, K., Andrews, R., McGee, C., *et al.*, 2014. Innate immune activity conditions the effect of regulatory variants upon monocyte gene expression. *Science*, **343**(6175):1246949.

Feldman et al., 2015. Feldman, A., Aigner, E., Weghuber, D., and Paulmichl, K., 2015. The Potential Role of Iron and Copper in Pediatric Obesity and Nonalcoholic Fatty Liver Disease. *BioMed Research International*, **2015**:287401.

Ferreira et al., 2016. Ferreira, J. F., Ahmed Mohamed, A. A., and Emery, P., 2016. Glucocorticoids and rheumatoid arthritis. *Rheumatic Diseases Clinics of North America*, **42**(1):33–46.

Flutre et al., 2013. Flutre, T., Wen, X., Pritchard, J., and Stephens, M., 2013. A statistical framework for joint eQTL analysis in multiple tissues. *PLoS Genetics*, **9**(5):e1003486.

Fox et al., 2012. Fox, C. S., Liu, Y., White, C. C., Feitosa, M., Smith, A. V., Heard-Costa, N., Lohman, K., Johnson, A. D., Foster, M. C., Greenawalt, D. M., *et al.*, 2012. Genome-wide association for abdominal subcutaneous and visceral adipose reveals a novel locus for visceral fat in women. *PLoS Genetics*, **8**(5).

Franco et al., 2013. Franco, L. M., Bucasas, K. L., Wells, J. M., Niño, D., Wang, X., Zapata, G. E., Arden, N., Renwick, A., Yu, P., Quarles, J. M., *et al.*, 2013. Integrative genomic analysis of the human immune response to influenza vaccination. *eLife*, **2**:e00299.

GENDEPInvestigators et al., 2013. GENDEPInvestigators, MARSInvestigators, and STAR*DInvestigators, 2013. Common genetic variation and antidepressant efficacy in major depressive disorder: a meta-analysis of three genome-wide pharmacogenetic studies .

Gusev et al., 2014. Gusev, A., Lee, S. H., Trynka, G., Finucane, H., Vilhjálmsson, B. J., Xu, H., Zang, C., Ripke, S., Bulik-Sullivan, B., Stahl, E., *et al.*, 2014. Partitioning heritability of regulatory and cell-type-specific variants across 11 common diseases. *The American Journal of Human Genetics*, **95**(5):535–552.

Harvey et al., 2015. Harvey, C. T., Moyerbrailean, G. A., Davis, G. O., Wen, X., Luca, F., and Pique-Regi, R., 2015. QuASAR: quantitative allele-specific analysis of reads. *Bioinformatics*, **31**(8):1235–1242.

Idaghdour et al., 2012. Idaghdour, Y., Quinlan, J., Goulet, J.-P., Berghout, J., Gbeha, E., Bruat, V., de Malliard, T., Grenier, J.-C., Gomez, S., Gros, P., *et al.*, 2012. From the Cover: Feature Article: Evidence for additive and interaction effects of host genotype and infection in malaria. *Proceedings of the National Academy of Sciences*, **109**(42):16786–16793.

Kam et al., 2013. Kam, R. K. T., Shi, W., Chan, S. O., Chen, Y., Xu, G., Lau, C. B.-S., Fung, K. P., Chan, W. Y., and Zhao, H., 2013. Dhrs3 protein attenuates retinoic acid signaling and is required for early embryonic patterning. *The Journal of Biological Chemistry*, **288**(44):31477–87.

Kamatani et al., 2010. Kamatani, Y., Matsuda, K., Okada, Y., Kubo, M., Hosono, N., Daigo, Y., Nakamura, Y., and Kamatani, N., 2010. Genome-wide association study of hematological and biochemical traits in a Japanese population. *Nature Genetics*, **42**(3):210–215.

Kawaguchi et al., 2012. Kawaguchi, T., Sumida, Y., Umemura, A., Matsuo, K., Takahashi, M., Takamura, T., Yasui, K., Saibara, T., Hashimoto, E., Kawanaka, M., *et al.*, 2012. Genetic polymorphisms of the human pnpla3 gene are strongly associated with severity of non-alcoholic fatty liver disease in japanese. *PLoS One*, **7**(6):e38322.

Kim et al., 2016. Kim, H. J., Yoon, B. K., Park, H., Seok, J. W., Choi, H., Yu, J. H., Choi, Y., Song, S. J., Kim, A., and Kim, J.-W., *et al.*, 2016. Caffeine inhibits adipogenesis through modulation of mitotic clonal expansion and the akt/gsk3 pathway in 3t3-l1 adipocytes. *BMB Reports*, **49**(2):111–5.

Kirino et al., 2013. Kirino, Y., Bertsias, G., Ishigatsubo, Y., Mizuki, N., Tugal-Tutkun, I., Seyahi, E., Ozyazgan, Y., Sacli, F. S., Erer, B., Inoko, H., *et al.*, 2013. Genome-wide association analysis identifies new susceptibility loci for Behçet's disease and epistasis between HLA-B*51 and ERAP1. *Nature Genetics*, **45**(2):202–7.

Kitamoto et al., 2013. Kitamoto, T., Kitamoto, A., Yoneda, M., Hyogo, H., Ochi, H., Nakamura, T., Teranishi, H., Mizusawa, S., Ueno, T., Chayama, K., *et al.*, 2013. Genome-wide scan revealed that polymorphisms in the PNPLA3, SAMM50, and PARVB genes are associated with development and progression of nonalcoholic fatty liver disease in Japan. *Human Genetics*, **132**(7):783–792.

Knowles et al., 2015. Knowles, D. A., Davis, J. R., Raj, A., Zhu, X., Potash, J. B., Weissman, M. M., Shi, J., Levinson, D., Mostafavi, S., Montgomery, S. B., *et al.*, 2015. Allele-specific expression reveals interactions between genetic variation and environment. *bioRxiv*, **10.1101/025874**.

Langfelder and Horvath, 2008. Langfelder, P. and Horvath, S., 2008. WGCNA: an R package for weighted correlation network analysis. *BMC Bioinformatics*, **9**:559.

Lee et al., 2014. Lee, M. N., Ye, C., Villani, A.-C., Raj, T., Li, W., Eisenhaure, T. M., Imboywa, S. H., Chipendo, P. I., Ran, F. A., Slowikowski, K., *et al.*, 2014. Common genetic variants modulate pathogen-sensing responses in human dendritic cells. *Science*, **343**(6175):1246980.

Li et al., 2015. Li, D.-K., Ferber, J. R., and Odouli, R., 2015. Maternal caffeine intake during pregnancy and risk of obesity in offspring: a prospective cohort study. *International Journal of Obesity*, **39**(4):658–664.

Li and Durbin, 2009. Li, H. and Durbin, R., 2009. Fast and accurate short read alignment with Burrows-Wheeler transform. *Bioinformatics*, **25**(14):1754–1760.

Love et al., 2014. Love, M. I., Huber, W., and Anders, S., 2014. Moderated estimation of fold change and dispersion for RNA-seq data with DESeq2. *Genome Biology*, **15**(12):550.

Mahajan et al., 2014. Mahajan, A., Go, M. J., Zhang, W., Below, J. E., Gaulton, K. J., Ferreira, T., Horikoshi, M., Johnson, A. D., Ng, M. C. Y., Prokopenko, I., *et al.*, 2014. Genome-wide trans-ancestry meta-analysis provides insight into the genetic architecture of type 2 diabetes susceptibility. *Nature Genetics*, **46**(3):234–44.

Mangravite et al., 2013. Mangravite, L. M., Engelhardt, B. E., Medina, M. W., Smith, J. D., Brown, C. D., Chasman, D. I., Mecham, B. H., Howie, B., Shim, H., Naidoo, D., *et al.*, 2013. A statin-dependent QTL for GATM expression is associated with statin-induced myopathy. *Nature*, **502**(7471):377–80.

Maranville et al., 2013. Maranville, J. C., Baxter, S. S., Witonsky, D. B., Chase, M. A., and Di Rienzo, A., 2013. Genetic mapping with multiple levels of phenotypic information reveals determinants of lymphocyte glucocorticoid sensitivity. *American Journal of Human Genetics*, **93**(4):735–43.

Maranville et al., 2011. Maranville, J. C., Luca, F., Richards, A. L., Wen, X., Witonsky, D. B., Baxter, S., Stephens, M., and Rienzo, A., 2011. Interactions between glucocorticoid treatment and *cis*-regulatory polymorphisms contribute to cellular response phenotypes. *PLoS Genetics*, **7**(7):e1002162.

Maranville et al., 2012. Maranville, J. C., Luca, F., Stephens, M., and Di Rienzo, A., 2012. Mapping gene-environment interactions at regulatory polymorphisms: insights into mechanisms of phenotypic variation. *Transcription*, **3**(2):56–62.

Mokhtari et al., 2013. Mokhtari, M., Arfken, C., and Boutros, N., 2013. The dex/crh test for major depression: a potentially useful diagnostic test. *Psychiatry Research*, **208**(2):131–139.

Moyerbrailean et al., 2015. Moyerbrailean, G. A., Davis, G. O., Harvey, C. T., Watza, D., Wen, X., Pique-Regi, R., and Luca, F., 2015. A high-throughput rna-seq approach to profile transcriptional responses. *Scientific Reports*, **5**:14976.

Ohara et al., 2016. Ohara, T., Muroyama, K., Yamamoto, Y., and Murosaki, S., 2016. Oral intake of a combination of glucosyl hesperidin and caffeine elicits an anti-obesity effect in healthy, moderately obese subjects: a randomized double-blind placebo-controlled trial. *Nutrition Journal*, **15**(1):6.

Okada et al., 2012. Okada, Y., Kubo, M., Ohmiya, H., Takahashi, A., Kumasaka, N., Hosono, N., Maeda, S., Wen, W., Dorajoo, R., Go, M. J., *et al.*, 2012. Common variants at CDKAL1 and KLF9 are associated with body mass index in east Asian populations. *Nature Genetics*, **44**(3):302–306.

Padyukov et al., 2011. Padyukov, L., Seielstad, M., Ong, R. T. H., Ding, B., Ronnelid, J., Seddighzadeh, M., Alfredsson, L., and Klareskog, L., 2011. A genome-wide association study suggests contrasting associations in acpa-positive versus acpa-negative rheumatoid arthritis. *Annals of the Rheumatic Diseases*, **70**(2):259–265.

Qiu et al., 2014. Qiu, W., Rogers, A. J., Damask, A., Raby, B. a., Klanderman, B. J., Duan, Q. L., Tyagi, S., Niu, S., Anderson, C., Cahir-McFarland, E., *et al.*, 2014. Pharmacogenomics: Novel Loci Identification via Integrating Gene Differential Analysis and eQTL Analysis. *Human Molecular Genetics*, **23**(18):5017–5024.

Saxena et al., 2010. Saxena, R., Hivert, M. F., Langenberg, C., Tanaka, T., Pankow, J. S., Vollenweider, P., Lyssenko, V., Bouatia-Naji, N., Dupuis, J., Jackson, A. U., *et al.*, 2010. Genetic variation in GIPR influences the glucose and insulin responses to an oral glucose challenge. *Nature Genetics*, **42**(2):142–148.

Siddle et al., 2014. Siddle, K. J., Deschamps, M., Tailleux, L., Nédélec, Y., Pothlichet, J., Lugo-Villarino, G., Libri, V., Gicquel, B., Neyrolles, O., Laval, G., *et al.*, 2014. A genomic portrait of the genetic architecture and regulatory impact of microRNA expression in response to infection. *Genome Research*, **24**(5):850–9.

Smirnov et al., 2009. Smirnov, D. a., Morley, M., Shin, E., Spielman, R. S., and Cheung, V. G., 2009. Genetic analysis of radiation-induced changes in human gene expression. *Nature*, **459**(7246):587–591.

Speliotes et al., 2010. Speliotes, E. K., Willer, C. J., Berndt, S. I., Monda, K. L., Thorleifsson, G., Jackson, A. U., Allen, H. L., Lindgren, C. M., Luan, J., Magi, R., *et al.*, 2010. Association analyses of 249,796 individuals reveal 18 new loci associated with body mass index. *Nature Genetics*, **42**(11):937–948.

Storey, 2003. Storey, J. D., 2003. The positive false discovery rate: a Bayesian interpretation and the q -value. *The Annals of Statistics*, **31**(6):2013–2035.

The GTEx Consortium, 2015. The GTEx Consortium, 2015. The Genotype-Tissue Expression (GTEx) pilot analysis: Multitissue gene regulation in humans. *Science*, **348**(6235):648–660.

Trasande et al., 2013. Trasande, L., Sathyanarayana, S., Spanier, A. J., Trachtman, H., Attina, T. M., and Urbina, E. M., 2013. Urinary phthalates are associated with higher blood pressure in childhood. *Journal of Pediatrics*, **163**(3).

Van de Geijn et al., 2015. Van de Geijn, B., McVicker, G., Gilad, Y., and Pritchard, J. K., 2015. WASP: allele-specific software for robust molecular quantitative trait locus discovery. *Nature Methods*, **12**(11):1061–1063.

Wain et al., 2011. Wain, L. V., Verwoert, G. C., O'Reilly, P. F., Shi, G., Johnson, T., Johnson, A. D., Bochud, M., Rice, K. M., Henneman, P., Smith, A. V., *et al.*, 2011. Genome-wide association study identifies six new loci influencing pulse pressure and mean arterial pressure. *Nature Genetics*, **43**(10):1005–11.

Welter et al., 2014. Welter, D., MacArthur, J., Morales, J., Burdett, T., Hall, P., Junkins, H., Klemm, A., Flicek, P., Manolio, T., Hindorff, L., *et al.*, 2014. The NHGRI GWAS Catalog, a curated resource of SNP-trait associations. *Nucleic Acids Research*, **42**(D1).

Wen et al., 2012. Wen, W., Cho, Y.-S., Zheng, W., Dorajoo, R., Kato, N., Qi, L., Chen, C.-H., Delahanty, R. J., Okada, Y., Tabara, Y., *et al.*, 2012. Meta-analysis identifies common variants associated with body mass index in east Asians. *Nature Genetics*, **44**(3):307–11.

Wen et al., 2014. Wen, W., Zheng, W., Okada, Y., Takeuchi, F., Tabara, Y., Hwang, J.-Y., Dorajoo, R., Li, H., Tsai, F.-J., Yang, X., *et al.*, 2014. Meta-analysis of genome-wide association studies in East Asian-ancestry populations identifies four new loci for body mass index. *Human Molecular Genetics*, **23**(20):5492–504.

Wen and Stephens, 2014. Wen, X. and Stephens, M., 2014. Bayesian methods for genetic association analysis with heterogeneous subgroups: From meta-analyses to gene-environment interactions. *Annals of Applied Statistics*, **8**(1):176–203.

Yang et al., 2010. Yang, J., Benyamin, B., McEvoy, B. P., Gordon, S., Henders, A. K., and Others, 2010. Common {SNPs} explain a large proportion of the heritability for human height. *Nature Genetics*, **42**(7):565–569.

Yuan et al., 2008. Yuan, X., Waterworth, D., Perry, J. R. B., Lim, N., Song, K., Chambers, J. C., Zhang, W., Vollenweider, P., Stirnadel, H., Johnson, T., *et al.*, 2008. Population-Based Genome-wide Association Studies Reveal Six Loci Influencing Plasma Levels of Liver Enzymes. *American Journal of Human Genetics*, **83**(4):520–528.

Zhou, 2016. Zhou, X., 2016. A unified framework for variance component estimation with summary statistics in genome-wide association studies. *bioRxiv*, **10.1101/042846**.

Zhou and Stephens, 2012. Zhou, X. and Stephens, M., 2012. Genome-wide efficient mixed-model analysis for association studies. *Nature Genetics*, **44**(7):821–824.

Zunszain et al., 2011. Zunszain, P. A., Anacker, C., Cattaneo, A., Carvalho, L. A., and Pariante, C. M., 2011. Glucocorticoids, cytokines and brain abnormalities in depression. *Progress in Neuro-Psychopharmacology and Biological Psychiatry*, **35**(3):722–729.

Table S1: **Treatments used for analysis.** The control shown for each treatment is the vehicle used to dilute it. For example, dexamethasone was a powder resuspended in EtOH, so we used EtOH as control for the dexamethasone treatment. Note that we also matched the concentration of the vehicle used. In the case of all the treatments with EtOH as control, both the treatment and the control wells received 1ul of EtOH per 10,000μL of culturing media. "In range" denotes whether the treatment concentration matches physiological levels in the blood (within 10x), as indicated by Mayo Clinic or the Center for Disease Control. When this information was not available, we used literature reports and we indicate the relevant PMID.

Treatment ID	Common Name	Control	Treatment Concentration	Physiological Concentration
T1C1	Vitamin C	Media	$1.00 \times 10^{-5}M$	Yes
T2C1	Vitamin H	Media	$4.75 \times 10^{-10}M$	Yes
T3C1	Vitamin B3	Media	$1.50 \times 10^{-5}M$	Yes
T4C1	Vitamin B5	Media	$1.00 \times 10^{-7}M$	15539319
T5C1	Vitamin B6	Media	$1.00 \times 10^{-5}M$	Yes
T6C1	Vitamin A	Ethanol	$1.00 \times 10^{-8}M$	21307853
T7C1	Vitamin E	Ethanol	$5.00 \times 10^{-5}M$	Yes
T8C1	Vitamin K3	Ethanol	$1.00 \times 10^{-6}M$	21134493
T9C1	Aldosterone	Ethanol	$1.00 \times 10^{-5}M$	No (2,000x higher)
T10C1	Progesterone	Ethanol	$1.00 \times 10^{-5}M$	No (11x higher)
T10C2	Progesterone	DMSO	$1.00 \times 10^{-6}M$	Yes
T11C1	Estrogen	Ethanol	$1.00 \times 10^{-5}M$	No (1,000x higher)
T12C1	Dexamethasone	Ethanol	$1.00 \times 10^{-5}M$	21750684
T13C1	Caffeine	Media	$1.16 \times 10^{-3}M$	No (15x higher)
T14C1	Nicotine	Media	$6.16 \times 10^{-4}M$	12194923
T15C1	Copper	Media	$6.00 \times 10^{-5}M$	Yes
T16C1	Iron	Media	$5.00 \times 10^{-3}M$	18050301
T16C2	Iron	Media	$1.00 \times 10^{-5}M$	Yes
T17C1	Manganese	Media	$3.00 \times 10^{-3}M$	No (200,000x higher)
T17C2	Manganese	Media	$1.5 \times 10^{-8}M$	Yes
T18C1	Molybdenum	Media	$5.00 \times 10^{-4}M$	18050301
T19C1	Selenium	Media	$1.00 \times 10^{-5}M$	Yes
T20C1	Zinc	Media	$8.00 \times 10^{-5}M$	Yes
T21C1	Tunicamycin	DMSO	2 μg/mL	18704925
T22C1	PM 2.5	Media	5 μg/mL	23573366
T23C1	Vitamin D	Ethanol	$1.00 \times 10^{-7}M$	Yes
T24C1	Acrylamide	Media	2 ng/mL	Yes
T25C1	BP-3	Ethanol	1000 ng/mL	Yes
T26C1	BPA	Media	20 ng/mL	Yes
T27C1	Cadmium	Media	2 ng/mL	Yes
T28C1	CCl3	Media	0.100 ng/mL	Yes
T29C1	PFOA	Media	15 ng/mL	Yes
T30C1	Triclosan	Ethanol	1200 ng/mL	Yes
T32C1	Vitamin B9	Media	250 ng/mL	Yes
T33C1	Insulin	Media	1004.6 ng/mL	No (1000x higher)
T34C1	Glucagon	Media	0.650 ng/mL	Yes
T35C1	Oxytocin	Media	10.07 ng/mL	16778082
T36C1	Vasopressin	Media	0.0017 ng/mL	Yes
T37C1	Acetylcholine	Media	293.9 ng/mL	3801213
T38C1	Glucose	Media	1800 ng/mL	Yes
T39C1	MSG	Media	93 μg/mL	10644540
T40C1	BHA	Ethanol	0.2 μg/mL	Yes
T41C1	Ibuprofen	Ethanol	5 μg/mL	Yes
T42C1	Acetaminophen	Media	50 μg/mL	Yes
T43C1	Aspirin	Ethanol	10 μg/mL	Yes
T44C1	Loratadine	Ethanol	50 ng/mL	2965043
T45C1	Cetirizine	Media	10 ng/mL	15025737
T46C1	Dextromethorphan	Media	6 ng/mL	Yes
T47C1	Phthalate	Ethanol	100 ng/mL	Yes
T48C1	Cholesterol	Ethanol	2400 ng/mL	Yes

Table S2: **Summary of shallow sequencing.** Plate ID, Cell Type, Cell Line, Barcode ID (see Section 4), Treatment ID (see Table S1), Total reads (total number of reads sequenced), Aligned reads (number of reads after initial alignment), Quality reads (number of reads passing quality filtering), Clean reads (number of reads after duplicate removal).
See attached file, Supplemental_Table_S2.xlsx, also available at `http://genome.grid.wayne.edu/gxebrowser/Tables/Supplemental_Table_S2.xlsx`

Table S3: **Number of differentially expressed genes identified by shallow sequencing.** For each treatment, shown is the number of differentially expressed genes for each cell type at 10% BH-FDR, with an $|\text{logFC}| > 0.25$. Differentially expressed genes are determined by contrasting a treatment with its matched control.
See attached file, Supplemental_Table_S3.xlsx, also available at `http://genome.grid.wayne.edu/gxebrowser/Tables/Supplemental_Table_S3.xlsx`

Table S4: **Summary of deep sequencing.** Total reads, total number of reads sequenced. Aligned reads, number of reads after initial alignment. Quality reads, number of reads passing quality filtering. Clean reads, number of reads after allele-specific read mapping and duplicate removal via WASP.
See attached file, Supplemental_Table_S4.xlsx, also available at `http://genome.grid.wayne.edu/gxebrowser/Tables/Supplemental_Table_S4.xlsx`

Table S5: **Number of differentially expressed genes identified by deep sequencing.** For each treatment, shown is the number of genes tested (”All”), the number of differentially expressed genes in each cell type at 10% BH-FDR, with an $|\text{logFC}| > 0.25$, and the number of differentially expressed genes (DEG) both up- and downregulated relative to the control. DEG are determined by contrasting a treatment with its matched control. Included is also the number of DEG when contrasting Control 1 versus Control 2 in each plate separately.
See attached file, Supplemental_Table_S5.xlsx, also available at `http://genome.grid.wayne.edu/gxebrowser/Tables/Supplemental_Table_S5.xlsx`

Table S6: **Differentially expressed genes across treatments and cell lines.** For each cell type/treatment combination, given is the Ensembl transcript ID, DESeq2 p-value for differential expression, BH-adjusted p-value for differential expression, log_2 fold-change relative to the control, Ensembl gene ID, and Ensembl gene name.
See attached files, Supplemental_Table_S6.tar.gz, also available at `http://genome.grid.wayne.edu/gxebrowser/Tables/Supplemental_Table_S6.tar.gz`

Table S7: **Module assignments for network genes.**
See attached file, Supplemental_Table_S7.xlsx, also available at `http://genome.grid.wayne.edu/gxebrowser/Tables/Supplemental_Table_S7.xlsx`

Table S8: Z**-scores for module-treatment combinations.** For each module, listed is the t-score for the treatments in the cell type indicated. Also included for each module is the number of genes, the assigned treatment (based on the highest average t-score across cell types), the number of heterozygous and ASE SNPs in genes within the module, and the proportion of ASE to heterozygous SNPs in genes within the module.
See attached file, Supplemental_Table_S8.xlsx, also available at `http://genome.grid.wayne.edu/gxebrowser/Tables/Supplemental_Table_S8.xlsx`

Table S9: **Summary of ASE in deep sequenced samples.** For each treatment (row) and individual (columns), listed is the number of ASE SNPs at 10% FDR, with the total number of heterozygous SNPs tested in parentheses.

	**L CL**			**HUVEC**			**PBMC**			**Mel**			**SMC**		
	**18507**	**18508**	**19239**	**H004-A**	**H013-A**	**H288-L**	**KP39334**	**KP39346**	**KP39351**	**NM10-L**	**NM31-P**	**NM85-L**	**SM046-A**	**SM050-A**	**SM794-L**
**Media**	95 (10726)	94 (9355)	96 (9494)	95 (10536)	98 (10213)	115 (11330)	116 (10715)	128 (11943)	111 (10874)	90 (9325)	103 (10154)	112 (8520)	79 (7625)	85 (7725)	77 (7976)
**Ethanol**	107 (11874)	115 (12229)	118 (12474)	84 (10882)	89 (9667)	89 (10105)	125 (10145)	142 (12401)	108 (9785)	110 (9940)	113 (9884)	106 (8352)	90 (8369)	71 (8049)	66 (7805)
**DMSO**										31 (3364)	38 (3482)	39 (3120)			
**Dexamethasone**	17 (3561)	24 (3908)	48 (4062)	25 (3822)	34 (4500)	44 (4561)	42 (3531)	42 (4308)	42 (3774)	32 (3239)	27 (2653)	31 (2980)	38 (3655)	33 (3666)	56 (5609)
**Caffeine**	13 (3265)	19 (4225)	33 (3600)	21 (4460)	29 (4539)	29 (4887)	43 (3736)	31 (4034)	34 (4080)	21 (2506)	23 (2702)	27 (2718)	42 (3683)	29 (3542)	32 (3797)
**Nicotine**							25 (3524)	22 (3330)	35 (3522)						
**Copper**	32 (4153)	33 (3648)	21 (3807)				39 (3601)	28 (3954)	36 (3846)						
**Iron (C1)**							29 (3489)	29 (3379)	24 (3213)						
**Molybdenum**				36 (4440)	20 (2605)	28 (4407)	37 (3432)	30 (3316)	30 (3356)				40 (3937)	34 (3820)	73 (6650)
**Selenium**	12 (3154)	31 (3741)	26 (3273)	33 (5133)	38 (4580)	31 (4126)				11 (1872)	15 (2370)	39 (3313)	49 (3986)	34 (4229)	34 (3585)
**Zinc**							34 (3228)	34 (3950)	41 (4042)						
**Tunicamycin**										15 (2336)	20 (2813)	39 (3792)			
**Vitamin D**				31 (4196)	29 (4533)	32 (4610)	13 (2222)	21 (3170)	7 (1345)	20 (3120)	25 (3744)	49 (3572)	39 (3405)	31 (3659)	31 (4022)
**Acrylamide**	32 (4047)	57 (4781)	40 (3611)	63 (5681)	79 (6535)	68 (6829)							42 (4629)	44 (4410)	43 (4983)
**BP-3**				48 (4941)	54 (5030)	34 (4529)							54 (4769)	33 (4001)	33 (4107)
**BPA**	32 (3631)	37 (3584)	31 (3336)										45 (4592)	40 (4154)	37 (3627)
**Cadmium**	20 (2630)	26 (3524)	29 (4081)				32 (3746)	43 (4303)	44 (3988)						
**PFOA**	23 (3752)	28 (3663)	31 (3670)										50 (4989)	21 (3015)	35 (4305)
**Vitamin H**							40 (3390)	32 (3786)	37 (3560)						
**Triclosan**				38 (4430)	64 (6267)	35 (4224)				38 (4756)	37 (4411)	38 (3726)	37 (3882)	51 (4251)	44 (4820)
**Insulin**				38 (4257)	32 (4097)	33 (4063)				34 (4582)	40 (4369)	50 (4866)	48 (4681)	40 (4113)	41 (4745)
**Acetyl Choline**							31 (4337)	24 (3175)	37 (3583)						
**Vitamin B3**							41 (3361)	23 (2603)	38 (3840)						
**BHA**				34 (4278)	65 (6326)	35 (4605)	27 (2605)	31 (3221)	24 (2648)	31 (3903)	50 (4627)	41 (4393)			
**Ibuprofen**										40 (4566)	46 (4716)	43 (4293)			
**Acetaminophen**							27 (3793)	29 (3418)	40 (3793)	30 (3689)	31 (4389)	55 (4651)	59 (5735)	38 (4252)	37 (4240)
**Aspirin**	28 (3484)	26 (2855)	38 (3627)	41 (4064)	54 (4853)	42 (4967)	27 (3224)	38 (3778)	33 (3254)	32 (4142)	60 (5924)	35 (2585)	37 (4486)	43 (4222)	41 (3996)
**Loratadine**	30 (3739)	41 (3404)	50 (4981)							39 (4357)	33 (4610)	41 (3858)			
**Cetirizine**	24 (3826)	20 (2151)	32 (3548)												
**Phthalate**	27 (3698)	28 (3432)	31 (3695)	30 (4118)	52 (4872)	36 (4485)	24 (3248)	26 (3184)	42 (4117)	23 (3474)	38 (4756)	53 (4754)			
**Vitamin B5**							41 (3513)	28 (3767)	33 (3402)				28 (2510)	32 (3628)	33 (4025)
**Vitamin B6**							47 (3386)	31 (4153)	27 (3297)				25 (2960)	35 (3597)	40 (4021)
**Vitamin A**	27 (3885)	28 (4636)	34 (4458)	11 (2420)	38 (4649)	34 (4591)	50 (3980)	26 (3531)	29 (3590)	28 (2820)	16 (2454)	41 (3500)	35 (3589)	37 (3343)	44 (4565)
**Vitamin E**							45 (3691)	35 (3565)	24 (2818)	28 (3124)	25 (3336)	33 (3147)			
**Aldosterone**													45 (4517)	36 (3403)	38 (4005)

Table S10: **ASE results for all SNPs tested.** All heterozygous SNPs covered by at least 40 reads are listed, along with the cell type and treatment condition. Also listed are the number of reads covering the reference and alternate alleles, the ASE $\hat{\beta}$, standard error, p-value from QuASAR, and the q-value.
See attached file, Supplemental_Table_S10.txt.gz, also available at `http://genome.grid.wayne.edu/gxebrowser/Tables/Supplemental_Table_S10.txt.gz`

Table S11: **SNPs displaying conditional ASE.** For each cASE SNP, listed is the SNP rsID, cell type, gene, treatment, the ΔAST Z-score, empirical p-value, and q-value, the MeSH control-only BF, and the MeSH treatment-only BF. Significant cASE is defined as having q-value < 0.24 (24% FDR) from at least one of the tests.

rsID	Cell Type	Gene	Treatment	$Z_{\triangle}$	$Z_{\triangle}$ corrected p-value	$Z_{\triangle}$ q-value	MeSH C$_{BF}$	MeSH T$_{BF}$
rs9069	LCL	*RAB4A*	Selenium	5.547	2.91E-08	0.091	-5.931	-2.961
rs16926021	LCL	*KIAA1279*	Copper	-4.405	1.06E-05	0.249	-0.286	3.500
rs4980895	LCL	*CCDC77*	Dexamethasone	-4.653	3.27E-06	0.224	3.286	0.264
rs17521586	LCL	*AKAP11*	Caffeine	-5.120	3.06E-07	0.091	0.492	4.635
rs11699	LCL	*TMCO3*	Selenium	-5.260	1.44E-07	0.091	-0.426	2.063
rs17103743	LCL	*KHNYN*	Vitamin A	-5.142	2.72E-07	0.091	2.829	-5.724
rs11620816	LCL	*HECTD1*	Caffeine	-4.553	5.28E-06	0.224	-0.325	4.071
rs7167612	LCL	*TRIP4*	Selenium	5.478	4.30E-08	0.091	0.366	4.345
rs12929100	LCL	*SMG1*	Selenium	-4.637	3.53E-06	0.224	2.144	2.188
rs1801635	LCL	*PITPNA*	Caffeine	5.265	1.40E-07	0.091	0.297	3.806
rs77903511	LCL	*AC087645*.1	Caffeine	-5.182	2.19E-07	0.091	-3.889	-11.855
rs9303891	LCL	*USP14*	Selenium	-5.351	8.75E-08	0.091	-0.619	2.195
rs1055002	LCL	*VPS4B*	Selenium	-4.377	1.20E-05	0.249	-0.568	2.680
rs17072146	LCL	*SERPINB10*	Selenium	4.417	1.00E-05	0.249	0.644	3.081
rs7247504	LCL	*STAP2*	Caffeine	4.915	8.89E-07	0.091	1.447	2.959
rs17028275	LCL	*VPS54*	Caffeine	-6.113	9.77E-10	0.091	-0.616	3.111
rs77388906	LCL	*C1D*	Vitamin A	-4.403	1.07E-05	0.249	-7.380	-27.925
rs1878583	LCL	*SLC25A12*	Copper	4.435	9.21E-06	0.244	1.981	2.114
rs7587	LCL	*SAMM50*	Copper	-4.705	2.54E-06	0.208	-0.486	4.110
rs3773713	LCL	*SSR3*	Caffeine	5.934	2.96E-09	0.091	-18.250	-9.325
rs16833703	LCL	*LAMP3*	Selenium	4.411	1.03E-05	0.249	1.505	1.909
rs693758	LCL	*PHACTR1*	Dexamethasone	-4.498	6.87E-06	0.224	1.240	2.609
rs2269978	LCL	*SCRN1*	Caffeine	-4.414	1.02E-05	0.249	0.117	3.810
rs1043615	LCL	*DNAJB9*	Vitamin A	-4.612	3.98E-06	0.224	2.365	2.066
rs16904746	LCL	*PHF20L1*	Copper	-4.956	7.19E-07	0.091	-0.083	3.917
rs10964471	LCL	*SMARCA2*	Copper	5.560	2.69E-08	0.091	0.292	4.658
rs1053959	LCL	*PTGR1*	Selenium	5.073	3.92E-07	0.091	-11.511	3.055
rs1053959	LCL	*PTGR1*	Caffeine	4.970	6.71E-07	0.091	-8.299	2.507
rs1065675	SMC	*NUP133*	Triclosan	-4.599	4.25E-06	0.224	2.441	2.080
rs1829556	SMC	*WNT5A*	PFOA	4.428	9.53E-06	0.249	1.070	3.003
rs2274136	SMC	*NUP153*	BPA	4.495	6.94E-06	0.224	2.860	1.576
rs945508	Mel	*ARHGEF11*	Tunicamycin	-4.696	2.65E-06	0.208	1.599	3.019
rs2277300	Mel	*OSBPL5*	Vitamin D	-4.466	7.99E-06	0.224	1.442	2.584
rs3742722	Mel	*CEP128*	Selenium	-5.471	4.49E-08	0.091	2.506	3.707
rs3815820	Mel	*BANP*	Tunicamycin	-4.440	8.99E-06	0.244	1.969	2.349
rs3088016	Mel	*CCDC137*	Dexamethasone	-4.397	1.10E-05	0.249	-0.644	3.293
rs112976362	Mel	*ADNP2*	Vitamin D	4.483	7.35E-06	0.224	1.640	2.505
rs2229259	Mel	*ECH1*	Tunicamycin	-4.901	9.53E-07	0.091	1.624	3.654
rs2304802	Mel	*TMA16*	Tunicamycin	-5.045	4.53E-07	0.091	-0.357	4.635
rs17482078	Mel	*ERAP1*	Selenium	-4.402	1.07E-05	0.249	1.739	2.347
rs221790	Mel	*GIGYF1*	Vitamin E	4.704	2.55E-06	0.208	-2.867	3.750
rs1127635	Mel	*STXBP3*	BHA	4.405	1.06E-05	0.249	-0.221	3.242
rs823137	Mel	*RAB7L1*	Acetaminophen	4.462	8.13E-06	0.244	1.239	-6.282
rs8946	Mel	*BAG3*	Phthalate	4.997	5.83E-07	0.091	-8.535	-8.497
rs11025310	Mel	*NAV2*	Loratadine	5.947	2.73E-09	0.091	0.877	5.392
rs35216474	Mel	*PAMR1*	Aspirin	-5.298	1.17E-07	0.091	0.711	-10.708
rs4752904	Mel	*PTPRJ*	Acetaminophen	-5.490	4.02E-08	0.091	0.469	4.987
rs4414223	Mel	*SNX19*	Acetaminophen	5.014	5.34E-07	0.091	1.417	3.583
rs10844253	Mel	*FGD4*	Acetaminophen	4.391	1.13E-05	0.249	2.708	1.553
rs1298463	Mel	*ZFC3H1*	Acetaminophen	4.775	1.80E-06	0.208	3.790	-0.088
rs177393	Mel	*PAPLN*	Ibuprofen	-4.454	8.44E-06	0.244	3.397	-0.870
rs903160	Mel	*SMG6*	Aspirin	4.700	2.61E-06	0.208	3.350	0.695
rs1071705	Mel	*NUP88*	Ibuprofen	-4.636	3.56E-06	0.224	4.073	-0.987
rs2279103	Mel	*CTDP1*	Loratadine	-5.204	1.95E-07	0.091	1.590	3.739
rs56076827	Mel	*IFT172*	Phthalate	4.435	9.22E-06	0.244	-1.399	3.427
rs2285365	Mel	*PLXND1*	Loratadine	4.507	6.58E-06	0.224	2.852	-3.341
rs1048145	Mel	*NCK1*	Phthalate	4.409	1.04E-05	0.249	0.096	3.288
rs79940815	Mel	*AC024560*.3	Phthalate	4.679	2.88E-06	0.224	-3.378	3.628
rs11762014	Mel	*TECPR1*	BHA	5.211	1.88E-07	0.091	0.267	4.214
rs13277646	Mel	*UBXN2B*	BHA	-4.614	3.96E-06	0.224	2.073	2.706
rs8507	Mel	*ZER1*	Aspirin	-4.497	6.89E-06	0.224	0.892	3.090
rs6661946	HUVEC	*HEATR1*	Vitamin A	4.501	6.78E-06	0.224	-0.012	3.166
rs3209896	HUVEC	*AKR1C3*	Selenium	-5.405	6.49E-08	0.091	2.526	-9.749
rs11552445	HUVEC	*COMMD3-BMI1*	Caffeine	-4.508	6.53E-06	0.224	-1.210	4.013
rs11556066	HUVEC	*TWF1*	Caffeine	-4.800	1.59E-06	0.177	-2.950	4.085
rs6580942	HUVEC	*ESPL1*	Dexamethasone	5.179	2.23E-07	0.091	0.632	3.903
rs61730727	HUVEC	*PWP1*	Caffeine	4.094	4.24E-05	0.445	-0.115	3.431
rs1168666	HUVEC	*SETD1B*	Dexamethasone	5.503	3.73E-08	0.091	3.709	1.691
rs1168666	HUVEC	*SETD1B*	Vitamin A	5.398	6.74E-08	0.091	3.576	1.447
rs6488868	HUVEC	*SBNO1*	Molybdenum	-4.332	1.48E-05	0.281	-0.267	3.479
rs15587	HUVEC	*DDX55*	Selenium	-5.255	1.48E-07	0.091	1.944	3.490
rs872224	HUVEC	*NCOR2*	Dexamethasone	-4.413	1.02E-05	0.249	1.389	2.630
rs1372085	HUVEC	*PARP4*	Vitamin A	-4.456	8.33E-06	0.244	-0.136	3.608
rs1822135	HUVEC	*PARP4*	Vitamin A	-5.584	2.35E-08	0.091	3.272	2.641
rs14193	HUVEC	*RABGGTA*	Selenium	-4.918	8.75E-07	0.091	2.234	2.632
rs14193	HUVEC	*RABGGTA*	Caffeine	-4.654	3.25E-06	0.224	2.190	2.126
rs9904043	HUVEC	*SUPT6H*	Selenium	4.739	2.14E-06	0.208	2.262	3.068
rs1046045	HUVEC	*YPEL2*	Dexamethasone	-4.390	1.13E-05	0.249	1.953	1.903
rs4298	HUVEC	*ACE*	Selenium	4.704	2.55E-06	0.208	3.815	-8.462
rs7503278	HUVEC	*ACTG1*	Dexamethasone	5.424	5.83E-08	0.091	-11.665	5.528
rs6102	HUVEC	*SERPINB10*	Dexamethasone	-6.171	6.80E-10	0.091	-9.610	-1.867
rs2043449	HUVEC	*CYP20A1*	Vitamin D	4.797	1.61E-06	0.177	1.463	3.039
rs2255341	HUVEC	*ATP9A*	Dexamethasone	-4.511	6.44E-06	0.224	0.860	2.966
rs1135618	HUVEC	*MRPL39*	Molybdenum	4.712	2.45E-06	0.208	3.155	1.214

Continued on next page...

Table S11 – continued from previous page

rsID	Cell Type	Gene	Treatment	$Z_{\triangle}$	$Z_{\triangle}$ corrected p-value	$Z_{\triangle}$ q-value	MeSH C_{BF}	MeSH T_{BF}
rs2272007	HUVEC	*ULK4*	Caffeine	4.419	9.93E-06	0.249	-5.232	-0.387
rs17052357	HUVEC	*PBRM1*	Selenium	4.481	7.43E-06	0.224	0.943	3.500
rs13080329	HUVEC	*COL8A1*	Caffeine	5.452	4.99E-08	0.091	-5.673	-1.557
rs2270226	HUVEC	*RBPJ*	Vitamin D	-4.482	7.38E-06	0.224	1.449	2.678
rs190450	HUVEC	*FBN2*	Vitamin A	4.455	8.41E-06	0.244	0.975	3.210
rs2502599	HUVEC	*SYNJ2*	Vitamin A	5.852	4.87E-09	0.091	-12.583	-7.599
rs2502598	HUVEC	*SYNJ2*	Vitamin A	5.780	7.47E-09	0.091	-8.376	-4.665
rs13272579	HUVEC	*PREX2*	Molybdenum	-4.484	7.33E-06	0.224	0.264	3.203
rs896849	HUVEC	*TP53INP1*	Vitamin D	4.446	8.76E-06	0.244	-0.431	3.818
rs8218	HUVEC	*HRSP12*	Vitamin D	4.646	3.38E-06	0.224	2.998	0.787
rs1129768	HUVEC	*EHMT1*	Selenium	-4.989	6.08E-07	0.091	-0.311	4.257
rs2296377	PBMC	*TOR3A*	Acetylcholine	-4.408	1.04E-05	0.249	-2.439	3.114
rs12084264	PBMC	*C1orf27*	BHA	4.580	4.65E-06	0.224	1.509	2.872
rs7899928	PBMC	*WDR11*	Phthalate	-4.686	2.79E-06	0.224	-3.065	4.014
rs1145207	PBMC	*SIK3*	Cadmium	4.552	5.31E-06	0.224	3.177	0.600
rs17125548	PBMC	*SORL1*	Acetylcholine	-4.503	6.70E-06	0.224	-0.488	2.832
rs3168600	PBMC	*COPS7A*	Phthalate	5.342	9.19E-08	0.091	1.215	4.022
rs15993	PBMC	*ACTN1*	Acetaminophen	-4.381	1.18E-05	0.249	0.202	3.472
rs2526882	PBMC	*PCNX*	Aspirin	4.864	1.15E-06	0.091	3.686	0.722
rs11160859	PBMC	*IGHG2*	Acetylcholine	4.528	5.94E-06	0.224	-10.775	-2.669
rs11651270	PBMC	*NLRP1*	Phthalate	-4.491	7.08E-06	0.224	1.896	1.992
rs3826709	PBMC	*KRI1*	Acetylcholine	-4.413	1.02E-05	0.249	0.212	3.479
rs11555053	PBMC	*SUGP1*	Aspirin	5.113	3.18E-07	0.091	-1.542	4.131
rs1130426	PBMC	*ETFB*	Aspirin	-4.876	1.08E-06	0.091	-0.667	3.979
rs1138484	PBMC	*ST3GAL5*	Phthalate	5.294	1.20E-07	0.091	0.729	3.877
rs62154801	PBMC	*ANKRD36*	Acetylcholine	4.761	1.93E-06	0.208	1.934	-7.373
rs3951216	PBMC	*LY75-CD302*	BHA	-4.435	9.22E-06	0.244	2.713	1.615
rs463312	PBMC	*TUBB1*	Acetaminophen	-4.645	3.40E-06	0.224	-3.572	2.631
rs415064	PBMC	*TUBB1*	Acetaminophen	-4.645	3.40E-06	0.224	-3.572	2.631
rs394321	PBMC	*IGLV3-1*	Cadmium	7.423	1.15E-13	0.091	4.393	6.018
rs2034244	PBMC	*MIER3*	Acetylcholine	6.542	6.09E-11	0.091	3.350	5.263
rs3097146	PBMC	*TMEM161B*	BHA	-4.702	2.58E-06	0.208	4.057	-0.886
rs1131769	PBMC	*TMEM173*	Acetylcholine	4.482	7.39E-06	0.224	0.030	3.709
rs78233829	PBMC	*TMEM173*	Acetylcholine	4.531	5.86E-06	0.224	0.092	3.776
rs1801265	PBMC	*DPYD*	Nicotine	4.686	2.79E-06	0.224	1.769	2.680
rs200319336	PBMC	*NBPF9*	Vitamin H	-5.600	2.15E-08	0.091	-13.145	-10.942
rs200319336	PBMC	*NBPF9*	Molybdenum	-5.984	2.18E-09	0.091	-9.305	-9.975
rs200319336	PBMC	*NBPF9*	Caffeine	-5.621	1.90E-08	0.091	-10.205	-10.707
rs200319336	PBMC	*NBPF9*	Copper	-5.579	2.42E-08	0.091	-9.740	-10.744
rs1778112	PBMC	*PDE4DIP*	Vitamin D	-5.642	1.68E-08	0.091	6.248	-8.641
rs6029	PBMC	*F5*	Copper	-4.879	1.06E-06	0.091	-1.174	4.244
rs7998427	PBMC	*SETDB2*	Vitamin A	-5.443	5.25E-08	0.091	3.535	2.027
rs17103743	PBMC	*KHNYN*	Vitamin B5	-4.934	8.06E-07	0.091	1.727	-9.641
rs976272	PBMC	*SLC38A6*	Zinc	4.724	2.32E-06	0.208	1.642	2.627
rs12971834	PBMC	*CTD-2521M24.9*	Vitamin B6	4.537	5.70E-06	0.224	-1.040	3.829
rs12979773	PBMC	*CTD-2521M24.9*	Vitamin B6	4.332	1.48E-05	0.281	-0.856	3.675
rs2627765	PBMC	*PNPT1*	Vitamin B5	4.565	5.00E-06	0.224	-2.171	4.608
rs1107065	PBMC	*DIP2A*	Caffeine	5.232	1.68E-07	0.091	-0.594	4.504
rs1051169	PBMC	*S100B*	Caffeine	6.039	1.55E-09	0.091	6.739	-9.889
rs1051169	PBMC	*S100B*	Vitamin D	9.170	4.74E-20	0.091	13.692	-1.973
rs9610729	PBMC	*TOP3B*	Vitamin H	5.366	8.04E-08	0.091	1.196	4.206
rs2280083	PBMC	*CHST2*	Dexamethasone	4.199	2.68E-05	0.314	-1.052	3.675
rs6768054	PBMC	*RNF13*	Vitamin A	5.040	4.67E-07	0.091	1.532	3.809
rs1042994	PBMC	*PLK2*	Vitamin D	-4.929	8.27E-07	0.091	4.180	-2.589
rs7735338	PBMC	*CWC27*	Molybdenum	4.457	8.32E-06	0.244	1.801	2.558
rs34741656	PBMC	*STEAP4*	Vitamin B5	-5.237	1.63E-07	0.091	-10.625	-1.276
rs3118863	PBMC	*DAPK1*	Nicotine	4.628	3.69E-06	0.224	3.755	-7.202
rs270502	LCL	*TARBP1*	Cadmium	4.570	4.88E-06	0.224	4.127	-4.000
rs8018720	LCL	*SEC23A*	Cadmium	4.179	2.92E-05	0.323	-0.083	3.495
rs1107413	LCL	*SRPRB*	PFOA	4.229	2.35E-05	0.294	-2.725	3.671
rs34558821	LCL	*FRYL*	Cetirizine	-4.439	9.05E-06	0.244	-1.760	3.376
rs200499	LCL	*HIST1H4J*	Phthalate	-6.368	1.91E-10	0.091	-6.961	1.004
rs73581683	LCL	*HDDC2*	Cadmium	4.793	1.65E-06	0.208	-10.409	-3.196
rs4310250	LCL	*PRRC2B*	Cadmium	-4.467	7.95E-06	0.224	0.757	3.413
rs7868455	LCL	*EHMT1*	Cetirizine	4.925	8.42E-07	0.091	-11.233	1.298
rs1126972	HUVEC	*PPT1*	Phthalate	-4.518	6.24E-06	0.224	-2.275	-16.018
rs2794751	HUVEC	*HEATR1*	BHA	-4.795	1.63E-06	0.208	3.104	2.032
rs35363135	HUVEC	*RPS6KB2*	Triclosan	-4.751	2.03E-06	0.208	2.565	2.308
rs13508	HUVEC	*MRPS31*	Insulin	-4.677	2.91E-06	0.224	3.086	1.426
rs61620792	HUVEC	*RP11-632K20.7*	Insulin	-4.753	2.00E-06	0.208	-6.399	0.725
rs4777755	HUVEC	*CHD2*	Phthalate	-4.397	1.10E-05	0.249	2.340	2.001
rs189899	HUVEC	*GOSR2*	Phthalate	4.505	6.63E-06	0.224	-2.741	3.651
rs17853713	HUVEC	*RNF213*	Insulin	4.852	1.22E-06	0.091	1.889	3.422
rs7238987	HUVEC	*CYB5A*	Aspirin	-4.576	4.75E-06	0.224	1.047	-6.300
rs1135618	HUVEC	*MRPL39*	Aspirin	-4.781	1.74E-06	0.208	-0.658	4.449
rs2470548	HUVEC	*ANKRD28*	Triclosan	-5.902	3.60E-09	0.091	4.837	2.460
rs9503797	HUVEC	*RP1-140K8.5*	Phthalate	-4.478	7.55E-06	0.224	-10.770	-1.455
rs12540098	HUVEC	*MICALL2*	Triclosan	4.463	8.09E-06	0.244	1.631	2.531
rs16836943	SMC	*RPRD2*	Dexamethasone	5.434	5.51E-08	0.091	3.870	2.379
rs1045247	SMC	*CDC42BPA*	Vitamin D	4.578	4.68E-06	0.224	0.692	3.369
rs6989	SMC	*LGALS8*	Vitamin B5	4.901	9.56E-07	0.091	2.584	2.697
rs2025258	SMC	*IPO4*	Aldosterone	4.728	2.26E-06	0.208	3.005	1.841
rs13225	SMC	*AKAP13*	Caffeine	4.392	1.13E-05	0.249	2.347	1.843
rs11640454	SMC	*KNOP1*	Dexamethasone	4.283	1.85E-05	0.294	-0.580	3.503
rs2251219	SMC	*SMIM4*	Dexamethasone	-4.954	7.26E-07	0.091	3.228	1.675
rs13146	SMC	*UMPS*	Vitamin B5	4.580	4.66E-06	0.224	0.998	3.346
rs6778479	SMC	*WWTR1*	Molybdenum	4.705	2.54E-06	0.208	1.650	3.251
rs1042094	SMC	*PPP3CA*	Vitamin B5	4.416	1.01E-05	0.249	2.793	1.339
rs1042094	SMC	*PPP3CA*	Caffeine	4.691	2.72E-06	0.208	2.770	1.787
rs698912	SMC	*COL4A3BP*	Vitamin B5	-4.662	3.13E-06	0.224	1.348	3.094
rs2278221	SMC	*ADAMTS2*	Selenium	-4.593	4.36E-06	0.224	-1.613	3.841

Continued on next page...

Table S11 – continued from previous page

rsID	Cell Type	Gene	Treatment	$Z_\triangle$	$Z_\triangle$ corrected p-value	$Z_\triangle$ q-value	MeSH C_{BF}	MeSH T_{BF}
rs3747807	SMC	*DMTF1*	Caffeine	4.457	8.32E-06	0.244	2.008	2.464
rs1061195	SMC	*SLC25A32*	Selenium	4.455	8.39E-06	0.244	2.653	1.486
rs3206852	SMC	*FOCAD*	Vitamin D	-4.658	3.19E-06	0.224	-2.092	3.745

Table S12: **cASE results for all SNPs tested.** For each SNP tested for cASE, listed is the same information in S11, as well as ASE data (similar to Table S10) for treatment and control SNPs, and two additional columns: the SNP-based expression and SNP-based log(fold change), described in Section 9.3.
See attached file, Supplemental_Table_S12.txt.gz, also available at `http://genome.grid.wayne.edu/gxebrowser/Tables/Supplemental_Table_S12.txt.gz`

Table S13: **cASE genes with a reQTL signal.** Out of 83 genes reported to have GxE in previous reQTL and ASE studies and able to be tested for cASE in our data, 63 genes are listed in this table that also have cASE in our data (p-value <0.05). The table denotes the genes, the environment in which the reQTL was identified, the citation of the paper establishing the reQTL, and the cell types and treatments for which we find cASE

Gene with reQTL	Environment for reQTL / ASE	Citation	Cell Type(s) for cASE (p-value <0.05)	Treatment(s) for cASE
ADCY3	Rhinovirus	Caliskan M et al., 2015 (25874939)	HUVEC, LCL, Mel, PBMC	BHA, Caffeine, Ibuprofen, Loratadine, Mono-n-butyl Phthalate, Retinoic Acid, Vitamin B6
AGTRAP	Rhinovirus	Caliskan M et al., 2015 (25874939)	HUVEC	Insulin
ARL5B	Rhinovirus	Caliskan M et al., 2015 (25874939)	HUVEC, LCL, PBMC, SMC	Acetylcholine, Acrylamide, BHA, Bisphenol A, Caffeine, Copper, Dexamethasone, Iron, Molybdenum, Panthothenic acid, Retinoic Acid, Selenium, Vitamin B6, Vitamin D, Vitamin H
CALM1	Rhinovirus	Caliskan M et al., 2015 (25874939)	HUVEC, LCL, Mel	Aspirin, Cadmium, Caffeine
CCDC146	Rhinovirus	Caliskan M et al., 2015 (25874939)	PBMC	Acetaminophen
DNTTIP1	Rhinovirus	Caliskan M et al., 2015 (25874939)	Mel, PBMC	Acetaminophen, Caffeine, Insulin, Tunicamycin, Vitamin E, Zinc
EXOSC9	Rhinovirus	Caliskan M et al., 2015 (25874939)	HUVEC, LCL, SMC	Dexamethasone, Molybdenum, Retinoic Acid
FBN2	Rhinovirus	Caliskan M et al., 2015 (25874939)	HUVEC, SMC	Benzophenone-3, Bisphenol A, Caffeine, Retinoic Acid, Selenium, Vitamin B6, Vitamin D
GJA3	Rhinovirus	Caliskan M et al., 2015 (25874939)	Mel	Loratadine
INPP1	Rhinovirus	Caliskan M et al., 2015 (25874939)	HUVEC, LCL, Mel, PBMC, SMC	Acetaminophen, Acrylamide, Aspirin, Benzophenone-3, BHA, Bisphenol A, Copper, Insulin, Mono-n-butyl Phthalate, Retinoic Acid, Selenium, Vitamin D
IRF5	Rhinovirus	Caliskan M et al., 2015 (25874939)	PBMC	Mono-n-butyl Phthalate, Panthothenic acid
ITGA2	Rhinovirus	Caliskan M et al., 2015 (25874939)	HUVEC, Mel, SMC	Aspirin, Benzophenone-3, Caffeine, Dexamethasone, Ibuprofen, Insulin, Loratadine, Molybdenum, Mono-n-butyl Phthalate, Panthothenic acid, Retinoic Acid, Selenium, Triclosan, Vitamin D
MASTL	Rhinovirus	Caliskan M et al., 2015 (25874939)	LCL, Mel, PBMC	Aspirin, BHA, Caffeine, Cetirizine, Copper, Mono-n-butyl Phthalate, Retinoic Acid
MYO1D	Rhinovirus	Caliskan M et al., 2015 (25874939)	Mel	Caffeine
RAB31	Rhinovirus	Caliskan M et al., 2015 (25874939)	HUVEC, Mel, SMC	Acetaminophen, Aspirin, Dexamethasone, Ibuprofen, Insulin, Selenium, Vitamin D
SLFN5	Rhinovirus	Caliskan M et al., 2015 (25874939)	LCL, Mel, PBMC	Acetaminophen, Cadmium, Caffeine, Copper, Dexamethasone, Loratadine, Mono-n-butyl Phthalate, Panthothenic acid, Retinoic Acid, Tunicamycin, Vitamin D, Vitamin E
SPTLC2	Rhinovirus	Caliskan M et al., 2015 (25874939)	HUVEC, LCL, Mel, PBMC, SMC	Acetylcholine, Acrylamide, Aspirin, Benzophenone-3, Caffeine, Copper, Dexamethasone, Ibuprofen, Iron, Loratadine, Molybdenum, Mono-n-butyl Phthalate, Retinoic Acid, Selenium, Vitamin B6, Vitamin D
TMTC1	Rhinovirus	Caliskan M et al., 2015 (25874939)	Mel	Aspirin, Mono-n-butyl Phthalate
C1orf85	Flu Vaccine	Franco LM et al., 2013 (23878721)	HUVEC, LCL, Mel, SMC	Acrylamide, Caffeine, Insulin, Molybdenum, Panthothenic acid, Perfluorooctanoic Acid, Tunicamycin
DIP2A	Flu Vaccine	Franco LM et al., 2013 (23878721)	LCL, Mel, PBMC	Caffeine, Insulin, Loratadine, Nicotine, Panthothenic acid, Retinoic Acid, Selenium, Triclosan, Tunicamycin, Vitamin B3, Vitamin D, Vitamin E, Vitamin H, Zinc
DYNLT1	Flu Vaccine	Franco LM et al., 2013 (23878721)	HUVEC, Mel	Retinoic Acid, Triclosan, Vitamin D, Vitamin E
FGD2	Flu Vaccine	Franco LM et al., 2013 (23878721)	LCL, PBMC	Acetaminophen, Aspirin, Caffeine, Copper, Mono-n-butyl Phthalate, Retinoic Acid, Vitamin E
GM2A	Flu Vaccine	Franco LM et al., 2013 (23878721)	HUVEC, LCL, Mel, PBMC, SMC	Acrylamide, Aspirin, Benzophenone-3, BHA, Caffeine, Copper, Dexamethasone, Loratadine, Molybdenum, Nicotine, Panthothenic acid, Retinoic Acid, Selenium, Triclosan, Tunicamycin, Vitamin B3, Vitamin B6, Vitamin D, Zinc
HERC2	Flu Vaccine	Franco LM et al., 2013 (23878721)	LCL, Mel, SMC	Acetaminophen, Caffeine, Dexamethasone, Perfluorooctanoic Acid, Retinoic Acid, Tunicamycin
JUP	Flu Vaccine	Franco LM et al., 2013 (23878721)	SMC	Acrylamide
RPL14	Flu Vaccine	Franco LM et al., 2013 (23878721)	SMC	Aspirin, Triclosan, Vitamin D
TAP2	Flu Vaccine	Franco LM et al., 2013 (23878721)	HUVEC, LCL, Mel	Acetaminophen, Acrylamide, Aspirin, BHA, Bisphenol A, Caffeine, Copper, Ibuprofen, Loratadine

Continued on next page...

Table S13 – continued from previous page				
**Gene with reQTL**	**Environment for reQTL**	**Citation**	**Cell Type(s) for cASE (p-value <0.05)**	**Treatment(s) for cASE**
PRUNE2	Malaria	Idaghdour Y et al., 2012 (22949651)	Mel	Aspirin, Dexamethasone, Insulin, Tunicamycin, Vitamin E
SLC39A8	Malaria	Idaghdour Y et al., 2012 (22949651)	LCL, PBMC	Acrylamide, Copper, Dexamethasone, Perfluorooctanoic Acid, Selenium
UNC119B	Malaria	Idaghdour Y et al., 2012 (22949651)	HUVEC, LCL, PBMC, SMC	Acrylamide, Caffeine, Cetirizine, Loratadine, Nicotine, Panthothenic acid, Retinoic Acid, Triclosan, Vitamin D
ARHGEF18	Opiates	Knowles DA et al., 2015 (13/025874)	HUVEC, LCL, Mel, PBMC	Acrylamide, Dexamethasone, Ibuprofen, Mono-n-butyl Phthalate, Retinoic Acid
CPT1B	Blood Pressure Medication	Knowles DA et al., 2015 (13/025874)	LCL, Mel, PBMC	Acetaminophen, Cadmium, Caffeine, Ibuprofen, Mono-n-butyl Phthalate
DYSF	Exercise	Knowles DA et al., 2015 (13/025874)	HUVEC	Benzophenone-3, Dexamethasone, Molybdenum, Retinoic Acid
GSTO1	BMI	Knowles DA et al., 2015 (13/025874)	HUVEC, LCL, PBMC	Aspirin, Caffeine, Mono-n-butyl Phthalate, Vitamin D
IFI44L	Exercise	Knowles DA et al., 2015 (13/025874)	LCL, PBMC	Aspirin, Cadmium, Iron, Molybdenum, Vitamin B6, Vitamin D
IL10RA	Num. of Cigarettes/day	Knowles DA et al., 2015 (13/025874)	LCL, PBMC	Aspirin, Copper
NKG7	Antidepressants	Knowles DA et al., 2015 (13/025874)	PBMC	Vitamin B6
SSNA1	NSAIDs	Knowles DA et al., 2015 (13/025874)	SMC	Vitamin D
ZFAT	Opiates	Knowles DA et al., 2015 (13/025874)	LCL, PBMC	Aspirin, Loratadine
ZMAT2	Decongestant Medication	Knowles DA et al., 2015 (13/025874)	Mel	Triclosan
ATP5SL	Simvastatin	Mangravite LM et al., 2013 (23995691)	Mel, SMC	Retinoic Acid, Tunicamycin, Vitamin B6
ITFG2	Simvastatin	Mangravite LM et al., 2013 (23995691)	HUVEC	Mono-n-butyl Phthalate
C12orf45	Dexamethasone	Maranville JC et al., 2011 (21750684)	LCL, PBMC	Caffeine, Dexamethasone, Molybdenum, Vitamin B6, Zinc
CST7	Dexamethasone	Maranville JC et al., 2011 (21750684)	PBMC	Dexamethasone
LSG1	Dexamethasone	Maranville JC et al., 2011 (21750684)	HUVEC, LCL, Mel, PBMC, SMC	Benzophenone-3, Caffeine, Copper, Dexamethasone, Loratadine, Molybdenum, Mono-n-butyl Phthalate, Panthothenic acid, Selenium, Triclosan, Vitamin D
MCFD2	Dexamethasone	Maranville JC et al., 2011 (21750684)	LCL, PBMC	Bisphenol A, Vitamin E
MFGE8	Dexamethasone	Maranville JC et al., 2011 (21750684)	HUVEC, LCL	Bisphenol A, Caffeine, Insulin, Molybdenum, Mono-n-butyl Phthalate, Perfluorooctanoic Acid, Selenium, Triclosan, Vitamin D
NQO1	Dexamethasone	Maranville JC et al., 2011 (21750684)	Mel	Dexamethasone, Vitamin D
PANK3	Dexamethasone	Maranville JC et al., 2011 (21750684)	LCL, Mel, PBMC	Cadmium, Caffeine, Tunicamycin, Vitamin E
PDGFRL	Dexamethasone	Maranville JC et al., 2011 (21750684)	HUVEC, SMC	Dexamethasone, Vitamin D
SGK1	Dexamethasone	Maranville JC et al., 2011 (21750684)	HUVEC	Caffeine
TNIP1	Dexamethasone	Maranville JC et al., 2011 (21750684)	Mel, SMC	Aldosterone, Insulin, Loratadine, Molybdenum
ACAT1	Dexamethasone	Maranville JC et al., 2013 (24055111)	HUVEC, Mel	Acetaminophen, Insulin, Loratadine, Selenium, Tunicamycin
ATF7IP	Dexamethasone	Maranville JC et al., 2013 (24055111)	HUVEC, LCL, Mel, PBMC, SMC	Acetaminophen, Caffeine, Mono-n-butyl Phthalate, Perfluorooctanoic Acid, Retinoic Acid, Selenium, Vitamin D, Zinc
FAM117A	Dexamethasone	Maranville JC et al., 2013 (24055111)	LCL	Caffeine
GORAB	Dexamethasone	Maranville JC et al., 2013 (24055111)	LCL, Mel, PBMC, SMC	Aspirin, Benzophenone-3, Cadmium, Caffeine, Insulin, Mono-n-butyl Phthalate, Retinoic Acid, Triclosan, Zinc
MGAT1	Dexamethasone	Maranville JC et al., 2013 (24055111)	LCL	Cadmium, Caffeine, Loratadine
MTA1	Dexamethasone	Maranville JC et al., 2013 (24055111)	LCL, PBMC, SMC	Acetylcholine, Acrylamide, Benzophenone-3, Caffeine, Cetirizine, Copper, Perfluorooctanoic Acid, Vitamin B3
PLEKHG3	Dexamethasone	Maranville JC et al., 2013 (24055111)	Mel	Acetaminophen, Triclosan
RAB23	Dexamethasone	Maranville JC et al., 2013 (24055111)	HUVEC	Selenium
RCAN3	Dexamethasone	Maranville JC et al., 2013 (24055111)	Mel, PBMC	Acetaminophen, Cadmium, Insulin
SULT1C4	Dexamethasone	Maranville JC et al., 2013 (24055111)	HUVEC	Aspirin, Benzophenone-3, Mono-n-butyl Phthalate, Triclosan
UGGT2	Dexamethasone	Maranville JC et al., 2013 (24055111)	HUVEC, Mel, SMC	Aspirin, BHA, Dexamethasone, Insulin, Retinoic Acid, Triclosan, Vitamin D

Table S14: **EDGE index values for each cell type/treatment subgroup.** EDGE index and confidence intervals are calculated as described in Section 10.4. Also shown for each subgroup is the number of significant ASE and cASE SNPs.

Cell Type	Treatment	EDGE index	Lower 95% CI	Upper 95% CI	ASE (10% FDR)	cASE (24% FDR)
HUVEC	Dexamethasone	1.215	1.240	1.192	103	7
HUVEC	Caffeine	1.247	1.274	1.223	79	6
HUVEC	Molybdenum	1.209	1.236	1.184	84	3
HUVEC	Selenium	1.239	1.266	1.215	102	7
HUVEC	Vitamin D	1.289	1.318	1.261	92	4
HUVEC	Acrylamide	0.874	0.878	0.871	210	0
HUVEC	BP-3	0.964	0.972	0.955	136	0
HUVEC	Triclosan	1.020	1.033	1.008	137	3
HUVEC	Insulin	0.982	0.991	0.974	103	3
HUVEC	BHA	0.953	0.962	0.944	134	1
HUVEC	Aspirin	0.956	0.965	0.948	137	2
HUVEC	Phthalate	1.028	1.041	1.016	118	4
HUVEC	Vitamin A	1.232	1.260	1.206	83	7
LCL	Dexamethasone	1.274	1.303	1.248	89	2
LCL	Caffeine	1.528	1.588	1.475	65	9
LCL	Copper	1.371	1.413	1.333	86	5
LCL	Selenium	1.509	1.571	1.455	69	9
LCL	Acrylamide	0.898	0.903	0.894	129	0
LCL	BPA	0.925	0.932	0.918	100	0
LCL	Cadmium	1.019	1.032	1.006	75	4
LCL	PFOA	0.991	1.001	0.981	82	1
LCL	Aspirin	0.942	0.950	0.934	92	0
LCL	Loratadine	0.939	0.945	0.932	121	0
LCL	Cetirizine	1.021	1.035	1.008	76	2
LCL	Phthalate	1.053	1.067	1.040	86	1
LCL	Vitamin A	1.242	1.267	1.220	89	3
Mel	Dexamethasone	1.071	1.093	1.051	90	1
Mel	Caffeine	1.093	1.119	1.070	71	0
Mel	Selenium	1.105	1.134	1.079	65	2
Mel	Tunicamycin	1.208	1.245	1.175	74	4
Mel	Vitamin D	1.095	1.118	1.073	94	2
Mel	Triclosan	1.053	1.066	1.041	113	0
Mel	Insulin	1.100	1.115	1.086	124	0
Mel	BHA	1.046	1.059	1.035	122	3
Mel	Ibuprofen	1.072	1.085	1.059	129	2
Mel	Acetaminophen	1.055	1.068	1.043	116	5
Mel	Aspirin	1.010	1.021	1.000	127	3
Mel	Loratadine	1.059	1.073	1.047	113	3
Mel	Phthalate	1.071	1.085	1.058	114	4
Mel	Vitamin A	1.094	1.119	1.071	85	0
Mel	Vitamin E	1.106	1.131	1.083	86	1
PBMC	Dexamethasone	1.136	1.155	1.119	126	1
PBMC	Caffeine	1.189	1.211	1.168	108	3
PBMC	Nicotine	1.139	1.159	1.121	82	2
PBMC	Copper	1.066	1.080	1.054	103	2
PBMC	Iron (C1)	1.146	1.167	1.127	82	0
PBMC	Molybdenum	1.076	1.091	1.061	97	2
PBMC	Zinc	1.079	1.094	1.066	109	1

Continued on next page...

Table S14 – continued from previous page						
**Cell Type**	**Treatment**	**EDGE index**	**Lower 95% CI**	**Upper 95% CI**	**ASE SNPs**	**cASE SNPs**
PBMC	Vitamin D	1.389	1.447	1.338	41	3
PBMC	Cadmium	1.144	1.162	1.126	119	2
PBMC	Vitamin H	1.033	1.045	1.022	109	2
PBMC	Acetylcholine	1.178	1.201	1.157	92	8
PBMC	Vitamin B3	1.051	1.065	1.037	102	0
PBMC	BHA	1.121	1.143	1.101	82	3
PBMC	Acetaminophen	1.198	1.222	1.176	96	3
PBMC	Aspirin	1.145	1.166	1.126	98	3
PBMC	Phthalate	1.198	1.222	1.175	92	4
PBMC	Vitamin B5	1.064	1.078	1.051	102	3
PBMC	Vitamin B6	1.060	1.073	1.047	105	2
PBMC	Vitamin A	1.080	1.096	1.066	105	2
PBMC	Vitamin E	1.026	1.039	1.014	104	0
SMC	Dexamethasone	1.060	1.072	1.048	127	3
SMC	Caffeine	1.082	1.095	1.069	103	3
SMC	Molybdenum	1.028	1.039	1.018	147	1
SMC	Selenium	1.072	1.085	1.061	117	2
SMC	Vitamin D	1.053	1.065	1.042	101	2
SMC	Acrylamide	0.979	0.988	0.971	129	0
SMC	BP-3	1.008	1.018	1.000	120	0
SMC	BPA	1.011	1.021	1.002	122	1
SMC	PFOA	1.007	1.017	0.997	106	1
SMC	Triclosan	0.997	1.006	0.989	132	1
SMC	Insulin	0.994	1.003	0.985	129	0
SMC	Acetaminophen	0.984	0.993	0.975	134	0
SMC	Aspirin	1.024	1.034	1.014	121	0
SMC	Vitamin B5	1.104	1.120	1.089	93	4
SMC	Vitamin B6	1.078	1.091	1.065	100	0
SMC	Vitamin A	1.052	1.063	1.041	116	0
SMC	Aldosterone	1.049	1.061	1.039	119	1

Table S15: **SNPs displaying induced ASE.** Each iASE SNP is listed with the inducing treatment and matched control, cell type, and gene. Also given is the adjusted read coverages (see Section 10.5) for the reference and alternate allele in both treatment and control conditions.

rsID	Treatment ID	Control ID	Cell Type	Gene	Adjusted Treatment Coverage (Reference)	Adjusted Control Coverage (Reference)	Adjusted Treatment Coverage (Alternate)	Adjusted Control Coverage (Alternate)
rs5390	T13C1	CO1	LCL	*GIPR*	0.416	0.010	0.055	0.010
rs77388906	T19C1	CO1	LCL	*C1D*	3.034	0.010	0.727	0.010
rs28485150	T6C1	CO2	LCL	*LRRFIP1*	0.415	0.010	0.026	0.010
rs932501	T19C1	CO1	LCL	*MACROD2*	0.597	0.056	0.140	0.056
rs17238053	T13C1	CO1	LCL	*SEMA5A*	0.353	0.071	0.010	0.010
rs1061837	T6C1	CO2	LCL	*CCDC69*	0.609	0.010	1.817	0.010
rs200499	T19C1	CO1	LCL	*HIST1H4J*	0.010	0.010	0.569	0.010
rs61741379	T42C1	CO1	SMC	*PLEKHG5*	0.228	0.041	0.054	0.012
rs650616	T26C1	CO1	SMC	*PINK1-AS*	0.263	0.044	0.051	0.010
rs2244262	T42C1	CO1	SMC	*RAB3B*	0.182	0.021	0.046	0.017
rs192449674	T42C1	CO1	SMC	*NBPF15*	0.323	0.058	0.083	0.012
rs2834218	T30C1	CO2	SMC	*TMEM50B*	0.351	0.010	0.045	0.010
rs226202	T26C1	CO1	SMC	*ATG10*	0.063	0.024	0.376	0.047
rs8191993	T42C1	CO1	SMC	*hsa-mir-490*	0.186	0.033	0.041	0.012
rs3735943	T42C1	CO1	SMC	*TRPA1*	0.021	0.010	0.149	0.025
rs6866	T26C1	CO1	SMC	*SNHG7*	0.321	0.066	0.073	0.010
rs12857	T13C1	CO1	Mel	*DYNLL1*	0.454	0.010	2.945	0.010
rs12615308	T13C1	CO1	Mel	*UBE2F*	1.081	0.010	0.219	0.010
rs3172404	T21C1	CO3	Mel	*CLDN1*	0.041	0.010	0.529	0.010
rs1051122	T41C1	CO2	Mel	*CRYZ*	2.477	0.010	0.974	0.010
rs1883	T30C1	CO2	Mel	*EIF5*	0.742	0.010	0.232	0.010
rs2110964	T47C1	CO2	Mel	*PRKD3*	0.910	0.010	0.186	0.010
rs57381261	T43C1	CO2	Mel	*AC007246.3*	0.266	0.053	0.035	0.010
rs2305222	T33C1	CO1	Mel	*ANKRD28*	0.590	0.010	0.042	0.010
rs200813578	T44C1	CO2	Mel	*EGR1*	0.548	0.010	0.100	0.010
rs7326277	T12C1	CO2	HUVEC	*FLT1*	0.944	0.010	0.098	0.010
rs7326277	T23C1	CO2	HUVEC	*FLT1*	1.055	0.010	0.306	0.010
rs155053	T23C1	CO2	HUVEC	*CAST*	0.366	0.010	0.037	0.010
rs2296198	T13C1	CO1	HUVEC	*RNF144B*	1.161	0.010	0.329	0.010
rs4619	T12C1	CO2	HUVEC	*IGFBP1*	0.024	0.010	0.312	0.010
rs12530729	T19C1	CO1	HUVEC	*ZNF117*	0.111	0.010	0.613	0.010
rs3807069	T19C1	CO1	HUVEC	*ZNF117*	0.102	0.010	0.669	0.010
rs73672486	T18C1	CO1	HUVEC	*SWI5*	1.060	0.010	0.239	0.010
rs12784633	T23C1	CO2	PBMC	*RP11-445P17.8*	0.303	0.021	0.010	0.021
rs11235851	T2C1	CO1	PBMC	*RAB6A*	0.630	0.010	0.079	0.010
rs11235851	T5C1	CO1	PBMC	*RAB6A*	0.840	0.010	0.156	0.010
rs11235851	T20C1	CO1	PBMC	*RAB6A*	0.454	0.010	0.023	0.010
rs11235851	T13C1	CO1	PBMC	*RAB6A*	0.565	0.010	0.040	0.010
rs217086	T13C1	CO1	PBMC	*CTSC*	0.783	0.010	2.357	0.010
rs2239008	T16C1	CO1	PBMC	*MMP1*	0.164	0.010	1.254	0.010
rs470215	T16C1	CO1	PBMC	*MMP1*	0.059	0.010	0.478	0.010
rs10488	T16C1	CO1	PBMC	*MMP1*	1.357	0.010	0.251	0.010
rs28675952	T12C1	CO2	PBMC	*RP11-324E6.6*	0.073	0.010	0.429	0.010
rs11160859	T4C1	CO1	PBMC	*IGHG2*	0.502	0.039	0.029	0.019
rs11160859	T14C1	CO1	PBMC	*IGHG2*	0.346	0.037	0.037	0.019
rs2591050	T14C1	CO1	PBMC	*LINC00926*	0.039	0.010	0.525	0.010
rs121565	T6C1	CO2	PBMC	*CCL22*	2.494	0.010	0.674	0.010
rs121565	T7C1	CO2	PBMC	*CCL22*	2.304	0.010	0.780	0.010
rs3027955	T2C1	CO1	PBMC	*SLC1A5*	1.016	0.010	0.088	0.010
rs3027955	T4C1	CO1	PBMC	*SLC1A5*	0.925	0.010	0.088	0.010
rs3027955	T13C1	CO1	PBMC	*SLC1A5*	0.371	0.010	0.024	0.010
rs1130094	T5C1	CO1	PBMC	*ADAM17*	1.186	0.010	0.312	0.010
rs7583955	T4C1	CO1	PBMC	*AC009950.2*	0.531	0.010	0.068	0.010
rs1106639	T16C1	CO1	PBMC	*D2HGDH*	0.338	0.010	1.263	0.010
rs1106639	T14C1	CO1	PBMC	*D2HGDH*	0.131	0.010	0.663	0.010
rs8139993	T15C1	CO1	PBMC	*DESI1*	0.119	0.010	0.698	0.010
rs111462360	T20C1	CO1	PBMC	*LINC00847*	0.419	0.051	0.051	0.010
rs17295741	T2C1	CO1	PBMC	*NCF1*	0.123	0.010	0.622	0.010
rs17295741	T13C1	CO1	PBMC	*NCF1*	0.040	0.010	0.476	0.010
rs10264853	T13C1	CO1	PBMC	*UPK3B*	0.066	0.010	0.554	0.010
rs202105684	T7C1	CO2	PBMC	*UPK3B*	0.315	0.010	0.053	0.010
ss1388100066	T40C1	CO2	HUVEC	*RP11-22B23.1*	0.066	0.010	0.376	0.080
rs2834218	T24C1	CO1	HUVEC	*TMEM50B*	0.313	0.021	0.053	0.010
rs7289170	T24C1	CO1	HUVEC	*CECR1*	0.015	0.010	0.155	0.030
rs7726384	T24C1	CO1	HUVEC	*SREK1IP1*	0.011	0.010	0.234	0.034
rs1047494	T24C1	CO1	HUVEC	*IQGAP2*	0.136	0.019	0.019	0.010
rs4656992	T18C1	CO1	SMC	*ADAMTS4*	0.076	0.014	0.241	0.036
rs76197396	T18C1	CO1	SMC	*HERC2P3*	0.403	0.064	0.045	0.011
rs3743104	T9C1	CO2	SMC	*GREM1*	0.475	0.010	4.186	0.010
rs2303262	T4C1	CO1	SMC	*MPHOSPH6*	0.052	0.030	0.322	0.037
rs2292843	T18C1	CO1	SMC	*PRDM8*	0.011	0.010	0.118	0.010
rs3932940	T18C1	CO1	SMC	*CCDC127*	0.011	0.010	0.104	0.010
rs1012899	T12C1	CO2	SMC	*LRRC16A*	0.427	0.010	0.119	0.010
rs2502598	T18C1	CO1	SMC	*SYNJ2*	0.216	0.028	0.017	0.010
rs1134170	T4C1	CO1	SMC	*COL5A1*	5.536	0.010	1.519	0.010

Table S16: **Summary of GWAS meta-analysis traits examined.** Shown for each trait is the trait abbreviation and the citation for the meta analysis study.

Abbreviation	Trait	Study
BMI	Body mass index	Speliotes, E.K., et al. (2010). Nat. Genet. 42, 937-948
CD	Chron disease	Jostins, L., et al. (2012). Nature 491, 119-124
FG	Fasting glucose levels	Manning, A.K., et al. (2012). Nat. Genet. 44, 659-669
FNBMD	Bone mineral density (femur)	Estrada, K., et al. (2012). Nat. Genet. 44, 491-501
HB	Hemoglobin levels	van der Harst, P., et al. (2012). Nature 492, 369-375
HDL	HDL cholesterol levels	Teslovich, T.M., et al. (2010). Nature 466, 707-713
Height	Height	Lango Allen, H., et al. (2010). Nature 467, 832-838
LDL	LDL cholesterol levels	Teslovich, T.M., et al. (2010). Nature 466, 707-713
LSBMD	Bone mineral density (lumbar spine)	Estrada, K., et al. (2012). Nat. Genet. 44, 491-501
MCH	Mean red blood cell hemoglobin	van der Harst, P., et al. (2012). Nature 492, 369-375
MCHC	Mean corpuscular hemoglobin concentration	van der Harst, P., et al. (2012). Nature 492, 369-375
MCV	Mean red blood cell volume	van der Harst, P., et al. (2012). Nature 492, 369-375
MPV	Mean platelet volume	Gieger, C., et al. (2011). Nature 480, 201-208
PCV	Packed red blood cell volume	van der Harst, P., et al. (2012). Nature 492, 369-375
PLT	Platelet counts	Gieger, C., et al. (2011). Nature 480, 201-208
RBC	Red blood cell count	van der Harst, P., et al. (2012). Nature 492, 369-375
TC	Total cholesterol levels	Teslovich, T.M., et al. (2010). Nature 466, 707-713
TG	Triglyceride levels	Teslovich, T.M., et al. (2010). Nature 466, 707-713

Table S17: **GEMMA per SNP heritability estimates relative to the genomic average.** Shown for each GWAS trait tested (see Table S16) are the estimates for cASE/iASE (SNPs in genic regions with cASE or iASE), ASE (SNPs in genic regions with ASE), No ASE (SNPs in genic regions without ASE) and Intergenic (SNPs farther than 100kb from any gene). PVE: proportion of variance in phenotypes explained.

GWAS Trait	Category	PVE	Std Err	σ^2	Std Err	Enrichment	Std Err
BMI	cASE/iASE	0.0003	0.0001	5.96E-09	2.17E-09	0.9697	0.3819
BMI	ASE	0.0026	0.0010	1.68E-08	6.11E-09	2.7306	0.9696
BMI	Intergenic	0.0021	0.0006	5.69E-09	1.50E-09	0.9260	0.2757
BMI	No ASE	0.0267	0.0036	5.82E-09	7.81E-10	0.9469	0.0407
CD	cASE/iASE	0.0005	0.0007	8.60E-09	1.43E-08	0.0957	0.1619
CD	ASE	0.0229	0.0032	1.45E-07	2.03E-08	1.6199	0.2546
CD	Intergenic	0.0004	0.0067	9.88E-10	1.81E-08	0.0110	0.2015
CD	No ASE	0.4402	0.0314	9.60E-08	6.84E-09	1.0687	0.0169
FG	cASE/iASE	0.0000	0.0002	5.83E-10	3.86E-09	0.0847	0.5609
FG	ASE	0.0002	0.0006	1.56E-09	4.08E-09	0.2267	0.5973
FG	Intergenic	-0.0014	0.0009	-3.78E-09	2.56E-09	-0.5488	0.3597
FG	No ASE	0.0365	0.0070	7.99E-09	1.52E-09	1.1616	0.0364
FNBMD	cASE/iASE	0.0013	0.0004	2.42E-08	6.73E-09	5.1713	2.6679
FNBMD	ASE	0.0028	0.0011	1.76E-08	6.97E-09	3.7533	2.3087
FNBMD	Intergenic	-0.0092	0.0015	-2.50E-08	4.02E-09	-5.3267	2.3578
FNBMD	No ASE	0.0294	0.0109	6.41E-09	2.37E-09	1.3671	0.1246
HB	cASE/iASE	0.0025	0.0003	4.86E-08	4.93E-09	6.7590	0.0150
HB	ASE	0.0001	0.0006	6.81E-10	3.83E-09	0.0946	0.5336
HB	Intergenic	-0.0053	0.0008	-1.43E-08	2.19E-09	-1.9883	0.3669
HB	No ASE	0.0398	0.0062	8.67E-09	1.34E-09	1.2058	0.0297
HDL	cASE/iASE	0.0013	0.0002	2.57E-08	4.24E-09	2.6991	0.5198
HDL	ASE	0.0166	0.0029	1.06E-07	1.81E-08	11.1082	1.8199
HDL	Intergenic	-0.0059	0.0006	-1.61E-08	1.59E-09	-1.6912	0.2039
HDL	No ASE	0.0371	0.0067	8.09E-09	1.46E-09	0.8505	0.0593
HEIGHT	cASE/iASE	0.0017	0.0001	3.18E-08	2.84E-09	1.3101	0.1387
HEIGHT	ASE	0.0109	0.0008	6.91E-08	4.91E-09	2.8493	0.2311
HEIGHT	Intergenic	-0.0080	0.0007	-2.17E-08	1.80E-09	-0.8942	0.0718
HEIGHT	No ASE	0.1205	0.0065	2.63E-08	1.41E-09	1.0854	0.0091
LDL	cASE/iASE	0.0021	0.0004	3.96E-08	6.75E-09	5.8858	2.0932
LDL	ASE	0.0044	0.0009	2.81E-08	5.89E-09	4.1724	1.6154
LDL	Intergenic	-0.0043	0.0008	-1.18E-08	2.24E-09	-1.7451	0.3600
LDL	No ASE	0.0327	0.0116	7.11E-09	2.53E-09	1.0563	0.0593
LSBMD	cASE/iASE	0.0030	0.0005	5.79E-08	1.03E-08	13.8333	6.5121
LSBMD	ASE	0.0029	0.0012	1.86E-08	7.65E-09	4.4363	2.8557
LSBMD	Intergenic	-0.0074	0.0016	-2.01E-08	4.39E-09	-4.7997	2.3134
LSBMD	No ASE	0.0231	0.0110	5.04E-09	2.40E-09	1.2022	0.1098
MCH	cASE/iASE	0.0091	0.0007	1.73E-07	1.34E-08	8.6428	0.3575
MCH	ASE	0.0007	0.0009	4.71E-09	5.99E-09	0.2355	0.3093
MCH	Intergenic	-0.0033	0.0013	-8.88E-09	3.47E-09	-0.4442	0.1560
MCH	No ASE	0.0968	0.0123	2.11E-08	2.68E-09	1.0552	0.0164
MCHC	cASE/iASE	0.0005	0.0002	9.88E-09	4.47E-09	-1.2357	0.6014
MCHC	ASE	-0.0003	0.0006	-1.91E-09	3.85E-09	0.2395	0.4845
MCHC	Intergenic	-0.0039	0.0008	-1.06E-08	2.17E-09	1.3290	0.3229
MCHC	No ASE	-0.0376	0.0047	-8.19E-09	1.03E-09	1.0251	0.0302
MCV	cASE/iASE	0.0063	0.0005	1.21E-07	9.74E-09	5.8436	0.3254
MCV	ASE	0.0019	0.0007	1.22E-08	4.50E-09	0.5892	0.2407
MCV	Intergenic	-0.0036	0.0011	-9.80E-09	2.88E-09	-0.4737	0.1246

Continued on next page...

Table S17 – continued from previous page							
**GWAS Trait**	**Category**	**PVE**	**Std Err**	σ^2	**Std Err**	**Enrichment**	**Std Err**
MCV	No ASE	0.1023	0.0102	2.23E-08	2.23E-09	1.0774	0.0138
MPV	cASE/iASE	0.0032	0.0005	6.19E-08	9.40E-09	2.9909	0.6316
MPV	ASE	0.0251	0.0039	1.60E-07	2.46E-08	7.7145	1.4648
MPV	Intergenic	-0.0114	0.0017	-3.09E-08	4.66E-09	-1.4916	0.2313
MPV	No ASE	0.0899	0.0153	1.96E-08	3.34E-09	0.9475	0.0524
PCV	cASE/iASE	0.0019	0.0003	3.60E-08	5.12E-09	6.1117	0.9515
PCV	ASE	-0.0015	0.0006	-9.42E-09	3.90E-09	-1.5979	0.7217
PCV	Intergenic	-0.0072	0.0009	-1.94E-08	2.39E-09	-3.2921	0.6572
PCV	No ASE	0.0372	0.0057	8.11E-09	1.23E-09	1.3761	0.0595
PLT	cASE/iASE	0.0036	0.0003	6.83E-08	6.25E-09	3.5299	0.3717
PLT	ASE	0.0119	0.0013	7.54E-08	8.28E-09	3.8947	0.4394
PLT	Intergenic	-0.0089	0.0007	-2.40E-08	1.99E-09	-1.2390	0.1022
PLT	No ASE	0.0934	0.0064	2.04E-08	1.39E-09	1.0520	0.0183
RBC	cASE/iASE	0.0015	0.0003	2.87E-08	5.11E-09	1.8908	0.3775
RBC	ASE	0.0004	0.0007	2.29E-09	4.56E-09	0.1509	0.3031
RBC	Intergenic	-0.0033	0.0010	-9.03E-09	2.78E-09	-0.5939	0.1727
RBC	No ASE	0.0800	0.0084	1.74E-08	1.84E-09	1.1472	0.0188
TC	cASE/iASE	0.0048	0.0005	9.12E-08	9.10E-09	9.6485	1.8248
TC	ASE	0.0043	0.0009	2.73E-08	5.69E-09	2.8829	0.8570
TC	Intergenic	-0.0063	0.0007	-1.69E-08	2.02E-09	-1.7925	0.2413
TC	No ASE	0.0460	0.0104	1.00E-08	2.27E-09	1.0614	0.0358
TG	cASE/iASE	0.0037	0.0005	7.06E-08	9.64E-09	7.9232	0.0172
TG	ASE	0.0152	0.0034	9.67E-08	2.18E-08	10.8507	2.8737
TG	Intergenic	-0.0062	0.0008	-1.68E-08	2.04E-09	-1.8806	0.3053
TG	No ASE	0.0333	0.0098	7.26E-09	2.13E-09	0.8149	0.0807

Table S18: **Overlap of GWAS-identified genes and genes containing cASE or iASE SNPs.** The table shows genes that have previously been associated with a phenotype through GWAS and contain evidence of cASE (79 genes with 87 SNPs) or iASE (28 genes with 35 SNPs). Also denoted are the treatment and cell type for which we identified cASE.

See attached file, Supplemental_Table_S18.xlsx, also available at `http://genome.grid.wayne.edu/gxebrowser/Tables/Supplemental_Table_S18.xlsx`

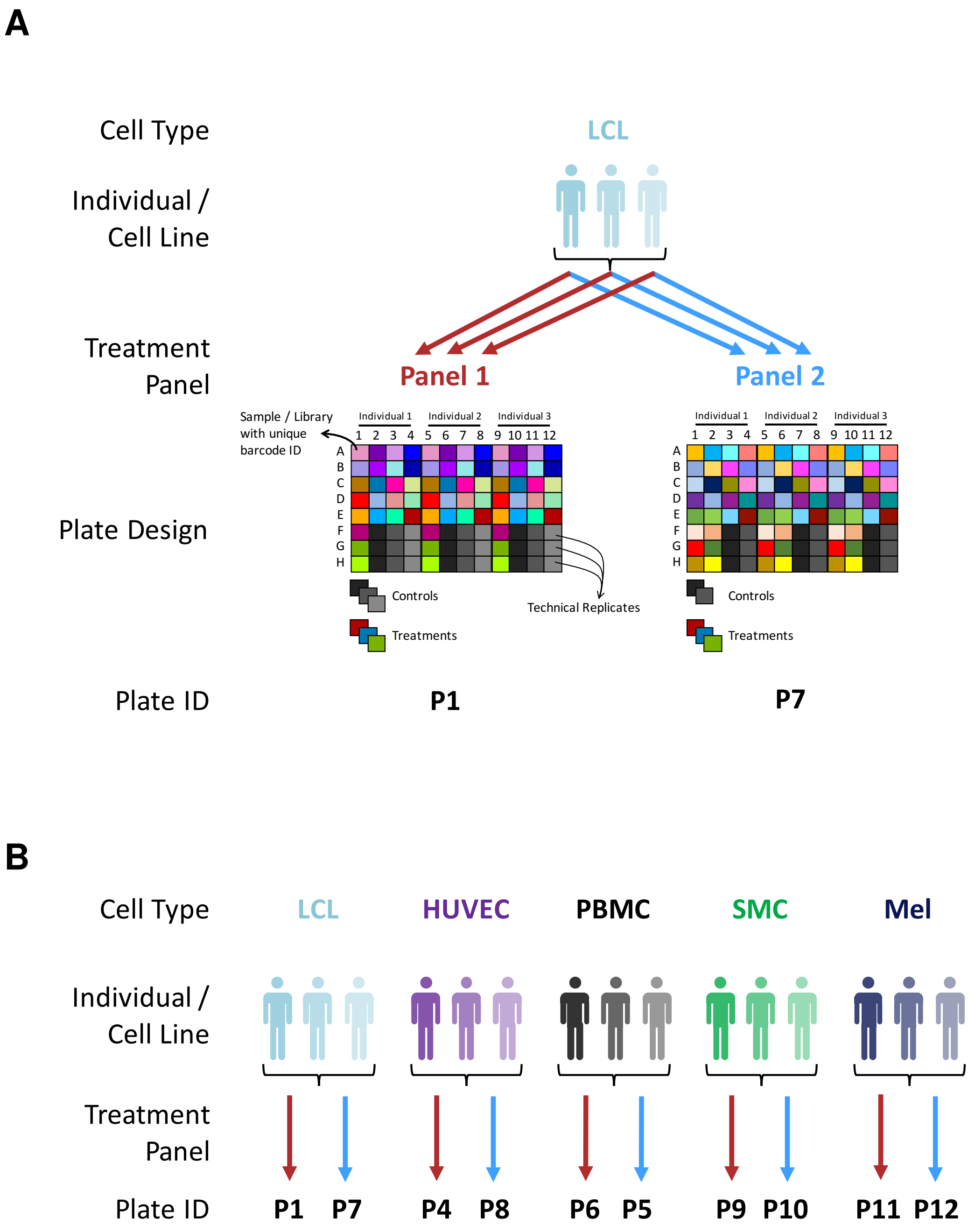

Figure S1: **Study design for one cell type (A) and all cell types (B).** (A) Each cell type is represented by three individuals, each treated with treatments belonging to two treatment panels. For each treatment panel, the three individuals were treated in parallel on the same plate to analyze 32 samples (corresponding to sequencing libraries with unique barcode IDs, and represented by the different colors on the plate design). The 32 conditions correspond to 23 treatments and 3 vehicle controls for panel one, and to 26 treatments and 2 vehicle controls for panel two. For each control sample (colored in shades of grey), three technical replicates were performed (i.e., triplicates of the same sample/library). (B) The study design described in (A) is repeated for each cell type in the study.

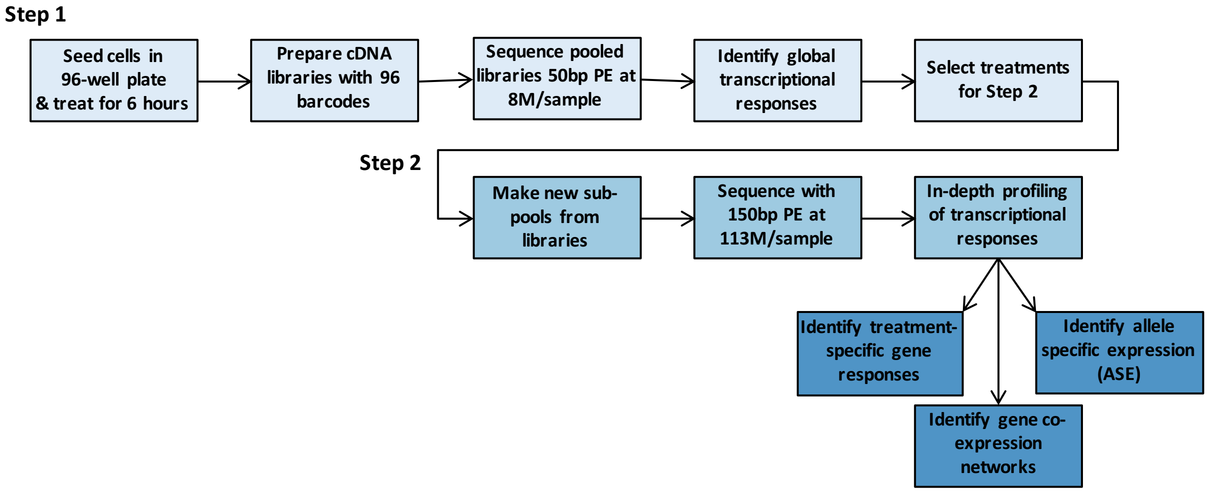

Figure S2: **Workflow of the two-step approach, modified from (*Moyerbrailean et al., 2015*).**

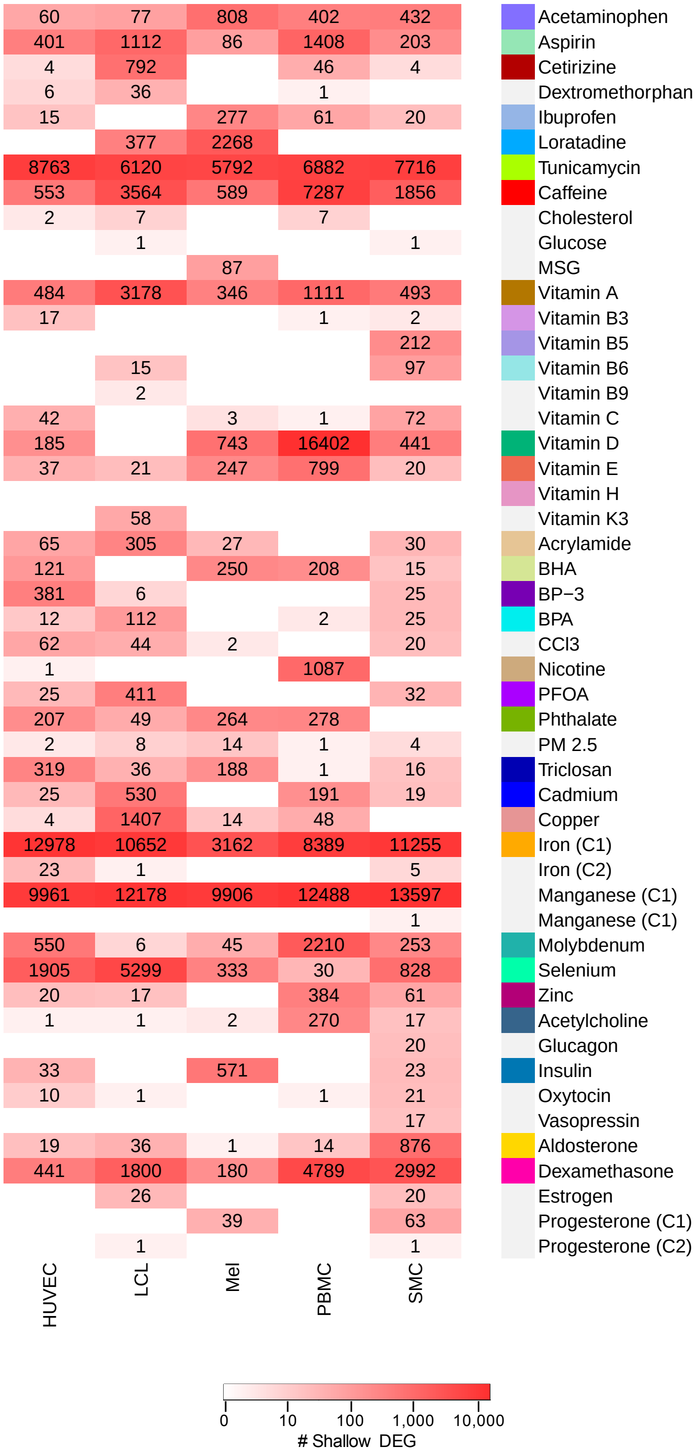

Treatment	HUVEC	LCL	Mel	PBMC	SMC
Acetaminophen	60	77	808	402	432
Aspirin	401	1112	86	1408	203
Cetirizine	4	792		46	4
Dextromethorphan	6	36		1	
Ibuprofen	15		277	61	20
Loratadine		377	2268		
Tunicamycin	8763	6120	5792	6882	7716
Caffeine	553	3564	589	7287	1856
Cholesterol	2	7		7	
Glucose		1			1
MSG			87		
Vitamin A	484	3178	346	1111	493
Vitamin B3	17			1	2
Vitamin B5					212
Vitamin B6		15			97
Vitamin B9		2			
Vitamin C	42		3	1	72
Vitamin D	185		743	16402	441
Vitamin E	37	21	247	799	20
Vitamin H					
Vitamin K3		58			
Acrylamide	65	305	27		30
BHA	121		250	208	15
BP−3	381	6			25
BPA	12	112		2	25
CCl3	62	44	2		20
Nicotine	1			1087	
PFOA	25	411			32
Phthalate	207	49	264	278	
PM 2.5	2	8	14	1	4
Triclosan	319	36	188	1	16
Cadmium	25	530		191	19
Copper	4	1407	14	48	
Iron (C1)	12978	10652	3162	8389	11255
Iron (C2)	23	1			5
Manganese (C1)	9961	12178	9906	12488	13597
Manganese (C1)					1
Molybdenum	550	6	45	2210	253
Selenium	1905	5299	333	30	828
Zinc	20	17		384	61
Acetylcholine	1	1	2	270	17
Glucagon					20
Insulin	33		571		23
Oxytocin	10	1		1	21
Vasopressin					17
Aldosterone	19	36	1	14	876
Dexamethasone	441	1800	180	4789	2992
Estrogen		26			20
Progesterone (C1)			39		63
Progesterone (C2)		1			1

Figure S3: **Heatmap of differential gene expression from Step 1.** Shown for each cell type (columns) and treatment (rows) combination are the number of differentially expressed genes (10% FDR and $|\log FC| > 0.25$). Number of differentially expressed genes is indicated by the number in the cell as well as the shade of red. Colors next to treatment names represent treatments chosen for deep sequencing. Grey indicates treatments that were not deep sequenced.

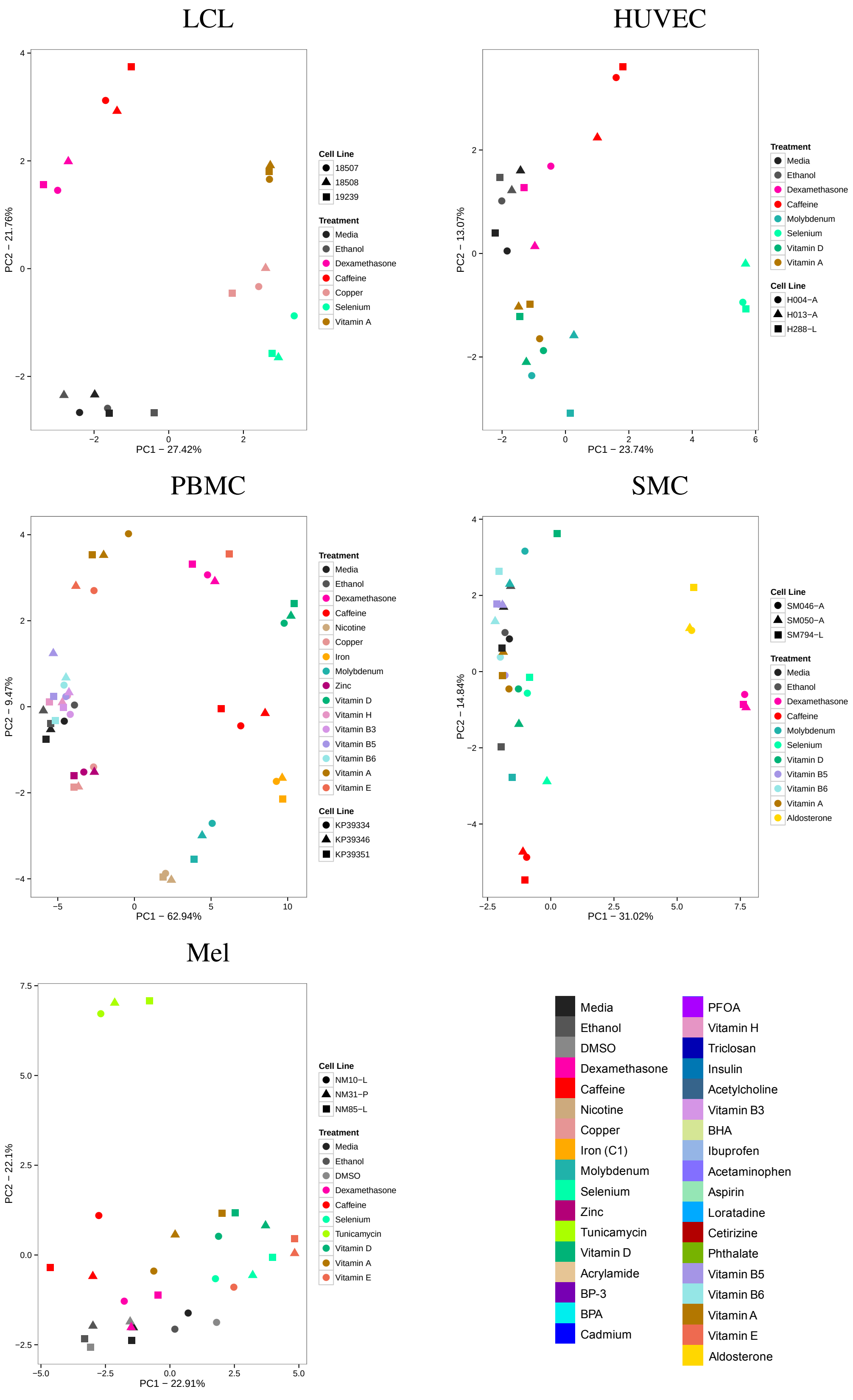

Figure S4: **Principal component analysis of gene expression levels across all deep sequenced samples, for Panel 1 treatments.** Gene expression levels (FPKMs) were obtained for each sample (individual and treatment combination) as a vector indexed by gene. Those vectors were correlated and PCA was performed on the resulting correlation matrix.

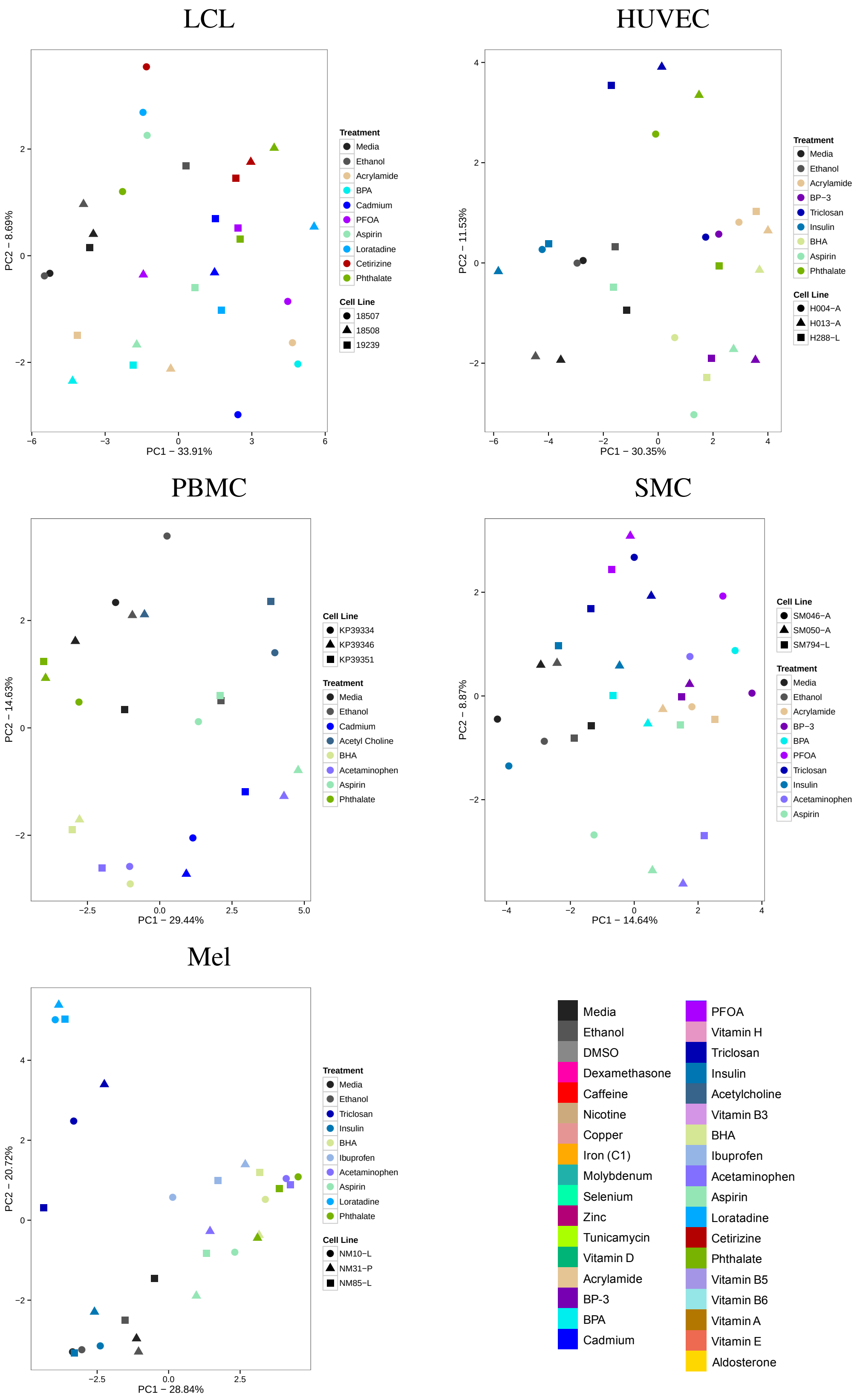

Figure S5: **Principal component analysis of gene expression levels across all deep sequenced samples, for Panel 2 treatments.** Gene expression levels (FPKMs) were obtained for each sample (individual and treatment combination) as a vector indexed by gene. Those vectors were correlated and PCA was performed on the resulting correlation matrix.

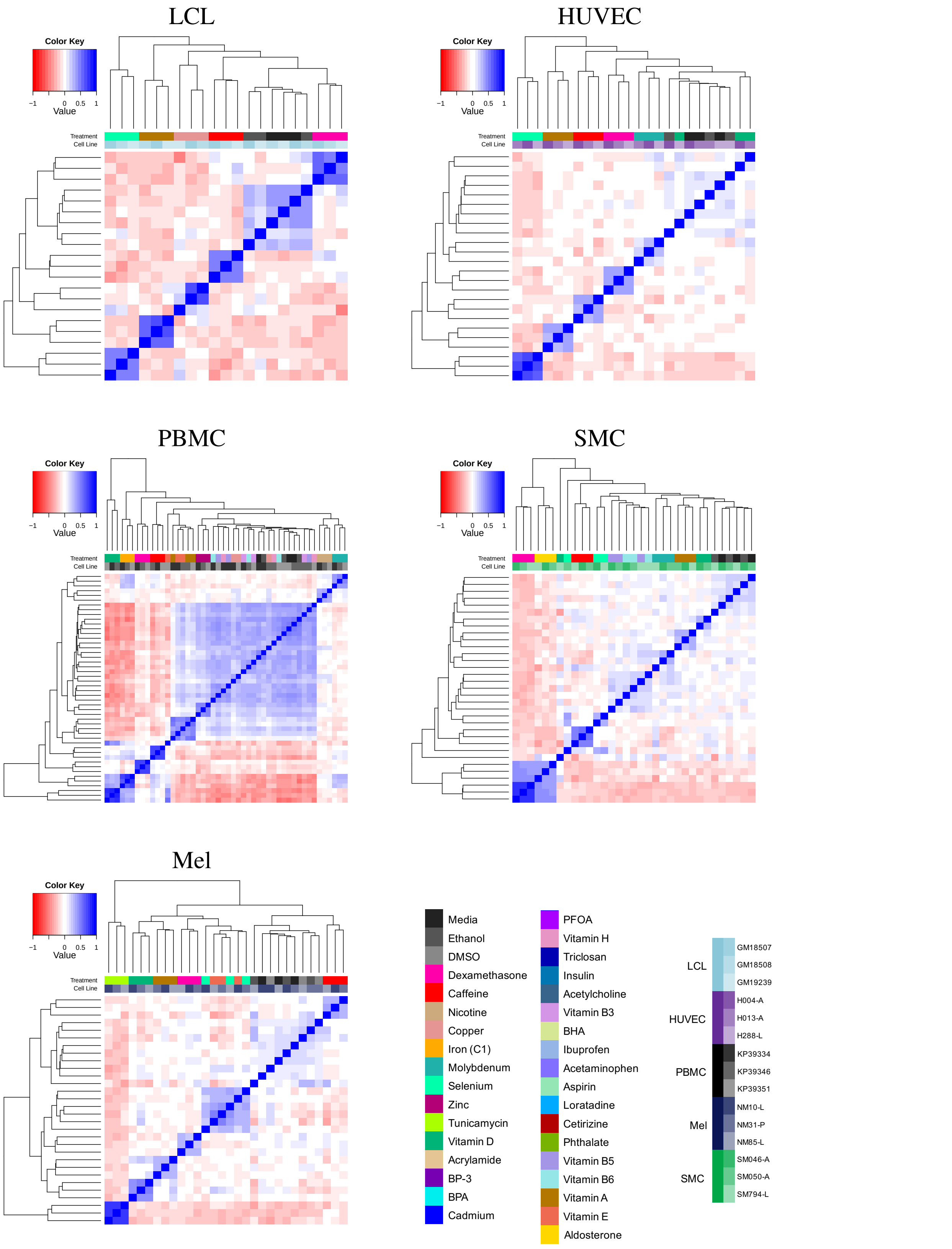

Figure S6: **Heatmap and hierarchical clustering of gene expression levels across all deep sequenced samples, for Panel 1 treatments.** Gene expression levels (FPKMs) were obtained for each sample (individual and treatment combination) as a vector indexed by gene. Those vectors were clustered using hierarchical clustering and the dendrogram is displayed at the top of a heatmap visualizing the Pearson correlation between each sample. The sample identity is detailed by a two-way coloring indexing the individual and treatment (see legend).

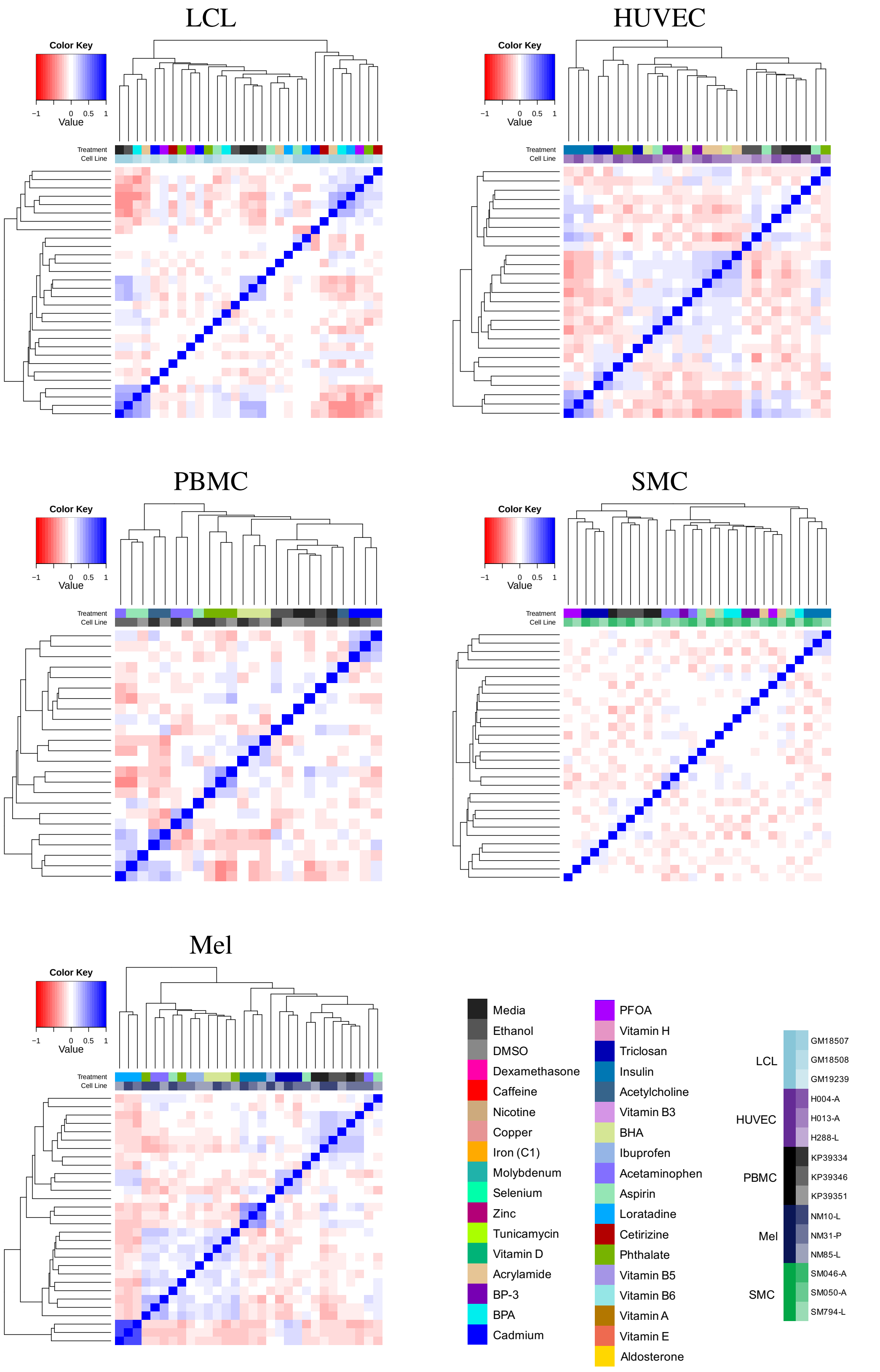

Figure S7: **Heatmap and hierarchical clustering of gene expression levels across all deep sequenced samples, for Panel 2 treatments.** Gene expression levels (FPKMs) were obtained for each sample (individual and treatment combination) as a vector indexed by gene. Those vectors were clustered using hierarchical clustering and the dendrogram is displayed at the top of a heatmap visualizing the Pearson correlation between each sample. The sample identity is detailed by a two-way coloring indexing the individual and treatment (see legend).

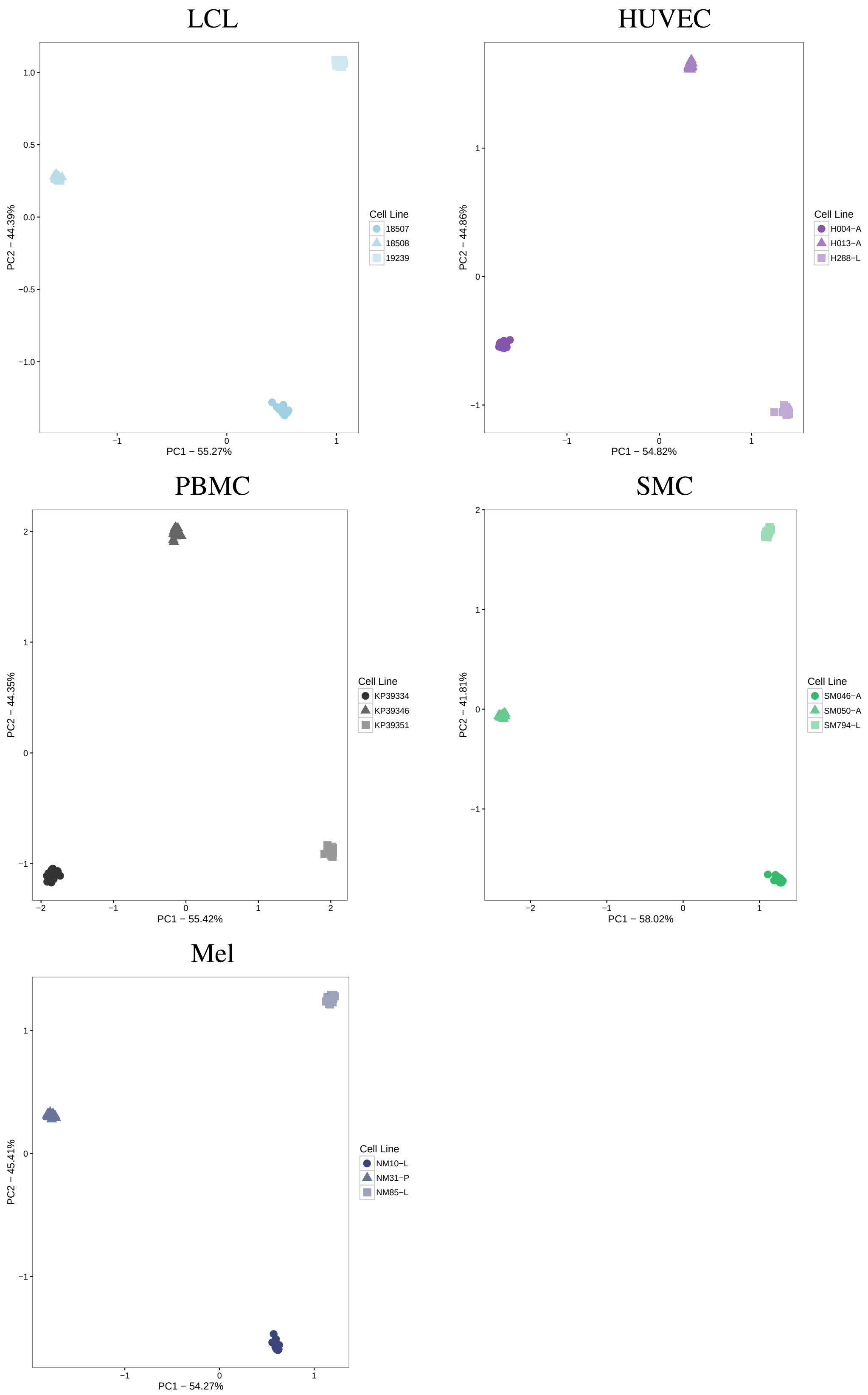

Figure S8: **Principal component analysis of the genotype data**. For each cell type, the allele ratio ($\hat{\rho}$) was correlated using all samples for each of the three individuals. PCA was performed on the correlation matrix, showing three distinct clusters representing each of the three individuals, confirming that there is no cross-individual contamination.

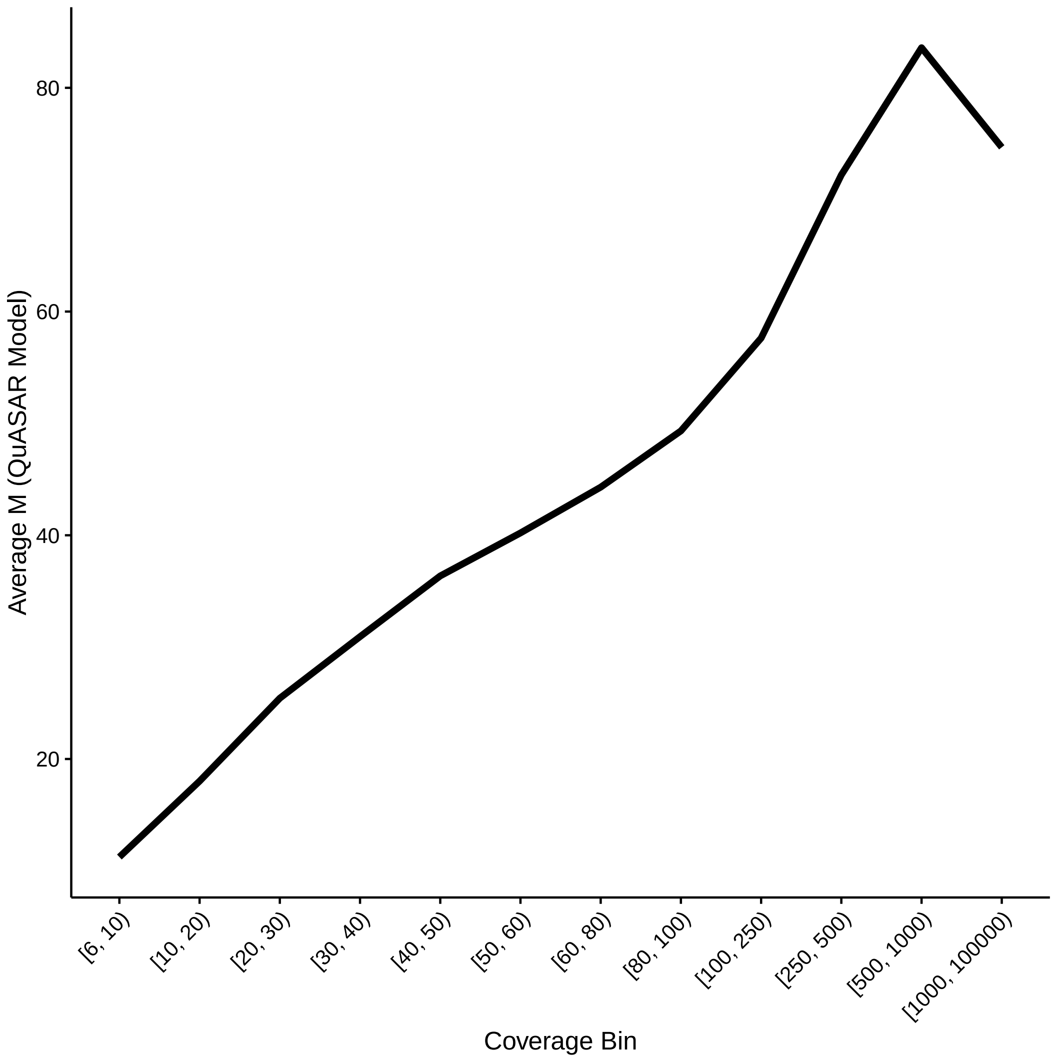

Figure S9: **Average M parameter estimates derived from the QuASAR model.** For each bin of coverage (total number of reads covering a SNP), plotted is the average estimated we obtained for the M parameter in beta-binomial. Lower M indicates higher over-dispersion of the read proportions in the beta-binomial model.

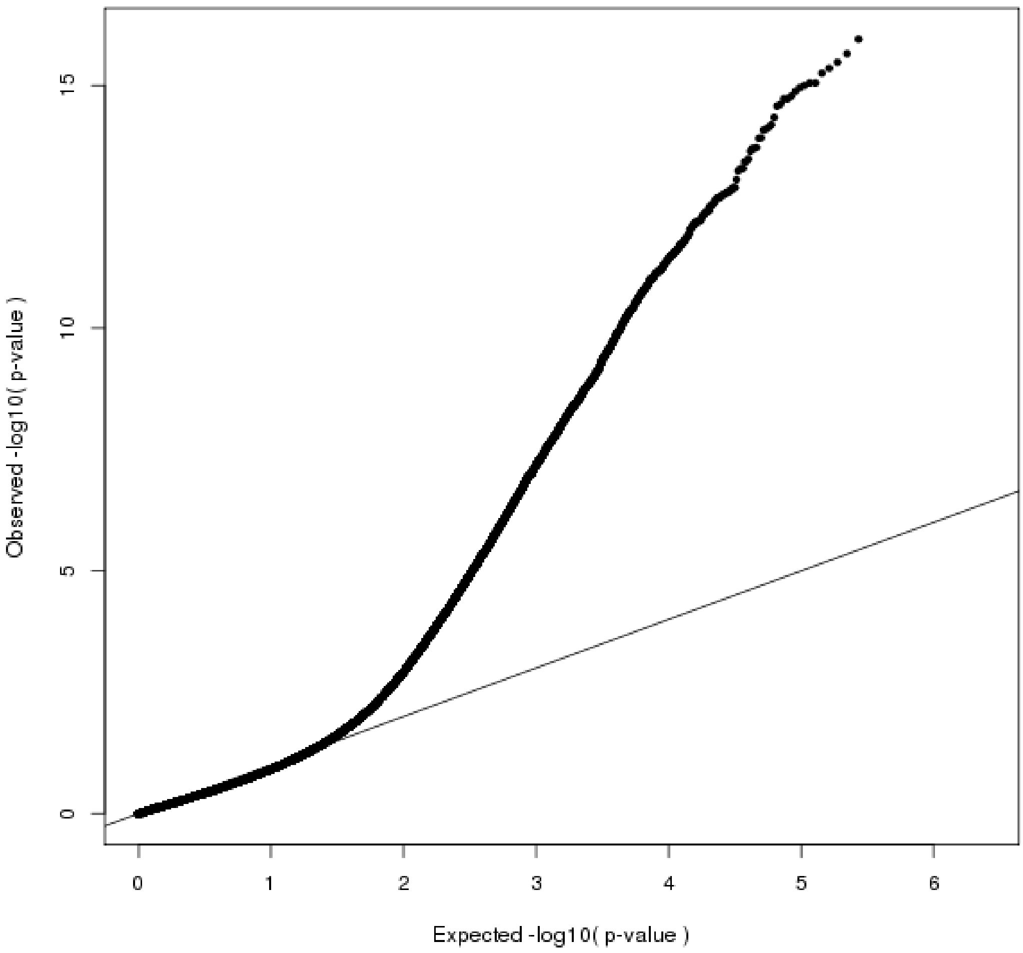

Figure S10: **QQ-plot of p-values from the QuASAR test for ASE.**

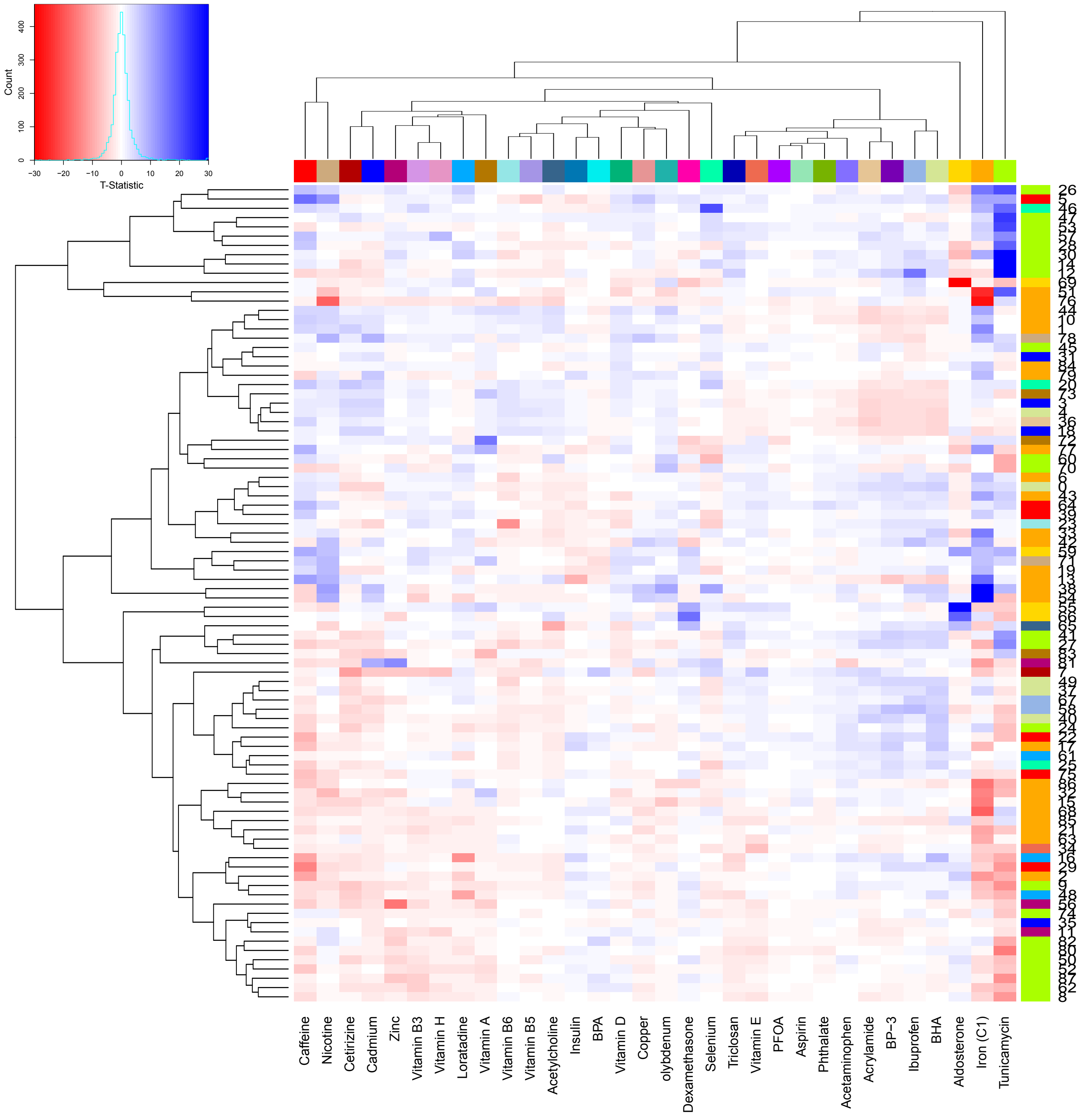

Figure S11: **Heatmap of the t-values calculated for each network module and treatment.**

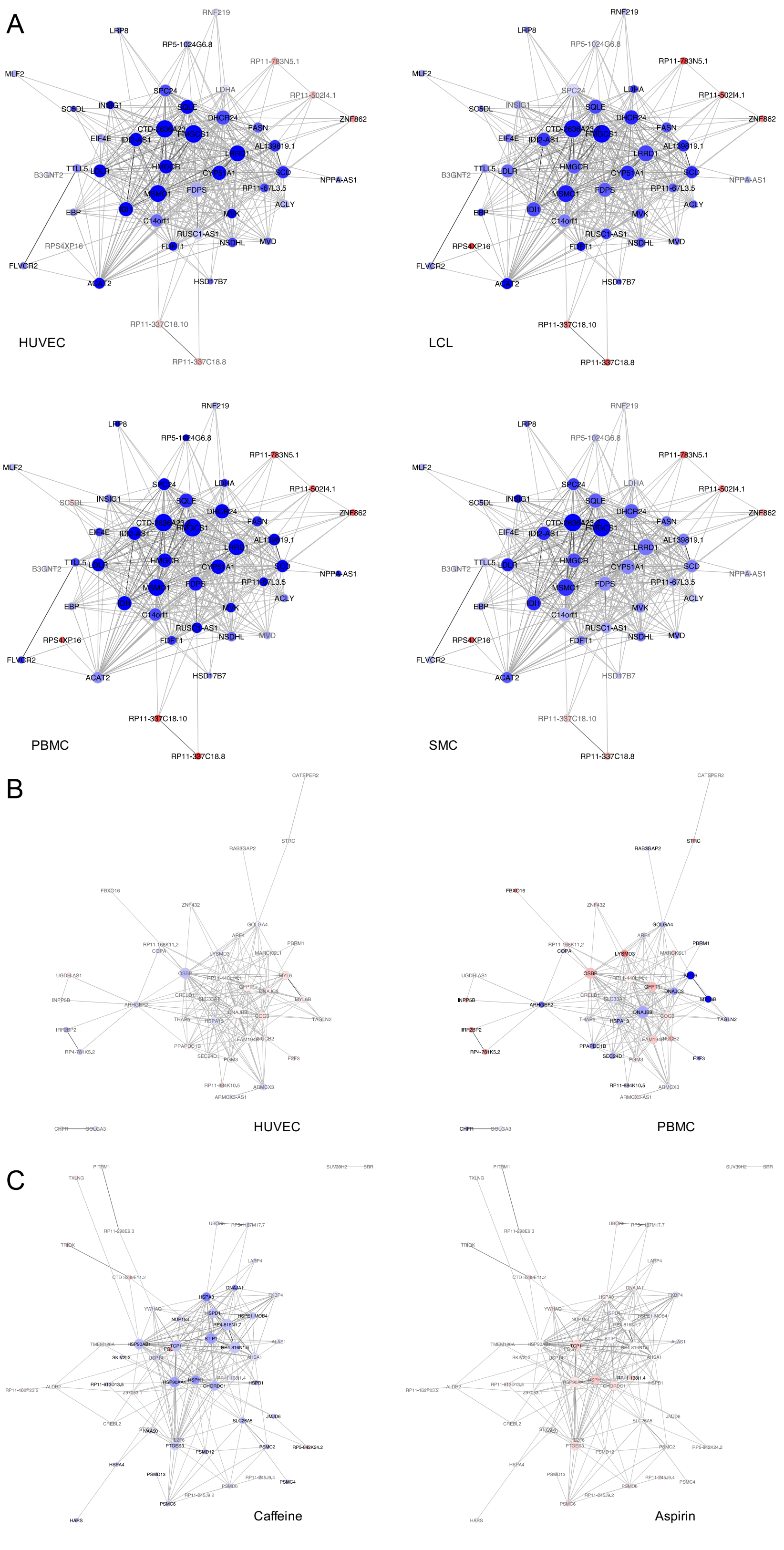

Figure S12: **Modules significantly associated with given cell type or treatments.** Nodes are sized proportional to their connectivity (normalized within a module) and colored based on the sign and magnitude of the gene expression log-fold change (red denotes an increase in expression following treatment). Gene names in black are significantly differentially expressed (1% FDR) following treatment. Edges are shaded based on the weight of the correlation between the two genes (black indicates stronger correlation across samples). (A) Module 29, associated with caffeine across various cell types. (B) Module 30, associated with vitamin D in HUVECs and PBMCs. (C) Module 22, associated with caffeine and aspirin in SMCs.

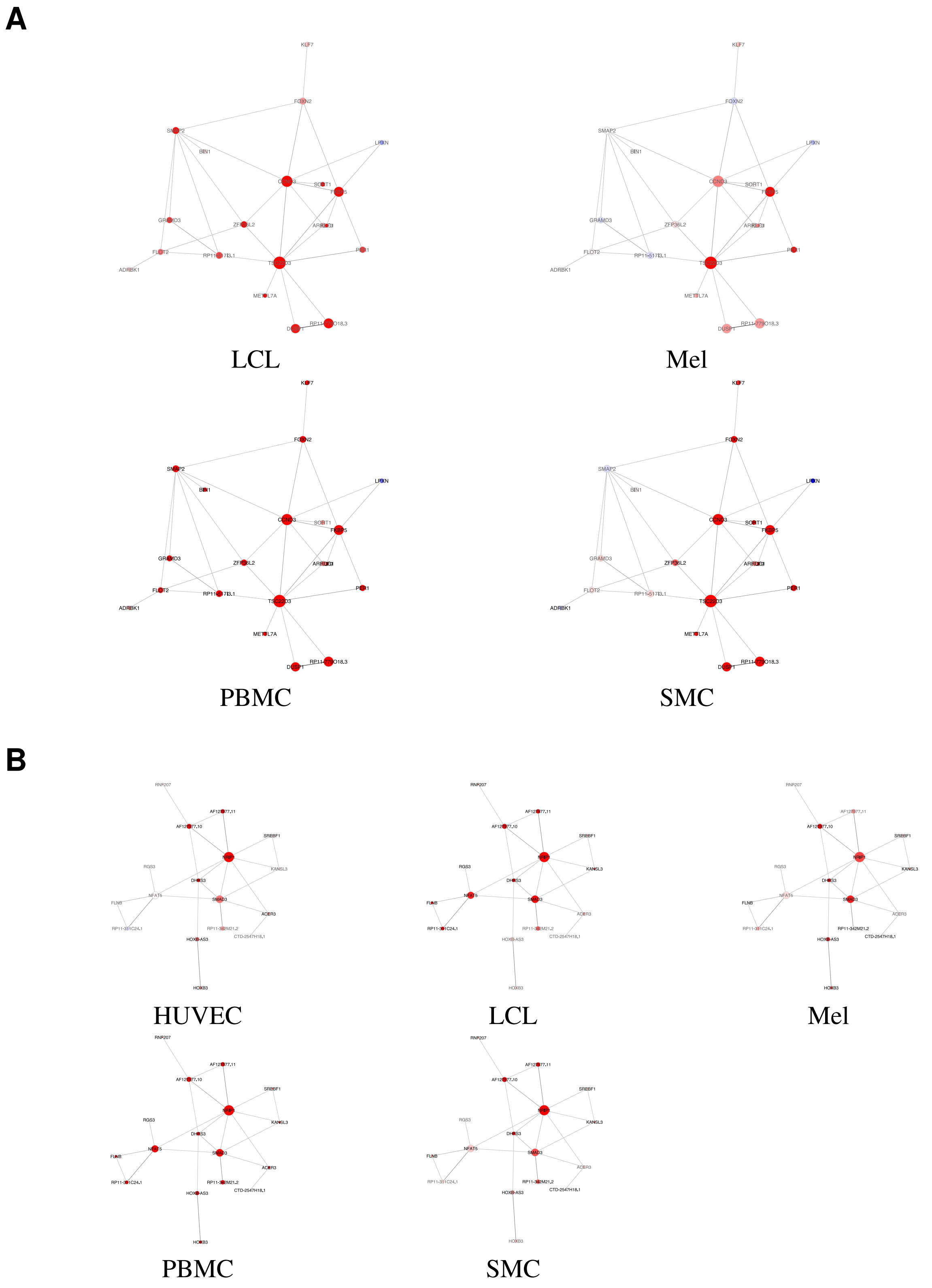

Figure S13: **Modules significantly associated with given cell type or treatments.** Nodes are sized proportional to their connectivity (normalized within a module) and colored based on the sign and magnitude of the gene expression log-fold change (red denotes an increase in expression following treatment). Gene names in black are significantly differentially expressed (1% FDR) following treatment. Edges are shaded based on the weight of the correlation between the two genes (black indicates stronger correlation across samples). (A) Dexamethasone (module 66). (B) Vitamin A (module 72). The product of module 72 hub gene *DHRS3* is known to attenuate vitamin A synthesis to maintain the correct balance of intracellular retinoic acid levels during body axis-formation (*Kam et al., 2013*).

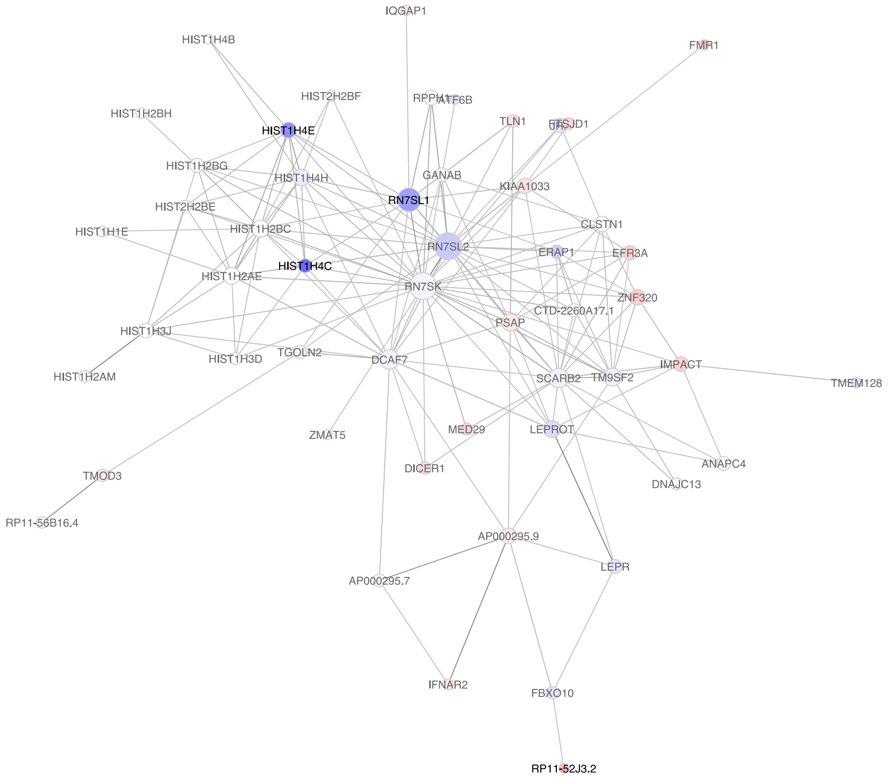

Figure S14: **Module 23 containing *ERAP1* in Melanocytes following Selenium.** Nodes are sized proportional to their connectivity (normalized within each module) and colored based on the sign and magnitude of the gene expression log-fold change (red denotes an increase in expression following treatment). Edges are shaded based on the weight of the correlation between the two genes (darker indicates stronger correlation between samples). Gene names in black are significantly differentially expressed (1% FDR) following treatment.

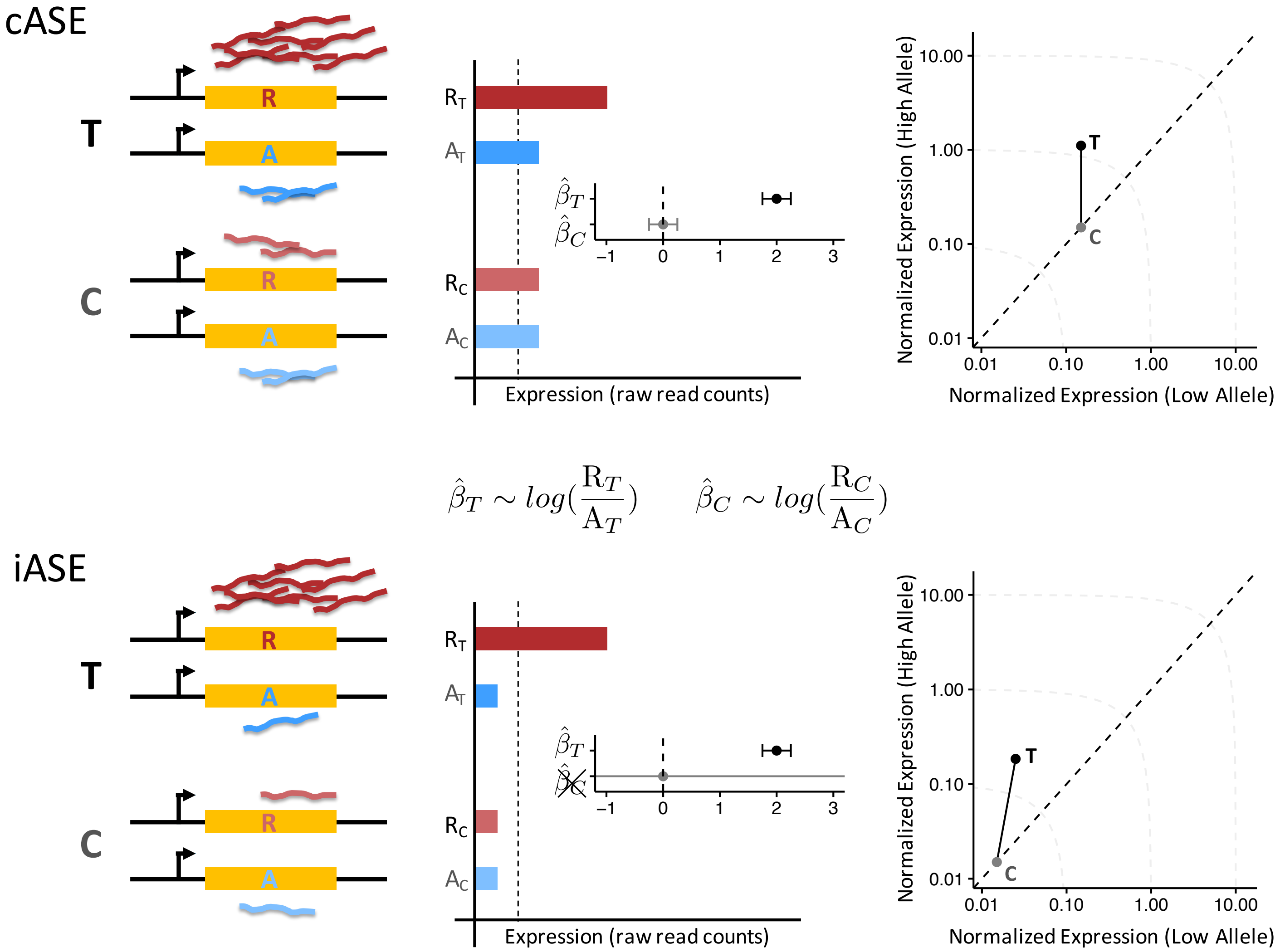

Figure S15: **Example diagrams and plots describing cASE and iASE SNPs.** Left, gene expression in treatment (T) and control (C) samples for both reference (R) and alternate (A) alleles. Middle, barplot of example expression levels. The dotted line represents a threshold for the detection of transcript expression. Inset is a forest plot of $\hat{\beta}$ values calculated from the reads covering each alleles, using the provided formulas. Note that in the control sample, the $\hat{\beta}$ is undefined for iASE. Right, scatter plot contrasting expression levels (normalized to library coverage) of the two alleles for both treatment (black point) and control (grey point). The line represents the magnitude of the fold-change between treatment and control. The dotted line represents equal expression of the two alleles (i.e., no ASE).

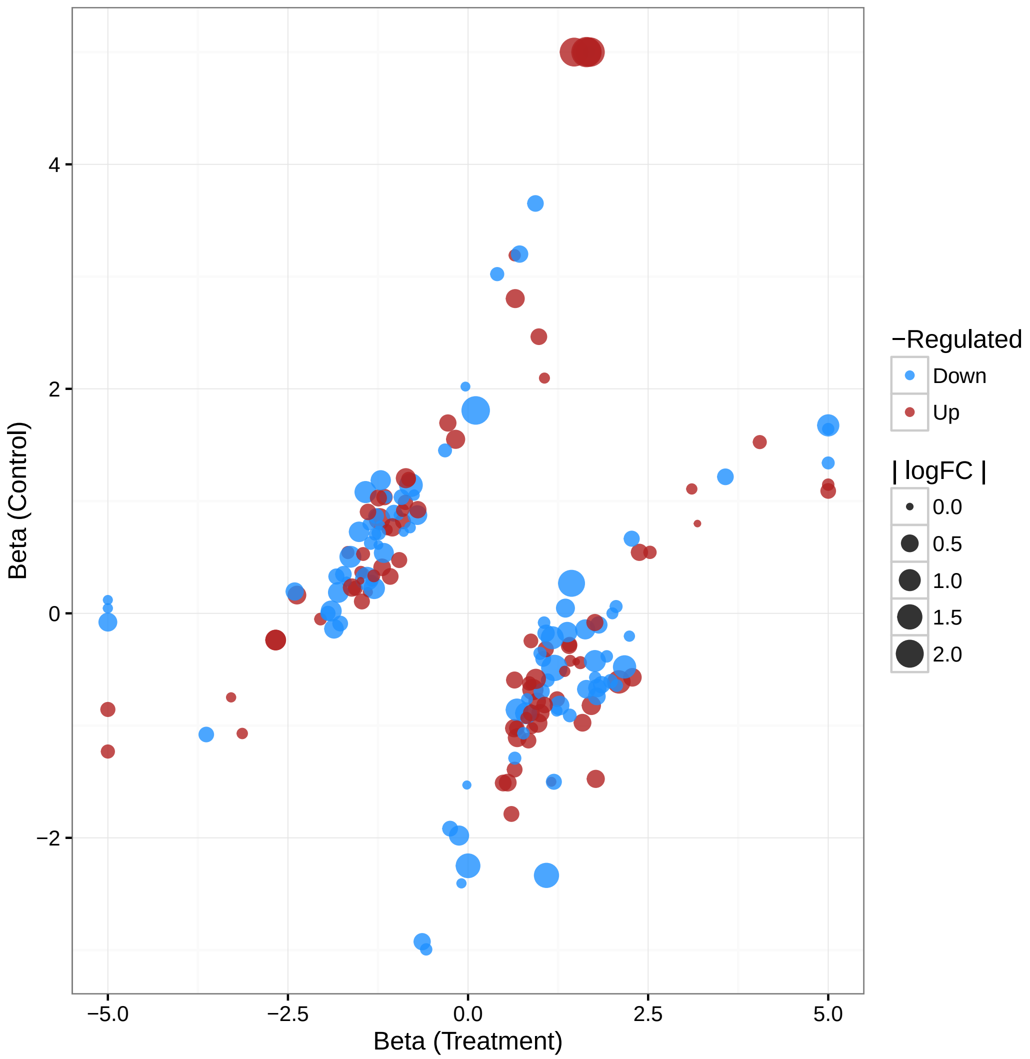

Figure S16: **Scatterplot of $\hat{\beta}$s for SNPs with cASE.** For each cASE SNP, plotted is the $\hat{\beta}$ from QuASAR for the control (y-axis) and treatment (x-axis) conditions. The size of the point indicates the degree of differential expression for the gene containing the SNP, while the color indicates whether the treatment causes the gene to be up- or downregulated.

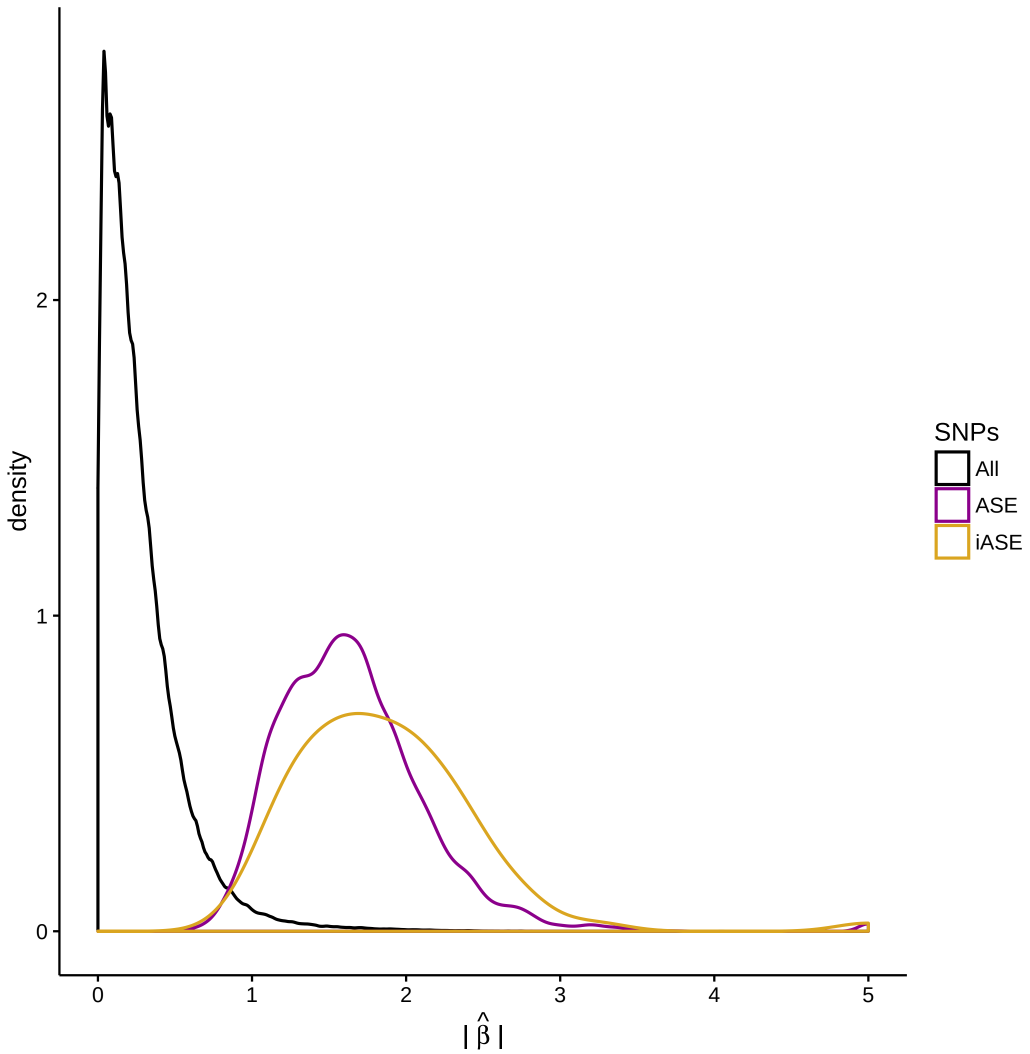

Figure S17: **Density of ASE $\hat{\beta}$ values across SNPs.** All, all SNPs tested for ASE; ASE, SNPs with ASE (10% FDR); iASE, SNPs with induced ASE (10% FDR).

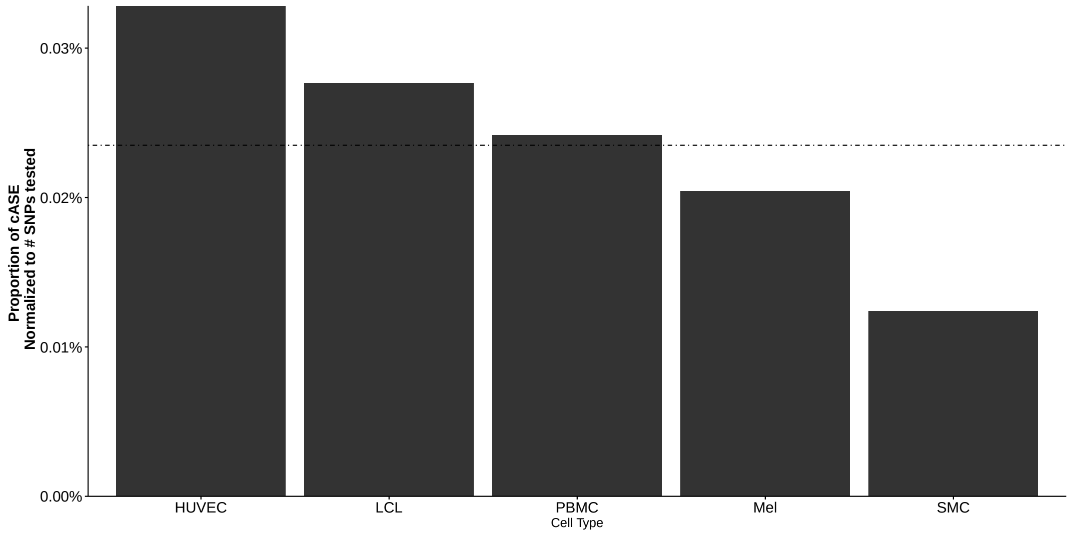

Figure S18: **Percent of cASE SNPs identified in each cell type.** For each cell type, plotted is the percent of cASE SNPs identified, relative to the number of SNPs tested for that group. The dotted black line represents the average percent of cASE SNPs across all categories or cell type. Groups with a ”*” are significantly enriched or depleted (Binomial p-value < 0.05) relative to the average.

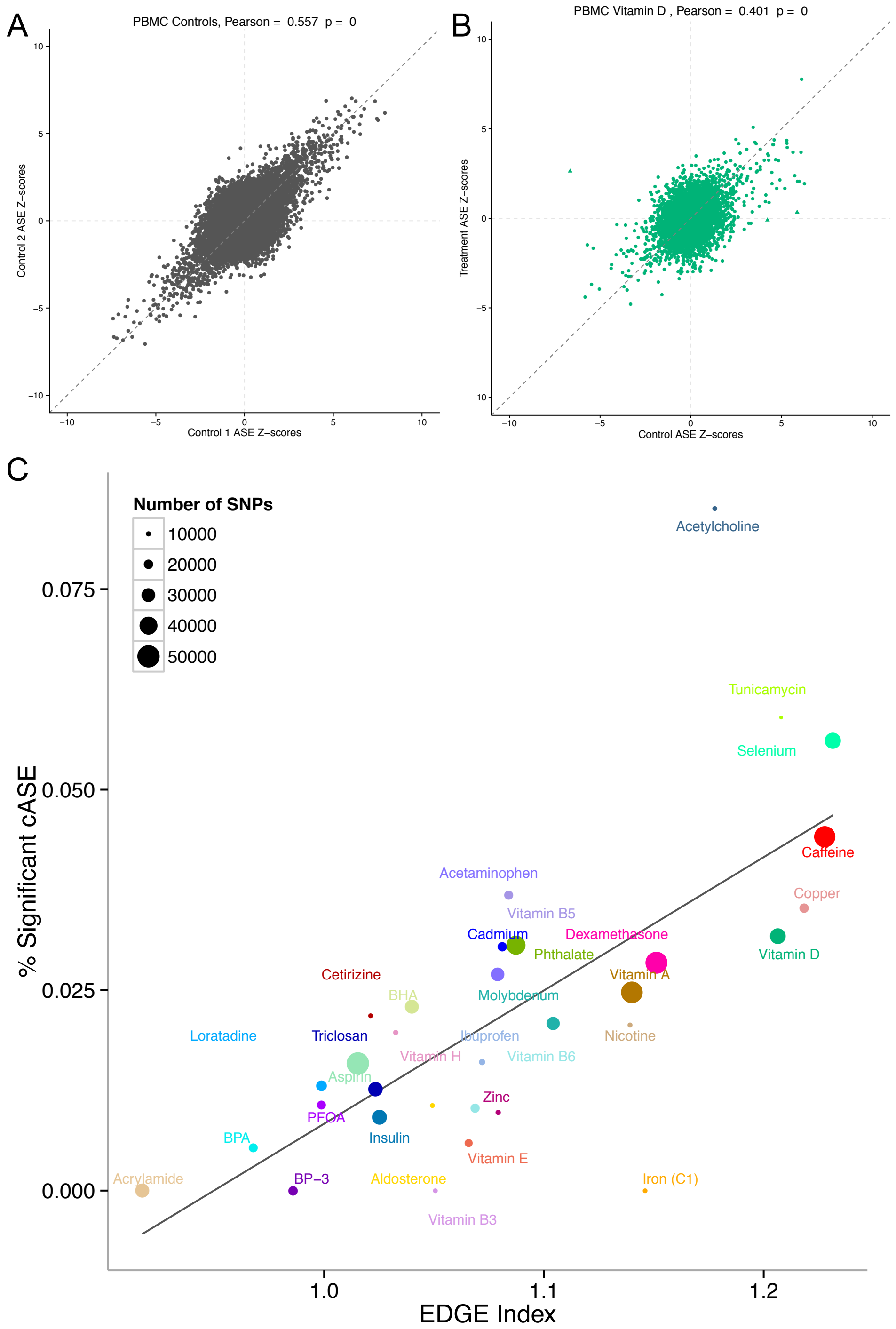

Figure S19: **Environmental displacement of genetic effect (EDGE) index.** (A & B) Example of EDGE. The low correlation of ASE Z-scores in (B) relative to the control in (A) highlights how the genetic effect is modulated by the treatment. In this example, the EDGE index would be calculated (using Equation 9) as 1 / (0.401 / 0.557) = 1.389. (C) For each treatment (indicated by color and label), plotted is the average EDGE Index across cell types versus the % cASE. The number of heterozygous SNPs tested for cASE in each treatment is indicated by the size of the point. The black line represents a linear model fit on the points, indicating the two are highly correlated (Spearman's $\rho = 0.717$, $p = 3.8 \times 10^{-6}$).

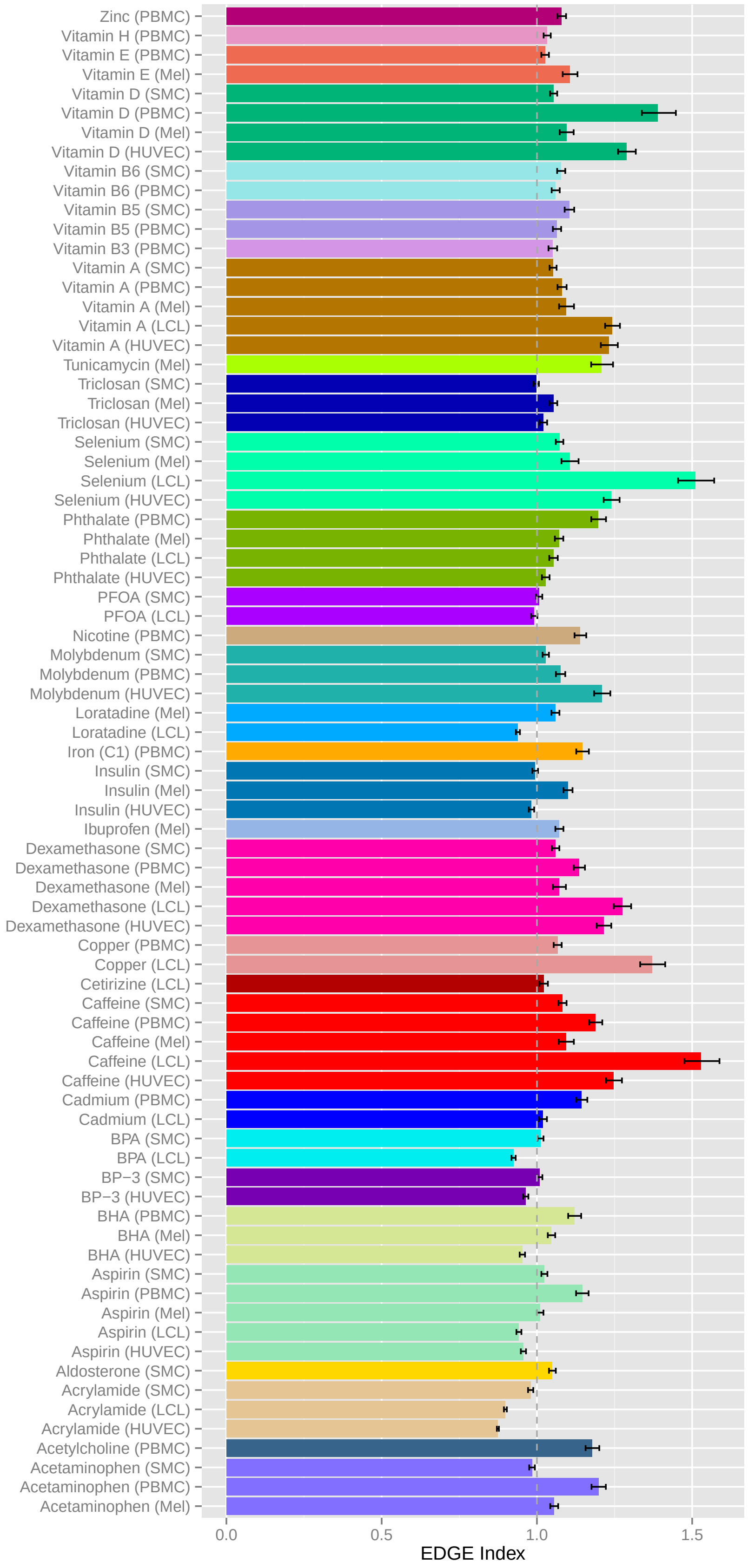

Figure S20: **Barplot of EDGE index values for each cell type and treatment.** Values are normalized within each cell type based on the controls (see Equation 9). Error bars indicate the 95 % confidence interval.

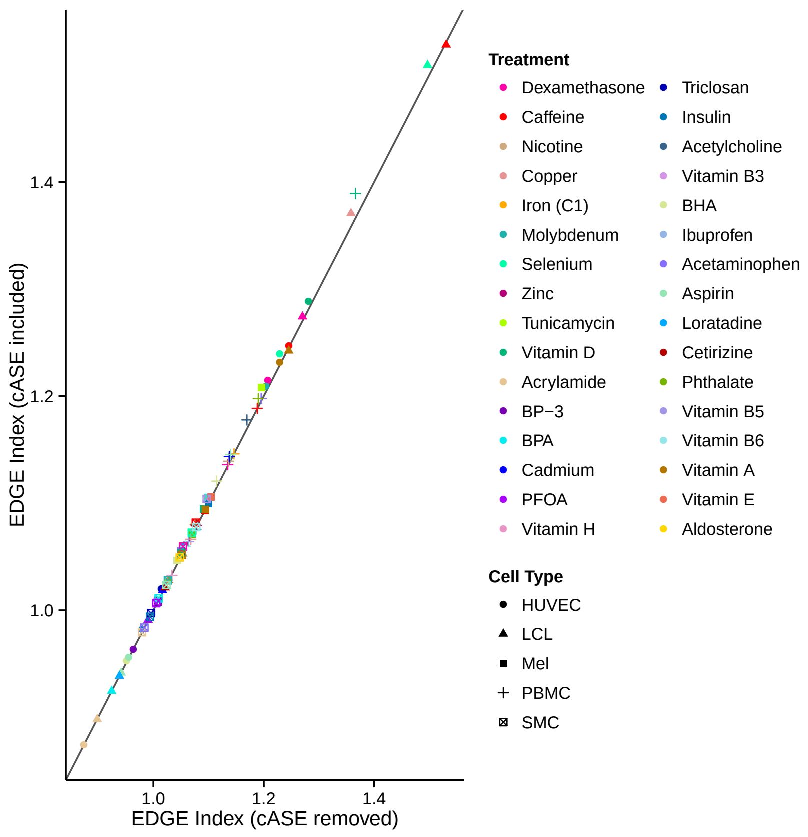

Figure S21: **Comparison of EDGE index values with and without including cASE SNPs.** For each sample, the EDGE index was calculated as described in Section 10.4, using either all SNPs in that subgroup (y-axis) or only SNPs that do not display significant cASE (x-axis). Note that for this analysis, we only included SNPs passing the default coverage filter (40 reads).
